# Insulin effects on core neurotransmitter pathways involved in schizophrenia neurobiology: a meta-analysis of preclinical studies. Implications for the treatment

**DOI:** 10.1038/s41380-023-02065-4

**Published:** 2023-04-21

**Authors:** Andrea de Bartolomeis, Giuseppe De Simone, Michele De Prisco, Annarita Barone, Raffaele Napoli, Francesco Beguinot, Martina Billeci, Michele Fornaro

**Affiliations:** 1 Section of Psychiatry, Laboratory of Molecular and Translational Psychiatry, Unit of Treatment-Resistant Psychiatric Disorders, Department of Neuroscience, Reproductive Sciences and Odontostomatology University of Naples “Federico II”, School of Medicine, Via Pansini 5, 80131 Naples, Italy; 2 Bipolar and Depressive Disorders Unit, Institute of Neuroscience, Hospital Clinic, University of Barcelona, IDIBAPS, CIBERSAM, 170 Villarroel st, 12-0, 08036 Barcelona, Catalonia Spain; 3 Department of Translational Medical Sciences, University of Naples “Federico II”, Via S. Pansini 5, 80131 Naples, Italy; 4 URT Genomic of Diabetes, Institute of Experimental Endocrinology and Oncology, National Research Council, Naples, Italy

**Keywords:** Neuroscience, Schizophrenia

## Abstract

Impairment of insulin action and metabolic dysregulation have traditionally been associated with schizophrenia, although the molecular basis of such association remains still elusive. The present meta-analysis aims to assess the impact of insulin action manipulations (i.e., hyperinsulinemia, hypoinsulinemia, systemic or brain insulin resistance) on glutamatergic, dopaminergic, γ-aminobutyric acid (GABA)ergic, and serotonergic pathways in the central nervous system. More than one hundred outcomes, including transcript or protein levels, kinetic parameters, and other components of the neurotransmitter pathways, were collected from cultured cells, animals, or humans, and meta-analyzed by applying a random-effects model and adopting Hedges’g to compare means. Two hundred fifteen studies met the inclusion criteria, of which 180 entered the quantitative synthesis. Significant impairments in key regulators of synaptic plasticity processes were detected as the result of insulin handlings. Specifically, protein levels of N-methyl-D-aspartate receptor (NMDAR) subunits including type 2A (NR2A) (Hedges’ *g* = −0.95, 95%C.I. = −1.50, −0.39; *p* = 0.001; *I*^2^ = 47.46%) and 2B (NR2B) (Hedges’*g* = −0.69, 95%C.I. = −1.35, −0.02; *p* = 0.043; *I*^2^ = 62.09%), and Postsynaptic density protein 95 (PSD-95) (Hedges’*g* = −0.91, 95%C.I. = −1.51, −0.32; *p* = 0.003; *I*^2^ = 77.81%) were found reduced in insulin-resistant animal models. Moreover, insulin-resistant animals showed significantly impaired dopamine transporter activity, whereas the dopamine D2 receptor mRNA expression (Hedges’*g* = 3.259; 95%C.I. = 0.497, 6.020; *p* = 0.021; *I*^2^ = 90.61%) increased under insulin deficiency conditions. Insulin action modulated glutamate and GABA release, as well as several enzymes involved in GABA and serotonin synthesis. These results suggest that brain neurotransmitter systems are susceptible to insulin signaling abnormalities, resembling the discrete psychotic disorders’ neurobiology and possibly contributing to the development of neurobiological hallmarks of treatment-resistant schizophrenia.

## Introduction

Dysregulation of insulin action has been linked to schizophrenia pathophysiology above and beyond the side effects of pharmacological treatments [1], as shown by the high prevalence of diabetes even before the introduction of antipsychotics in clinical practice [2] and in drug-naïve patients at first-episode psychosis [3]. Although divergent evidence has been reported in the literature [4–7], a recent meta-analysis showed a significant increase in fasting plasma glucose levels, plasma glucose levels 2 h after an oral glucose load, or insulin resistance in first-episode psychotic patients compared to controls, with heterogeneity across studies likely due to different lifestyles such as diet and smoking, population-specific polymorphisms, and differences in the definition of antipsychotic naïve [3]. Even if short-term antipsychotic therapy may represent a cofounding bias in the framework of insulin action dysregulation as a primitive feature of schizophrenia, the correlation of insulin resistance with polygenic risk score and treatment outcome in drug-naïve first-episode patients strongly supports the idea of a direct link between psychotic manifestations and metabolic disturbances [8]. Further, although some authors argued that atypical antipsychotics impact glucose sensitivity and induce insulin resistance even after a single dose [9], a major part of the studies point to a dose- and time-dependent effect of antipsychotics on glucose metabolism, with the greatest effect noted with 2nd generation antipsychotics such as clozapine and olanzapine [10–13]. The evidence of a common basis, including genetic variants and perinatal stressors, for schizophrenia and type 2 diabetes (T2D), has strengthened the hypothesis of a strong interaction between these two worldwide diffused disorders [14–16].

Specifically, the brain insulin pathway may exert a critical function in promoting central glucose uptake and regulating astrocytic glucose availability and morphology, synaptic plasticity, circuit connectivity, and neurotransmitter trafficking via the PI3K/Akt/mTOR pathway [17, 18]. Moreover, insulin signaling has been found crucial both in clozapine-induced Akt pathway activation [19] and raclopride-mediated D2 receptor blockade [20–22], considered relevant for the antipsychotic therapeutic mechanism. Although the exact mechanism by which insulin might affect brain function needs to be better elucidated, it has been shown that most of the insulin in the brain derives from circulating pancreatic insulin through a saturable transport across the blood–brain barrier (BBB) [23]. Several factors are involved in the regulation of insulin transport via the BBB as well as insulin signaling in the brain, including obesity, inflammation, glycemia, insulin resistance, levels of circulating triglycerides, and age [23, 24]. In this framework, brain insulin resistance may arise from low levels of insulin in the central nervous system or resistance at the receptor level [23, 25]. Of interest, brain insulin resistance may precede the onset of a full-blown diabetic state or represent an independent manifestation, as proved by the observation of a reduced response to ex vivo insulin stimulation in the hippocampal formation and the cerebellar cortex of patients affected by Alzheimer’s Disease without diabetes [26]. Thus, even though influenced by systemic metabolic disturbances, insulin dysfunction in the brain may be present also in the absence of peripheral diabetic or prediabetic states.

Nonetheless, a systematic analysis investigating the insulin regulation of neurotransmission processes with special regard to the putative impact on schizophrenia neurobiology has never been performed, leaving a gap in the current knowledge of the molecular mechanisms implicated. Hence, fundamental questions remain unanswered: i) What is the overall effect of insulin action perturbations on the main neurotransmitters and related molecular components involved in schizophrenia? ii) Where do these effects mainly take place? iii) How do the changes in insulin function impact synaptic structures and proteins involved in schizophrenia neurobiology? With these questions in mind, we have launched a meta-analysis, focusing on multiple patterns of insulin manipulations and their effect on dopaminergic, glutamatergic, serotonergic, and γ-aminobutyric acid (GABA)ergic pathways, in the attempt to possibly shed light on the molecular bases of schizophrenia and metabolic disturbances under a translational perspective.

## Methods

The present systematic review and meta-analysis followed the Preferred Reporting Items for Systematic Reviews and Meta-Analyses (PRISMA) guidelines (please, see Supplementary Information for PRISMA checklist) [27]. The screening, data extraction, and methodological quality appraisal of eligible studies were independently performed by three investigators (MB, MDP, GDS). Any disagreements were solved by consensus. Two senior investigators decided when an agreement could not be reached (AdB, MF). An a priori written study protocol is available upon request.

### Search strategy

The PubMed/MEDLINE, Embase, and Scopus databases were systematically searched for references indexed from inception until July 1st, 2021. An additional search was launched on the PubMed/MEDLINE database for the identification of further studies published from July 1st, 2021 until June 30th, 2022. Search strings for the databases are available in Supplementary information. In addition, relevant cross-references, textbooks, and other materials were hand-searched to identify potential additional references not captured in the original searches.

### Eligibility criteria and study outcomes

To highlight changes in neurotransmitter pathways resulting from manipulations of the insulin action, peer-reviewed studies, published in any language, that provided quantitative data were deemed for inclusion. Eligible studies included *cultured cells*, *animals*, or *humans* with documented alterations in the insulin pathway. Controls shared the same characteristics of the cases but without the insulin action alteration. Any outcome related to dopaminergic, glutamatergic, serotonergic, and GABAergic pathways measured in the brain was considered for inclusion. Studies conducted in vivo, in vitro, and ex vivo were evaluated for inclusion. Studies i) that did not provide a control group nor quantitative data; ii) measuring neurotransmitter outcomes in tissues different from the brain; iii) combining multiple insulin alterations in the same subjects were excluded. Studies using paired samples (i.e., the same group pre- and post-treatment) as control groups were excluded, along with reviews, case reports, letters to editors, and commentaries.

### Data extraction

The following variables were extracted (when feasible): first author, year of publication, geographical region, country, study design, study type (in vivo, in vitro, ex vivo), species considered (i.e., human, animal), animal type, animal weight, insulin action alteration type (hyperinsulinemia, hypoinsulinemia, peripheral or brain insulin resistance), experimental paradigm details for cases and controls (i.e., type of intervention, agent doses), neurotransmitter outcome type and its unit of measure, brain area considered, mean time from insulin alteration to neurotransmitter measure, blood glucose and insulin plasma levels for cases and controls, mean age, % of female, sample size, outcome mean and standard deviation (SD) for both cases and controls. If the study met our a priori-defined inclusion criteria, but raw data were not fully available, the computed effect size was used; if no effect size was available, the authors were contacted twice to ask for data. For studies reporting data in figures only, WebPlotDigitizer (https://automeris.io/WebPlotDigitizer/) was used to extract data from figures manually.

### Methodological quality appraisal

The methodological quality of included studies was assessed by using the Systematic Review Centre for Laboratory Animal Experimentation (SYRCLE) Tool for animal studies and the National Institutes of Health (NIH) Study Quality Assessment Tools for human studies (Supplementary Tables 1 and 2).

### Statistical analysis

Individual study data were pooled using the DerSimonian-Laird proportion method [28] with Comprehensive Meta-Analysis® software (version 2) [29]; random-effects modeling was applied for meta-analytic estimates. Heterogeneity was assessed by using I^2^ and Tau^2^. When the number of comparisons (*k*) equaled three or more and high heterogeneity was detected (*I*^2^ ≥ 75%) [30], the following a priori planned subgroup analyses were conducted: study type, animal type, insulin alteration model, and brain area. Hedges’g was adopted for comparison between means. Publication bias was evaluated by looking at the funnel plot and using the trim and fill method; Egger’s test was adopted to assess the eventual publication bias. Different animal species were analyzed together and subgroup analysis according to the animal type was always accomplished. However, we decided not to analyze together data coming from human and animal studies due to the expected variability in study methodology and organism biology. Meta-regression analyses were performed for the following variables: time from insulin alteration to neurotransmitter measure, blood glucose and insulin plasma concentration, and homeostatic model assessment (HOMA) index, expressed as the ratio between the levels in cases and controls. Meta-regression analyses were performed exclusively in the same animal species whenever at least ten primary comparisons were available. Sensitivity analyses were carried out excluding one study at a time to identify possible outliers, which could be biasing the pooled effect size estimate. RStudio R version 4.1.2 [31] was adopted to render high-resolution plots.

## Results

Five thousand nine hundred thirty-five records were identified across different sources, yielding 3681 unique references (i.e., after excluding duplicated references) for further screening. Of the latter, 3073 studies were excluded at the title/abstract level, and other 393 records were excluded after full-text review.

**Fig. 1:**
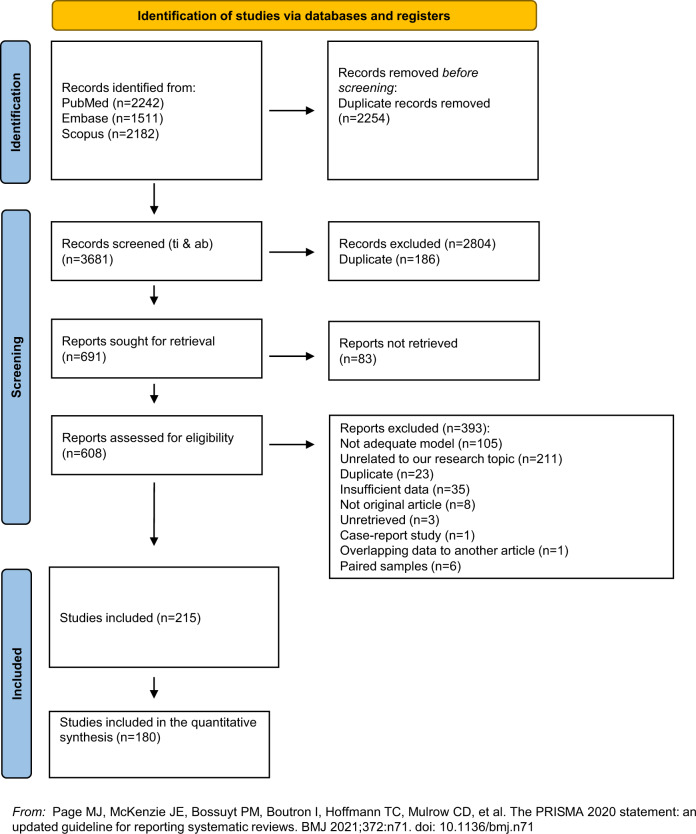
PRISMA flow diagram. Preferred Reporting Items for Systematic Reviews and Meta-Analyses (PRISMA) study selection flow diagram.

**Fig. 2:**
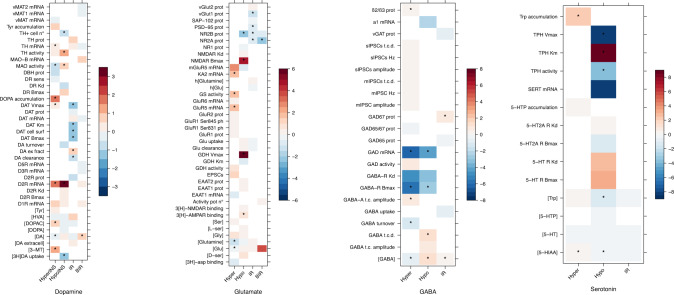
Effect sizes (Hedges’g) heatmap. Heatmap illustrating effect sizes (Hedges’g) of each outcome in different models (hyperinsulinemia, hypoinsulinemia, insulin resistance, and brain-limited insulin resistance) grouped into neurotransmitter pathways. The heatmap scale of colors codes the overall effect size (Hedges’g) value (ranging from blue to red as shown in the legend) obtained from each meta-analysis. Red colors implicate higher levels of the outcome in cases whereas blue colors implicate higher levels of outcome in controls. * is used to express a significant result (p<0.05). [3H]-asp binding [3H]-d-aspartate binding, [3H]DA uptake [3H]dopamine uptake, [3-MT] 3-methoxytyramine concentration, [5-HIAA] 5-hydroxyindoleacetic acid concentration, [5-HT] serotonin concentration, [5-HTP] 5-hydroxytryptophan concentration, [DA extracellular] extracellular dopamine concentration, [DA] dopamine concentration, [DOPA] dihydroxyphenylalanine concentration, [DOPAC] 3,4- dihydroxyphenylacetic acid concentration, [D-ser] D-serine concentration, [GABA] gamma-aminobutyric acid concentration, [Glu] glutamate concentration, [Glutamine] glutamine concentration, [Gly] glycine concentration, [HVA] homovanillic acid concentration, [L-ser] L-serine concentration, [Ser] serine concentration, [Trp] tryptophan concentration, [Tyr] tyrosine concentration, 3[H]-AMPAR binding 3[H]-AMPA receptor binding, 3[H]-NMDAR binding 3[H]-NMDA receptor binding, 5-HT R Bmax serotonin receptor maximal binding capacity, 5-HT R Kd serotonin receptor dissociation constant, 5-HT2AR Bmax serotonin receptor 2 A maximal binding capacity, 5-HT2A R Kd serotonin receptor 2A dissociation constant, 5-HTP accumulation 5-hydroxytryptophan accumulation, activity pot n° action potential number, D1R mRNA dopamine D1 receptor mRNA, D2R Bmax dopamine D2 receptor maximal binding capacity, D2R Kd dopamine D2 receptor dissociation constant, D2R prot dopamine D2 receptor protein levels, D3R mRNA dopamine D3 receptor mRNA, D5R mRNA dopamine D5 receptor mRNA, DA clearance dopamine clearance, DA ex fract dopamine extraction fraction, DA turnover dopamine turnover, DAT Bmax dopamine transporter maximal binding capacity, DAT cell surf dopamine transporter cell surface expression, DAT Km dopamine transporter Michaelis constant, DAT mRNA dopamine transporter mRNA, DAT prot dopamine transporter protein levels, DAT Vmax dopamine transporter maximal velocity, DOPA accumulation dihydroxyphenylalanine accumulation, DR Bmax dopamine receptor maximal binding capacity, DR Kd dopamine receptor dissociation constant, DR sens dopamine receptor sensitivity, DβH prot dopamine-β-hydroxylase protein levels, EAAT1 mRNA excitatory amino acid transporter type 1 mRNA, EAAT1 prot excitatory amino acid transporter type 1 protein levels, EAAT2 prot excitatory amino acid transporter type 2 protein levels, EPSCs excitatory postsynaptic currents, GABA t.c. amplitude GABA tonic current amplitude, GABA t.c.d. GABA tonic current density, GABA-A t.c. amplitude GABA A receptor tonic current amplitude, GABA-R Bmax GABA receptor maximal binding capacity, GABA-R Kd GABA receptor dissociation constant, GAD activity glutamic acid decarboxylase activity, GAD mRNA glutamic acid decarboxylase mRNA, GAD-65 prot glutamic acid decarboxylase 65 kDa form protein levels, GAD65/67 prot glutamic acid decarboxylase 65/67 kDa form protein levels, GAD67 prot glutamic acid decarboxylase 67 kDa form protein levels, GDH activity glutamate dehydrogenase activity, GDH Km glutamate dehydrogenase Michaelis constant, GDH Vmax glutamate dehydrogenase maximal velocity, Glu clearance glutamate clearance, Glu uptake glutamate uptake, GluR1 prot glutamate ionotropic receptor AMPA type subunit 1, GluR1 Ser831 ph glutamate ionotropic receptor AMPA type subunit 1 phosphorylation at Ser831, GluR1 Ser845 ph glutamate ionotropic receptor AMPA type subunit 1 phosphorylation at Ser845, GluR2 prot glutamate ionotropic receptor AMPA type subunit 2 protein levels, GluR5 (GRIK1) glutamate ionotropic receptor kainate type 1 subunit, GluR6 (GRIK2) mRNA glutamate ionotropic receptor kainate type 2 subunit mRNA, GS activity glutamine synthase activity, h[Glu] human glutamate concentration, h[Glutamine] human glutamine concentration, KA2 (GRIK5) mRNA glutamate ionotropic receptor kainate type 5 subunit mRNA, MAO activity monoamine oxidases activity, MAO-B mRNA monoamine oxidase B mRNA, mGluR5 mRNAglutamate metabotropic receptor type 5 mRNA, mIPSC amplitude miniature inhibitory postsynaptic currents amplitude, mIPSC Hz miniature inhibitory postsynaptic currents frequency, mIPSCs t.c.d.miniature inhibitory postsynaptic currents total current density, NMDAR Bmax NMDA receptor maximal binding capacity, NMDAR Kd NMDA receptor dissociation constant, NR1 prot glutamate ionotropic receptor NMDA type subunit 1 protein levels, NR2A prot glutamate ionotropic receptor NMDA type subunit 2A protein levels, NR2B  glutamate ionotropic receptor NMDA type subunit 2B protein levels, PSD-95 prot postsynaptic density protein 95 protein levels, SAP-102 prot synapse associated protein 102 protein levels, SERT mRNA serotonin transporter mRNA, sIPSCs amplitude spontaneous inhibitory postsynaptic currents amplitude, sIPSCs Hz spontaneous inhibitory postsynaptic currents frequency, sIPSCs t.c.d. spontaneous inhibitory postsynaptic currents total current density, TH activity tyrosine hydroxylase activity, TH mRNA tyrosine hydroxylase mRNA, TH prot tyrosine hydroxylase protein levels, TH + cell n number of tyrosine hydroxylase + cells, TPH activity tryptophan hydroxylase activity, TPH Km tryptophan hydroxylase Michaelis constant, TPH Vmax tryptophan hydroxylase maximal velocity, Trp accumulation tryptophan accumulation, Tyr accumulation tyrosine accumulation, vGAT prot vesicular GABA transporter protein levels, vGlut1 prot vesicular glutamate transporter 1 protein levels, vGlut2 prot vesicular glutamate transporter 2 protein levels, vMAT mRNA vesicular monoamine transporter mRNA, vMAT1 mRNA vesicular monoamine transporter 1 mRNA, vMAT2 mRNA vesicular monoamine transporter 2 mRNA, α1 mRNA GABA-A receptor α1 subunit mRNA, β2/β3 prot GABA-A receptor β2/β3 subunits protein levels.

Two hundred fifteen studies were deemed eligible for inclusion in the systematic review. One hundred eighty studies could be meta-analyzed. Almost all of the included studies (208) were conducted on animals or cultured cells, while only seven reports were performed on humans. The details of the selection process are displayed in Fig. 1. Due to the extensive data extracted, a detailed analysis of all the significant outcomes has been reported in the supplementary materials. The synthesis of overall the computed effect sizes appears in the heatmap in Fig. 2. Please, see the Supplementary Information section for a full consultation of forest plots, publication bias, meta-regressions, and subgroup analyses.

The data were systematized according to the different types of alteration induced or observed in the insulin action and thus were organized into four distinct groups: *hyperinsulinemia, hypoinsulinemia, insulin resistance*, and *brain insulin resistance*. The specific characteristics of each model are discussed in the Supplementary Information. It should be noted that spontaneous hyperinsulinemia, being a compensatory mechanism preventing the development of hyperglycemia in insulin-resistant individuals, can be considered a marker of insulin resistance in human clinical studies. However, this assumption cannot be extended to preclinical animal models explored in the present meta-analysis, since in all the included studies, hyperinsulinemia was induced acutely, often by insulin or tolbutamide administration, and was employed to reproduce hypoglycaemic conditions. Moreover, we differentiated insulin deficiency conditions caused, among others, by streptozotocin or alloxan treatment and comprised in the hypoinsulinemic model from peripheral and brain insulin resistance. Overall, hyperinsulinemic models were adopted to identify conditions characterized by increased insulin signaling, generally attributable to an elevation of peripheral and brain insulin levels. Otherwise, hypoinsulinemia was used to define conditions of reduced insulin signaling due to low insulin levels, in contrast to insulin-resistant models in which, normal levels of insulin were coupled with impaired transductive mechanisms. In addition, when reported, the comparison of insulin and glucose levels between cases and controls was used to ensure more correct assignment to individual models. The preclinical studies included in the present meta-analysis did not involve concurrent administration of antipsychotic therapy. Therefore, alterations in neurotransmitter pathways are related to insulin manipulation and not to other confounding variables. All the studies conducted in humans provided evidence by neuroimaging techniques.

### Glutamatergic outcomes

**Fig. 3:**
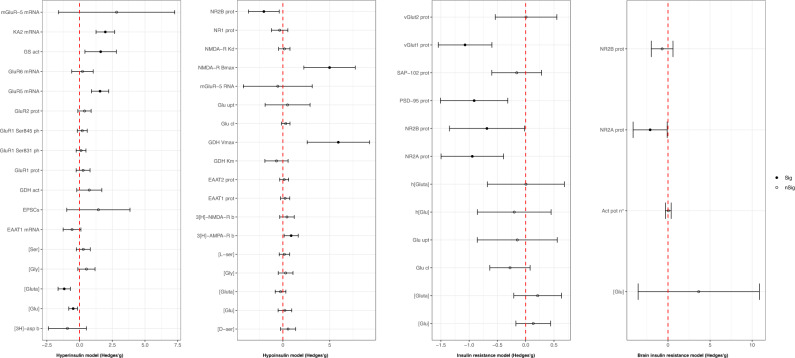
Glutamatergic outcomes effect sizes (Hedges’g) and 95% confidence intervals. Effect size and 95% confidence intervals are provided for each outcome included in the glutamatergic pathway and grouped into hyperinsulinemic, hypoinsulinemic, insulin-resistant, and brain insulin-resistant models respectively. [3H]-asp b [3H]-d-aspartate binding, [3H]-NMDA-R b [3H]-NMDA receptor binding, [3H]-AMPA-R b [3H]-AMPA receptor binding, [Glu] glutamate concentration, [Gluta] glutamine concentration, [Gly] glycine concentration, [D-ser] D-serine concentration, [L-ser] L-serine concentration, [Ser] serine concentration, act pot n° activity potential number, EAAT1 mRNA excitatory amino acid transporter type 1 mRNA, EAAT1 prot excitatory amino acid transporter type 1 protein levels, EPSCs excitatory postsynaptic currents, GDH act glutamate dehydrogenase activity, GDH Km glutamate dehydrogenase Michaelis constant, GDH Vmax glutamate dehydrogenase maximal velocity, Glu cl glutamate clearance, Glu upt glutamate uptake, GluR1 prot glutamate ionotropic receptor AMPA type subunit 1 protein levels, GluR1 Ser831 ph glutamate ionotropic receptor AMPA type subunit 1 phosphorylation at Ser831, GluR1 Ser845 ph glutamate ionotropic receptor AMPA type subunit 1 phosphorylation at Ser845, GluR2 prot glutamate ionotropic receptor AMPA type subunit 2 protein levels, GluR5 (GRIK1)  glutamate ionotropic receptor kainate type 1 subunit, GluR6 (GRIK2) mRNA glutamate ionotropic receptor kainate type 2 subunit mRNA, GS act glutamine synthase activity, h[Glu] human glutamate concentration, h[Gluta] human glutamine concentration, KA2 (GRIK5) mRNA glutamate ionotropic receptor kainate type 5 subunit mRNA, mGluR-5 mRNA metabotropic glutamate receptor type 5 subunit mRNA, NMDA-R Bmax NMDA receptor maximal binding capacity, NMDA-R Kd NMDA receptor dissociation constant, NR1 prot glutamate ionotropic receptor NMDA type subunit 1 protein levels, NR2A prot glutamate ionotropic receptor NMDA type subunit 2A protein levels, NR2B prot glutamate ionotropic receptor NMDA type subunit 2B protein level, PSD-95 prot postsynaptic density protein 95 protein levels, SAP-102 prot synapse associated protein 102 protein levels, vGlut1 prot vesicular glutamate transporter 1 protein levels, vGlut2 prot vesicular glutamate transporter 2 protein levels.

As reported in Fig. 3, hyperinsulinemic animals exhibited reduced brain glutamate (Hedges’*g* = −0.49; 95%C.I. = −0.83, −0.16; *p* = 0.004; *I*^2^ = 82.29%; based on 71 comparisons from 17 animal interventional studies [32–48]) and glutamine concentrations (Hedges’*g* = −1.17; 95%C.I. = −1.64, −0.70; *p* < 0.001; *I*^2^ = 84.27%; based on eight studies [34, 37–39, 45–48] fetching 41 comparisons). Subgroup analysis among studies exploring glutamate concentration showed a significant reduction in ex vivo studies and a significant increase in studies conducted in vitro and in vivo; the study type did not affect glutamine concentration. Animals in the hypoinsulinemic group showed a significant increase in both ^3^[H]- α-amino-3-hydroxy-5-methyl-4-isoxazole-propionic acid receptor (AMPAR) binding (Hedges’*g* = 0.89; 95%C.I. = 0.13, 1.65; *p* = 0.022; *I*^2^ = 73.11%; based on one study [49] providing nine comparisons) and N-methyl-D-aspartate receptor (NMDAR) *B*_max_ (Hedges’*g* = 4.99; 95%C.I. = 2.24, 7.74; *p* < 0.001; *I*^2^ = 79%; based on three studies [43, 49, 50] fetching three comparisons), and a significant reduction in the hippocampal protein levels of NMDAR type subunit 2B (NR2B) (Hedges’*g* = −2.03, 95%C.I. = −3.67, −0.39; *p* = 0.015; *I*^2^ = 79.79%; based on two studies [51, 52] fetching four comparisons). The insulin-resistant group displayed a significant drop in the hippocampal levels of several proteins involved in NMDAR function, including NMDAR type subunit 2A (NR2A) (Hedges’*g* = −0.95, 95%C.I. = −1.50, −0.39; *p* = 0.001; *I*^2^ = 47.46%; based on three studies [53–55] providing eight comparisons), NR2B (Hedges’*g* = −0.69, 95%C.I. = −1.35, −0.02; *p* = 0.043; *I*^2^ = 62.09%; based on three studies [53–55] fetching eight comparisons), and postsynaptic density protein 95 (PSD-95) (Hedges’*g* = −0.91, 95%C.I. = −1.51, −0.32; *p* = 0.003; *I*^2^ = 77.81%; based on 14 comparisons from four studies [55–57]). Even though data were insufficient to provide a meta-analysis, NMDAR type subunit 1 (NR1) [54] levels also decreased under insulin-resistant conditions. Animals in the brain insulin-resistant group presented a significant decline in the hippocampal protein levels of NR2A (Hedges’*g* = −2.11; 95%C.I. = −4.14, −0.09; *p* = 0.041; *I*^2^ = 88.00%; based on three studies [58–60] providing three comparisons). Although not significant, hippocampal protein levels of NR2B (Hedges’*g* = −0.69; 95%C.I. = −1.98, 0.60; *p* = 0.293; *I*^2^ = 78.02%; based on three studies [58, 60, 61] providing three comparisons) appeared to be reduced too. Even though data were not sufficient to provide a quantitative analysis, brain insulin-resistant animals showed a decrease in the hippocampal levels of NR1 [59, 60]. Animal type, study type, and brain area were effective in reducing heterogeneity for almost all the significant outcomes. Please, see Fig. 3 for a full acknowledgment of effect sizes and 95% confidence intervals related to glutamatergic pathway outcomes.

### Dopaminergic outcomes

**Fig. 4:**
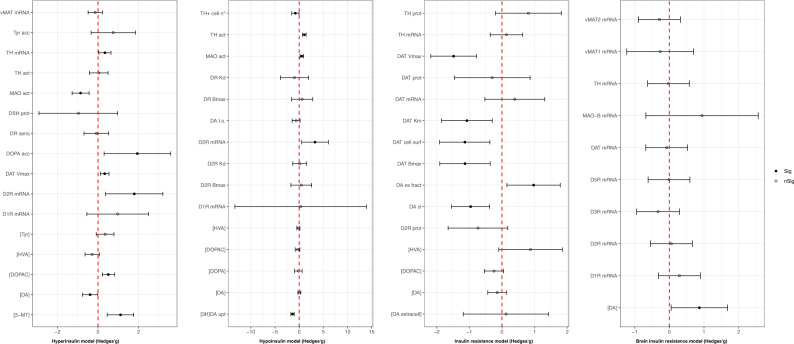
Dopaminergic outcomes effect sizes (Hedges’g) and 95% confidence intervals. Effect size and 95% confidence intervals are provided for each outcome included in the dopaminergic pathway and grouped into hyperinsulinemic, hypoinsulinemic, insulin-resistant, and brain insulin-resistant models respectively. [3-MT] 3-methoxytyramine concentration, [3H]DA upt [3H]dopamine uptake, [DA] dopamine concentration, [DA extracell] extracellular dopamine concentration, [DOPA] dihydroxyphenylalanine concentration, [DOPAC] 3,4-dihydroxyphenylacetic acid concentration, [HVA] homovanillic acid concentration, [Tyr] tyrosine concentration, D1R mRNA dopamine D1 receptor mRNA, D2R Bmax dopamine D2 receptor maximal binding capacity, D2R Kd dopamine D2 receptor dissociation constant, D2R mRNA dopamine D2 receptor mRNA, D2R prot dopamine D2 receptor protein levels, D3R mRNA dopamine D3 receptor mRNA, D5R mRNA dopamine D5 receptor mRNA, DA cl dopamine clearance, DA t.o. dopamine turnover, DAT Bmax dopamine transporter maximal binding capacity, DAT cell surf dopamine transporter cell surface expression, DAT ex fract dopamine transporter extraction fraction, DAT Km dopamine transporter Michaelis constant, DAT mRNA dopamine transporter mRNA, DAT prot dopamine transporter maximal protein levels, DAT Vmax dopamine transporter maximal velocity, DβH prot dopamine-β-hydroxylase protein levels, DOPA acc dihydroxyphenylalanine accumulation, DR Bmax dopamine receptor maximal binding capacity, DR Kd dopamine receptor dissociation constant, DR sens dopamine receptor sensitivity, MAO act monoamine oxidases activity, MAO-B mRNA monoamine oxidase B mRNA, TH act tyrosine hydroxylase activity, TH mRNA tyrosine hydroxylase mRNA, TH prot tyrosine hydroxylase protein levels, TH + cell n° number of cells tyrosine hydroxylase+, Tyr acc tyrosine accumulation, vMAT1 mRNA vesicular monoamine transporter 1 mRNA, vMAT2 mRNA vesicular monoamine transporter 2 mRNA.

Regarding dopaminergic pathway changes, shown in Fig. 4, we observed a significant decrease of dopamine concentrations in hyperinsulinemic animals (Hedges’*g* = −0.39; 95% C.I. = −0.77, −0.01; *p* = 0.043; *I*^2^ = 82.47%; based on 68 comparisons from 15 studies [62–76]) that was mirrored by an increase (Hedges’*g* = 0.87, 95% C.I. = 0.05, 1.69; *p* = 0.037; *I*^2^ = 44.29%; based on two studies [77, 78] fetching three comparisons) in brain insulin-resistant animals. In insulin-resistant animals, we found a significant impairment in functional parameters of the dopamine transporter (DAT), including DAT *B*_max_ (Hedges’*g* = −1.13, 95% C.I. = −1.90, −0.36; *p* = 0.004; *I*^2^ = 0%; based on one ex vivo study [79] fetching three comparisons), *V*_max_ (Hedges’*g* = −1.48, 95% C.I. = −2.18, −0.78; *p* < 0.001; *I*^2^ = 11.47%; based on one ex vivo study [79] fetching three comparisons), *K*_m_ (Hedges’*g* = −1.07, 95% C.I. = −1.85, −0.30; *p* = 0.007; *I*^2^ = 19.27%; based on one ex vivo study [79] fetching three comparisons), and DAT cell surface expression (Hedges’*g* = −1.14, 95% C.I. = −1.91, −0.37; *p* = 0.004; *I*^2^ = 24.76%; based on two ex vivo studies [79, 80] providing three comparisons), while a significant increase in DAT *V*_max_ (Hedges’*g* = 0.33; 95% C.I. = 0.12, 0.54; *p* = 0.002; *I*^2^ = 28.02%; based on one in vitro study [81] providing six comparisons) was observed in hyperinsulinemic animals. Dopamine clearance (Hedges’*g* = −1.48, 95% C.I. = −2.18, −0.78; *p* < 0.001; *I*^2^ = 11.47%; based on one in vivo study [80] fetching five comparisons) and [^3^H] dopamine uptake (Hedges’*g* = −1.371; 95% C.I. = −1.710, −1.032; *p* < 0.001; *I*^2^ = 0%; based on one study [82] providing four comparisons) displayed a significant reduction in insulin-resistant and hypoinsulinemic animals respectively. Monoamine oxidase (MAO) activity was significantly reduced (Hedges’*g* = −0.86; 95% C.I. = −1.28, −0.44; *p* < 0.001; *I*^2^ = 36.32%; based on one ex vivo study [66] fetching nine comparisons) and increased (Hedges’*g* = 0.545; 95% C.I. = 0.158, 0.904; *p* = 0.003; *I*^2^ = 0%; based on one study [66] providing eight comparisons) in hyper- and hypoinsulinemic conditions, respectively. Dopamine D2 receptor (D2R) D2R mRNA expression was increased both in hyper- (Hedges’*g* = 1.79; 95%C.I. = 0.37, 3.20; *p* = 0.013; *I*^2^ = 87.26%; based on seven comparisons from three studies [70, 74, 83]) and hypoinsulinemic (Hedges’*g* = 3.259; 95% C.I. = 0.497, 6.020; *p* = 0.021; *I*^2^ = 90.61%; based on three studies [70, 84, 85] providing three comparisons) animals. Please, see Fig. 4 for a full acknowledgment of effect sizes and 95% confidence intervals related to dopaminergic pathway outcomes.

### GABAergic outcomes

**Fig. 5:**
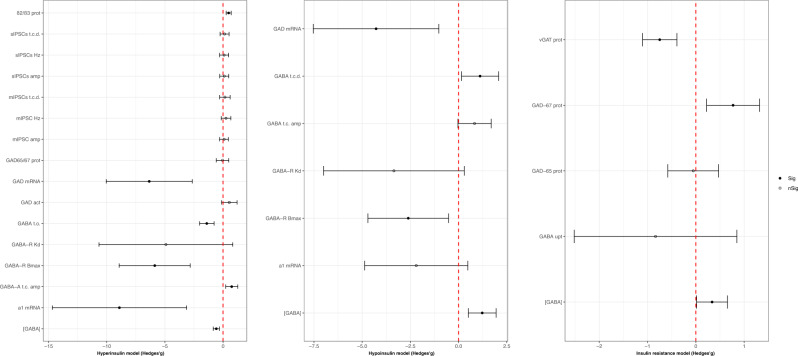
GABAergic outcomes effect sizes (Hedges’*g*) and 95% confidence intervals. Effect size and 95% confidence intervals are provided for each outcome included in the GABAergic pathway and grouped into hyperinsulinemic, hypoinsulinemic, and insulin-resistant respectively. α1 GABA-A receptor α1 subunit mRNA, β2/β3 prot GABA-A receptor β2/β3 subunits protein levels, [GABA] GABA concentration, GABA t.c. amp GABA tonic current amplitude, GABA t.c.d. GABA tonic current density, GABA t.o. GABA turnover, GABA upt GABA uptake, GABA-A t.c. amp GABA-A receptor tonic current amplitude, GABA-R Bmax GABA receptor maximal binding capacity, GABA-R Kd GABA receptor dissociation constant, GAD act glutamic acid decarboxylase activity, GAD mRNA glutamic acid decarboxylase mRNA, GAD65 prot glutamic acid decarboxylase 65 kDa form protein levels, GAD65/67 prot glutamic acid decarboxylase 65/67 kDa form protein levels, GAD67 prot glutamic acid decarboxylase 67 kDa form protein levels, mIPSCs amp miniature inhibitory postsynaptic currents amplitude, mIPSCs Hz miniature inhibitory postsynaptic currents frequency, mIPSCs t.c.d. miniature inhibitory postsynaptic currents total current density, sIPSCs amp spontaneous inhibitory postsynaptic currents amplitude, sIPSCs Hz spontaneous inhibitory postsynaptic currents frequency, sIPSCs t.c.d. spontaneous inhibitory postsynaptic currents total current density, vGAT prot vesicular GABA transporter protein levels.

As shown in Fig. 5, hyperinsulinemic animals showed lower glutamic acid decarboxylase (GAD) mRNA (Hedges’*g* = −6.35; 95% C.I. = −10.05, −2.64; *p* = 0.001; *I*^2^ = 83.59%; based on three comparisons from three ex vivo studies [85–87]) and higher GABA-A receptor β2/β3 subunits (Hedges’*g* = 0.48; 95% C.I. = 0.27, 0.70; p < 0.001; *I*^2^ = 17.51%; based on one in vitro study [88] providing five comparisons). GABA concentration displayed a significant reduction in hyperinsulinemia (Hedges’*g* = −0.58; 95% C.I. = −0.85, −0.32; *p* < 0.001; *I*^2^ = 75.09%; based on 70 comparisons from 14 studies [32, 37, 38, 41, 42, 44–47, 89–93]) and an increase in hypoinsulinemic (Hedges’ *g* = 1.37, 95%C.I. = 0.45, 2.29; *p* = 0.004; *I*^2^ = 83.60%; based on 12 comparisons from six studies [94–99]) and insulin-resistant animals (Hedges’*g* = 0.34, 95% C.I. = 0.02, 0.66; *p* = 0.04; *I*^2^ = 0%; based on two studies [57, 100] providing eight comparisons). Please, see Fig. 5 for a full acknowledgment of effect sizes and 95% confidence intervals related to GABAergic pathway outcomes.

### Serotonergic outcomes

**Fig. 6:**
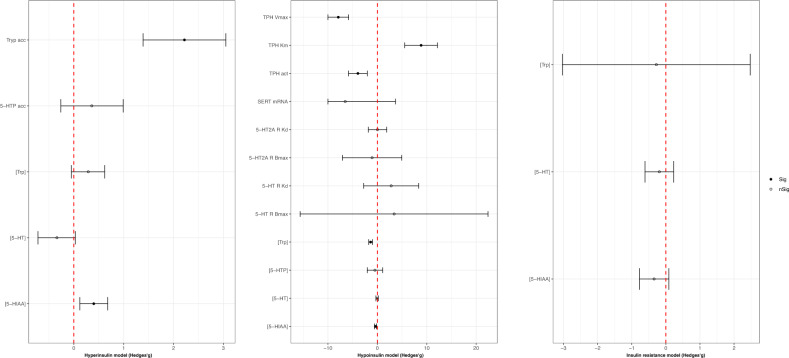
Serotonergic outcomes effect sizes (Hedges’g) and 95% confidence intervals. Effect size and 95% confidence intervals are provided for each outcome included in the serotonergic pathway and grouped into hyperinsulinemic, hypoinsulinemic, and insulin-resistant models respectively. In the hypoinsulinemic model, TPH V max and SERT mRNA values have been scaled for easier viewing; the real values have been reported in the supplementary materials. [5-HIAA] 5-hydroxy indole acetic acid concentration; [5-HT] serotonin concentration; [5-HTP] 5-hydroxytryptophan concentration; [Trp] tryptophan concentration; 5-HT R Bmax serotonin receptor maximal binding capacity; 5-HT R Kd serotonin receptor dissociation constant; 5-HT2A R Bmax serotonin receptor 2 A maximal binding capacity; 5-HT2A R Kd serotonin receptor 2A dissociation constant; 5-HTP acc 5-hydroxytryptophan accumulation; SERT mRNA serotonin transporter mRNA; TPH act tryptophan hydroxylase activity; TPH Km tryptophan hydroxylase Michaelis constant; TPH Vmax tryptophan hydroxylase maximal velocity; Tryp acc tryptophan accumulation.

With the regard to the serotonergic pathway, we observed a significant reduction in tryptophan (Trp) concentration (Hedges’*g* = −1.35; 95% C.I. = −1.75; −0.96, *p* < 0.001; *I*^2^ = 63.92%, based on seven studies [75, 101–106] fetching 17 comparisons), Trp hydroxylase (TPH) activity rate (Hedges’*g* =−3.93, 95% C.I. = −5.83; −2.03; *p* < 0.001; *I*^2^ = 82.56%; based on two studies [104, 107] providing five comparisons), and TPH *V*_max_ (Hedges’*g* = −45.64; 95% C.I. = −57.74, −33.53; *p* < 0.001; *I*^2^ = 27.62%; based on one study [107] providing four comparisons) in hypoinsulinemic animals. Moreover, hyperinsulinemic animals displayed a significant increase in the concentration of 5-hydroxyindoleacetic acid (5-HIAA) (Hedges’*g* = 0.40; 95% C.I. = 0.12, 0.68; *p* = 0.005; *I*^2^ = 75.62%; based on 64 comparisons from 17 studies [62, 63, 65, 68, 72, 73, 75, 76, 108–116]) and Trp accumulation (Hedges’*g* = 2.22; 95% C.I. = 1.39, 3.05; *p* < 0.001; *I*^2^ = 41.79%; based on one ex vivo study [63] fetching five comparisons). Conversely, 5-HIAA levels were significantly reduced in the hypoinsulinemic model (Hedges’*g* = −0.37; 95% C.I. = −0.59, −0.16; *p* = 0.001; *I*^2^ = 73.97%; based on 71 comparisons from 21 studies [75, 95, 101–106, 117–129]). Please, see Fig. 6 for a full acknowledgment of effect sizes and 95% confidence intervals related to serotonergic pathway outcomes.

## Discussion

To the best of our knowledge, this is the first quantitative synthesis of molecular components belonging to the glutamatergic, dopaminergic, serotonergic, and GABAergic systems in response to perturbations of insulin action. We detected significant impairments as consequences of systemic and brain-selective insulin manipulations in animal models. These observations support a possible role for dysregulation of insulin action in the neurobiology of psychotic disorders [17–22] in which the above neurotransmitters have been implicated. In light of the data produced, not only might insulin resistance be seen as a relevant factor in the pathophysiology of schizophrenia, but also a determinant player in its treatment response and resistance.

### Insulin and glutamatergic pathway

NMDARs are ionotropic glutamate receptors involved in excitatory neurotransmission and synaptic plasticity, displaying a heterotetrameric assembly composed of two NR1 and two NR2 subunits, with PSD-95 as a major protein at PSD playing both a functional and structural role in their regulation [130]. In literature, the role of NMDAR in the pathogenesis of schizophrenia has been well characterized, pointing to the receptor hypofunction as a driver for the onset of psychotic symptoms [131–133], and novel direct or undirect receptor modulators are currently under development as potential therapeutic agents in schizophrenia and treatment-resistant schizophrenia (TRS) [134].

According to the results of the present meta-analysis, we observed a significant reduction in NR2A, NR2B, and PSD-95 hippocampal levels and a significantly higher NMDAR *B*_max_ in hypoinsulinemic or insulin-resistant animals consistent with the view of insulin manipulations leading to crucial changes in the glutamatergic pathways.

A decrease in PSD-95, a major protein at PSD playing both a functional and structural role in NMDAR regulation and allowing the multimerization and clustering of protein complexes within the postsynaptic density, may affect the ability to recruit receptors and enhance synaptic strength [135]. It should be noted that a reduction in hippocampal expression of PSD-95 has been observed in post-mortem brain tissue of schizophrenia patients [136]. Of interest, PSD-95 directly binds to the NR2A subunit of NMDAR, which in the present meta-analysis was found likewise reduced. Furthermore, since NR2B-containing NMDARs are mainly involved in plasticity, synapse activation, and circuit integration [137], the detected reduction in NR2B may account for the weakening of hippocampal synapses. In this respect, the increase in the receptor total density measured as binding availability (*B*_max_) could be instead explained as a compensatory mechanism to counterbalance the NMDAR hypofunction.

Hippocampal NMDAR hypofunction has been related to a higher glutamate release in schizophrenia [138, 139]. Although no significant increase in glutamate concentration was reported in preclinical models of insulin resistance, our analysis revealed a parallel reduction in glutamate and glutamine levels associated with hyperinsulinemia and a trend toward the significance of increasing glutamate levels in hypoinsulinemic and insulin-resistant models. These findings are further supported by the increase in glutamine synthetase activity observed in hyperinsulinemic animals that may reflect the reduction in end products of glutamine metabolism [140]. Moreover, the meta-analysis of glutamate and glutamine concentrations in human subjects with T2D or prediabetes did not provide significant results, probably due to the limited number of included studies [141–143]. However, a study of 40 adults with a negative history of psychiatric illness demonstrated decreased cortical plasticity in both T2D and prediabetic subjects compared with controls as measured by transcranial magnetic stimulation [141].

Taken together, the data suggest that the decrease in insulin action, due to either insulin resistance or experimentally induced hypoinsulinemia, is associated with disturbances in synaptic plasticity processes and hypofunction of the glutamate receptor machinery, and, combined with the changes in glutamine/glutamate concentrations shown in the hyperinsulinemic model, resembles those alterations observed in schizophrenia patients. Consistently, hyperinsulinemia was associated with an increased expression of glutamate ionotropic receptor kainate type subunit 5 (GRIK5 or KA2) and glutamate ionotropic receptor kainate type subunit 1 (GRIK1 or GLUR5), whose loss-of-function mutations have recently been identified in schizophrenia patients and shown to weaken the interaction with PSD-95 [144].

The neurobiological bases of TRS remain elusive, although significant growing evidence supports the involvement of the glutamatergic pathway. While striatal dopamine synthesis capacity, measured by 3,4-dihydroxy-6-[18F]fluoro-l-phenylalanine (18F-DOPA) positron emission tomography (PET), has been demonstrated to be increased in patients responsive to antipsychotics, it did not substantially differ between TRS patients and healthy controls [145, 146]. Conversely, a significant increase in glutamate levels has been detected in multiple brain regions and notably in the anterior cingulate cortex (ACC) of TRS patients compared to responders and healthy controls, as measured by ^1^H-magnetic resonance spectroscopy (H^1^-MRS) [147–151]. Moreover, in the attempt to shed light on the neurobiological background of TRS, a multicenter study proved at once both an elevation in ACC glutamate levels and no major striatal dopamine uptake in resistant patients compared to responsive ones [147]. In conclusion, glutamatergic dysregulation may represent a molecular pattern specific for TRS whereas changes in the dopaminergic function may account for psychotic symptoms in drug-responsive patients.

Additionally, the present analysis showed a significant rise in ^3^[H]-AMPAR binding in hypoinsulinemic animals. There might be various reasons for this increase. However, considering this finding and the reduction in NMDAR subunits, it is conceivable that reduced insulin signaling may replicate some of the molecular changes observed in animal models of long-term antipsychotic therapy. Specifically, Kruyer and colleagues have recently described that, beyond the D2R upregulation and the increased proportion of high-affinity D2R, glutamatergic dysregulations, including the insertion of AMPA receptors and an increased AMPAR/NMDAR ratio combined with the loss of D2R-dependent inhibitory postsynaptic currents, might represent the molecular underpinning of behavioral supersensitivity [152]. Otherwise, the significant elevation in 3[H]-AMPAR binding and also in the *V*_max_ of glutamate dehydrogenase observed in hypoinsulinemic models might follow a possible increase in glutamate concentrations, which was not remarkably detected in the current study [153].

Overall, albeit with caution, these results let us hypothesize that insulin dysregulation in the glutamatergic signaling may mimic some of the discrete features of psychotic disorders and may provide a fertile background for antipsychotic refractoriness, contributing possibly to TRS, and thus triggering a “double resistance” state, namely the resistance to both insulin and antipsychotics.

### Insulin and dopaminergic pathways

Over time, several lines of evidence have highlighted the dopamine involvement in the neurobiology of psychotic symptoms, and, among others, PET neuroimaging approaches have provided the most replicated in vivo findings. The observations of a significant surge in dopamine release after amphetamine administration, as well as an increase in dopamine synthesis capacity in psychotic patients compared to healthy controls, detected by PET imaging studies, strongly support the dopamine hypothesis of schizophrenia [154–156].

In the present meta-analysis, dopamine concentration showed a significant decrease in hyperinsulinemic animals and a significant increase in brain insulin-resistant animal models, especially in limbic and striatal regions, suggesting a possible role for insulin resistance in the onset or maintenance of psychotic symptoms via augmentation of mesolimbic dopaminergic signaling. In this regard, dysfunctions of the mesolimbic pathway, as in a condition of striatal hyperdopaminergia, have been associated with psychotic symptoms and cognitive impairment in patients with schizophrenia, possibly by affecting the salience of environmental stimuli, perceptions processing, reward-based learning, and brain functional connectivity [157, 158]. Of interest, this finding has recently been reported also in humans, by the detection of reduced synaptic dopamine levels in response to central insulin stimulation as evidenced by a higher [11C]-raclopride binding potential at the striatal level [159]. The further observation of increased 3,4-dihydroxyphenylacetic acid and 3-methoxytyramine (3-MT) concentrations, DOPA accumulation, and tyrosine hydroxylase (TH) mRNA under conditions of hyperinsulinemia suggested a possible impairment of both dopamine synthesis and catabolism as the putative mechanism responsible for the altered dopamine levels [160].

Furthermore, we found significant impairment of DAT function in the striatum of insulin-resistant animals, with a decrease in DAT *B*_max_, *K*_m_, *V*_max_, and cell surface expression. Recently, a lower DAT expression has been found in the dorsal striatum of post-mortem tissue extracted from patients diagnosed with schizophrenia compared to controls, accountable for a striatal hyperdopaminergic state [161]. Although the reduction in DAT levels could be interpreted as the result of chronic drug administration in patients with schizophrenia, the impairment observed in insulin-resistant antipsychotic-free animals may suggest a possible involvement of insulin perturbation in the complex regulation of dopamine transporter [79, 80]. Thus, insulin signaling alterations may impair dopamine reuptake via dysregulation of DAT expression/function [161] and enhance synaptic dopamine release as endorsed by our observation of a significant decrease in dopamine clearance and [^3^H] dopamine uptake and supported by a remarkable increase in dopamine extraction fraction in insulin-resistant/hypoinsulinemic animals. Moreover, the reduction and elevation observed in MAO activity in hyper- and hypoinsulinemic conditions respectively may serve as a mechanism to balance dopamine levels, given the specific role in intracellular monoamine metabolization exerted by MAO enzymes [162].

The increase of D2R mRNA detected in both hyperinsulinemic and hypoinsulinemic animals included in the present meta-analysis may be explained by the different patterns of brain regions explored. Specifically, D2R expression was significantly increased in the striatum of hypoinsulinemic animals whereas in hyperinsulinemic models a quantitative analysis in this region of interest was not performed. Although changes in D2R protein levels apparently did not follow insulin-induced changes in D2R mRNA expression, a trend toward significance was detected in insulin-resistant animals. Further studies will be needed to assess whether the insulin pathway may be involved in modulating D2R protein levels over transcript regulation or not. However, it should be considered that insulin alterations may prime the dopaminergic system and facilitate dopamine sensitization in regions involved in schizophrenia pathophysiology, such as the striatum [84]. The role of striatal D2R has been well-defined in its association with schizophrenia neurobiology [163]. The upregulation of striatal D2R has been identified as a specific feature of supersensitivity psychosis, accounting for the relapse of psychotic symptoms and the development of a resistance condition by hindering effective blockade of D2Rs [164]. Moreover, the subchronic administration of haloperidol, a strong D2R blocker, has been associated with a striatal increase in the gene expression of multiple postsynaptic clustering regulators such as Homer1a/b/c [165]. Thus, insulin dysregulation by increasing striatal D2R expression may replicate the neurobiological correlates of supersensitivity psychosis and exhibit an inverse pattern of postsynaptic modifications compared to those elicited by antipsychotics, accounting for possible detrimental effects on neuronal plasticity.

### Insulin effects on GABA and serotonergic pathways

GABA concentration resulted significantly decreased under hyperinsulinemia and oppositely increased in hypoinsulinemic/insulin-resistant animals as detected in several brain areas, especially in the hypothalamus. Although decreased GABA levels in the midcingulate cortex and reduced GAD67 expression in the dorsolateral prefrontal cortex have been associated with schizophrenia pathophysiology by MRS and post-mortem studies [166–168], in the current meta-analysis cortical GABA concentrations were significantly different from controls only in the hyperinsulinemic model whereas no significant change was detected in the cortex of hypoinsulinemic and insulin-resistant animals.

Moreover, GABA concentration might mirror changes in glutamate levels as shown by the significant increase in the protein levels of GAD67, the rate-limiting enzyme catalyzing the conversion of glutamate to GABA [167], measured in the hippocampus of insulin-resistant animals.

Similarly, the reduction in GABA binding and turnover observed under hyperinsulinemic conditions could reflect changes in GABA concentrations [86] whereas the mismatch between GABA receptor *B*_max_ values as well as GAD mRNA levels in hypoinsulinemic and hyperinsulinemic models could be the consequence of neuronal injuries triggered by glucose homeostasis dysregulation, as suggested by the authors [87].

In addition, insulin concentrations were found to modulate the hippocampal levels of GABA receptor type A β_2_/β_3_ subunits, whose single nucleotide polymorphisms have been associated with cognitive function in schizophrenia patients [169].

Thus, further studies are needed to better clarify the GABAergic involvement under manipulations of insulin action apart from changes in glutamate levels and neuronal damage, focusing also on receptor modifications and possible different regional patterns of expression.

In the present meta-analysis, a significant reduction in the concentration of 5-HIAA and Trp was detected in hypoinsulinemic animals, as well as an alteration in TPH function, witnessed by a decrease in TPH activity and Vmax and an elevation in TPH *K*_m_ [170], whereas a significant increase in Trp accumulation and 5-HIAA levels was found in the hyperinsulinemic model. These findings support the insulin role in the regulation of Trp brain availability [171] and thus, the presumptive downregulation of the Trp pathway, at least toward serotonin and 5-HIAA formation, under insulin deficiency conditions.

Of interest, three main different metabolites have been found to originate directly from the Trp pathway: indole and 5-hydroxyindole (5-HI); kyurenine with its metabolites kynurenic acid (KYNA) and quinolinic acid; serotonin (5-HT) and 5-HIAA via TPH, the rate-limiting enzyme for 5-HT biosynthesis, activity [172].

In schizophrenia patients, 5-HI levels have recently been positively associated with the cortical thickness of the left lateral orbitofrontal cortex and an increased 5-HI/KYNA ratio has been related to better working memory performances [172], suggesting a different role for Trp metabolites on cognitive processes.

However, only future studies precisely assessing 5-HI and KYNA changes after insulin manipulations, as well as exploring other components of the serotoninergic system, will shed light on the insulin regulation of Trp and 5-HT pathways.

### Limitations

Our study presents several limitations. First, only a limited number of studies were conducted on humans. Second, the variability in animal models and study types might account for high heterogeneity, though partially reduced by subgroup analyses. Thus, attention needs to be paid to extending results to humans. Lastly, whilst informative and comprehensive, the present meta-analysis could not systemically allow for in-depth stratification of the results. Specifically, subgroup and meta-regression analyses could not selectively compare different brain regions and other meaningful variables such as glucose and insulin plasma levels across the additional study and animal types due to the lack of correspondingly primary studies, resulting in residual confounding factors. From this perspective, the present results need to be interpreted with caution.

### Conclusions and future direction: implications for treatment-resistant schizophrenia

Despite the limitations, the present meta-analysis provided strong evidence that systemic and brain-selective insulin action manipulations might produce significant dysregulation in multiple neurotransmitter pathways, including the glutamatergic, dopaminergic, serotonergic, and GABAergic ones. The most striking observation was the effects of insulin resistance on glutamatergic and dopaminergic neurotransmission, which reproduced a few cores of abnormalities reported in animal modeling of psychotic disorders (i.e., reduction in NMDAR subunits NR2A and NR2B, PSD-95, altered DAT activity) [173, 174] and in post-mortem studies conducted on schizophrenia patients [133, 136, 175]. Based on preclinical studies, these findings could be of relevant translational value in light of growing evidence pointing to metabolic disturbances as primitive features of schizophrenia, and not only secondarily due to antipsychotic medications [176]. Considering the multifactorial etiology of schizophrenia, dysfunction in insulin action could be regarded as one of the multiple factors potentially contributing to the pathophysiology of the disorder and possibly associated with the heterogeneity of the clinical manifestations and related neuroimaging findings. Consistently, in first-episode psychosis and antipsychotic naïve patients, a potential association of insulin resistance with diminished response to antipsychotic treatment can be identified [8]. Further, abnormal neuroimaging findings have been detected in diabetic and pre-diabetic patients without psychiatric illness [177–179]. Thus, greater attention should be paid to different patterns of brain functional and structural connectivity in psychotic patients detected in the absence or presence of metabolic abnormalities, with further implications for treatment responsiveness and resistance. In addition, studies exploring alterations in neurotransmitter pathways in patients with schizophrenia should also control for metabolic variables to discern effects due to insulin action disorders from those related to schizophrenia per se.

As extensively discussed above, insulin dysregulation may mirror some of the neurobiological features detected in translational and clinical models of TRS and thus undermine the efficacy of antipsychotics. Specifically, it has been proposed that antipsychotics may enhance the insulin/Akt/FOXO signaling [180], which is less likely to be activated in a condition of insulin resistance. This pathway may, at least in part, be involved in the antipsychotic mechanism of action and its impairment may, in turn, reflect on treatment efficacy. In agreement with this hypothesis, Sevak et al. reported a markedly reduced sensitivity to behavioral effects of D2R antagonists in an animal model of streptozotocin-induced diabetes [20] and a decrease in raclopride-induced catalepsy after both streptozotocin administration and food deprivation, restored by insulin exposure and free access to food respectively [21, 22].

In addition, clozapine, the most effective antipsychotic approved for TRS therapy [181–183], has shown to enhance Akt activation and the following inhibitory phosphorylation of glycogen synthase kinase-3β, accountable for an increase in cyclic AMP response element binding protein DNA binding and beneficial effects on synaptic plasticity and metaplasticity [184–187]. Noteworthy, it has been proven that clozapine-induced Akt activation requires integrity of the insulin signaling and seems to be attenuated when insulin secretion is reduced by octreotide exposure [19].

The Akt pathway is gradually gaining interest as a putative target for the development of senolytic agents, which selectively clear senescent cells with detrimental effects on tissue function and integrity [188]. In light of the Geroscience Hypothesis, interventions that regulate cellular senescence may attenuate neuroinflammation, tissue remodeling, and mitochondrial dysfunction and exert, at once, beneficial effects on the course of multiple chronic diseases, including schizophrenia and diabetes [188].

The onset of insulin resistance condition during the neurodevelopmental process may be associated with stable neurotransmitter and molecular alterations in the central nervous system, as proved by the high prevalence of neurodevelopmental disorders in childhood-onset diabetes [189]. On the other hand, late-onset glucose metabolism dysregulations, typical of T2D, may not be accompanied by stable brain abnormalities or induce only slight modifications because the neurodevelopment process is already completed. However, in patients with schizophrenia, alterations in the insulin pathway may be noteworthy by affecting already dysfunctional neurotransmitter systems and impacting clinical outcomes [8]. In this regard, the administration of antipsychotics could have different, time-dependent consequences. Short-term antipsychotic therapy may result in a condition of increased blood glucose and hyperinsulinemia [190], possibly leading to increased brain levels of insulin as well as overstimulation of the brain insulin receptor downstream pathway. On the other hand, antipsychotic chronic treatment may induce an inflammatory state, peripheral insulin resistance, and metabolic syndrome [191], with harmful consequences on the central nervous system possibly triggering a state of brain insulin resistance. Therefore, not only could treatment and prevention of insulin resistance improve the quality of life and clinical outcomes of patients with schizophrenia [192], but also influence psychopathological manifestations.

The high concordance between brain and systemic insulin-resistant and hypoinsulinemic models, exhibiting often changes in molecular outcomes towards the same direction, strongly supports the involvement of the insulin receptor and the downstream pathway and no other epiphenomena of insulin resistance as responsible for the above-discussed disruptions. Therefore, novel agents specifically targeting the brain insulin pathway may represent a futuristic strategy to counteract psychotic features even in TRS patients, especially in addition to already available drugs such as D2R-blockers and clozapine, upon careful selection of specific patient subsets.

### Supplementary information

Supplementary Information

## Data Availability

Data are fully available upon request.
