# Supplementary material for: Insulin effects on core neurotransmitter pathways involved in schizophrenia neurobiology: a meta-analysis of preclinical studies. Implications for the treatment
Source: Mol Psychiatry. 2023 Apr 21;28(7):2811–25. doi: 10.1038/s41380-023-02065-4 (PMC10615753; doi:10.1038/s41380-023-02065-4)
Supplement: Supplementary file 1 — Supplementary Information [file 41380_2023_2065_MOESM1_ESM.docx]

**SUPPLEMENTARY MATERIALS**

The following search string was adopted for **Pubmed/MEDLINE:** *("insulin"[Title/Abstract] OR "hyperinsulinemia"[Title/Abstract] OR "hypoinsulinemia"[Title/Abstract] OR "hyperinsulinism"[Title/Abstract] OR "insulin"[MeSH Terms] OR "insulin resistance"[MeSH Terms] OR "glut"[Title/Abstract]) AND ("dopamine"[Title/Abstract] OR "dat"[Title/Abstract] OR "glutamate"[Title/Abstract] OR "nmda"[Title/Abstract] OR "ampa"[Title/Abstract] OR "kainate"[Title/Abstract] OR "gaba"[Title/Abstract] OR "eaat"[Title/Abstract] OR "dopamine"[MeSH Terms] OR "glutamic acid"[MeSH Terms] OR "gamma-Aminobutyric Acid"[MeSH Terms] OR "serotonin"[Title/Abstract] OR "5ht"[Title/Abstract] OR "serotonin"[MeSH Terms] OR "tyrosine hydroxylase"[Title/Abstract] OR "dopa decarboxylase"[Title/Abstract] OR "tryptophan hydroxylase"[Title/Abstract] OR "gaba transaminase"[Title/Abstract] OR "glutamic acid decarboxylase"[Title/Abstract] OR "glutaminase"[Title/Abstract] OR "glycine"[Title/Abstract] OR "d aspartate"[Title/Abstract] OR "d serine"[Title/Abstract]) AND ("brain"[Title/Abstract] OR "central nervous system"[Title/Abstract] OR "cns"[Title/Abstract] OR "cortex"[Title/Abstract] OR "striatum"[Title/Abstract] OR "substantia nigra"[Title/Abstract] OR "vta"[Title/Abstract] OR "ventral tegmental area"[Title/Abstract] OR "accumbens"[Title/Abstract] OR "mesolimbic"[Title/Abstract] OR "cortical"[Title/Abstract] OR "hypothalamus"[Title/Abstract] OR "pyramidal"[Title/Abstract] OR "neuron"[Title/Abstract] OR "interneuron"[Title/Abstract] OR "raphe"[Title/Abstract] OR "synapse"[Title/Abstract] OR "dendritic spine"[Title/Abstract] OR "post synaptic density"[Title/Abstract] OR "presynaptic"[Title/Abstract])*.

The following search string was used for **EMBASE:** Search#1: dopamine:ab,ti OR 'dopamine transporter':ab,ti OR 'dopaminergic system':ab,ti OR 'dopamine receptor':ab,ti OR 'glutamic acid':ab,ti OR 'n methyl dextro aspartic acid receptor':ab,ti OR 'ampa receptor':ab,ti OR 'kainic acid receptor':ab,ti OR 'glutamatergic synapse':ab,ti OR '4 aminobutyric acid':ab,ti OR '4 aminobutyric acid receptor':ab,ti OR 'excitatory amino acid transporter':ab,ti OR serotonin:ab,ti OR 'serotonin receptor':ab,ti OR glycine:ab,ti OR 'dextro aspartic acid':ab,ti OR 'dextro serine':ab,ti OR 'tyrosine 3 monooxygenase':ab,ti OR 'aromatic levo amino acid decarboxylase':ab,ti OR 'tryptophan hydroxylase':ab,ti OR '4 aminobutyrate aminotransferase':ab,ti OR 'glutamate decarboxylase':ab,ti OR glutaminase:ab,ti

Search #2: insulin:ab,ti OR hyperinsulinism:ab,ti OR hyperinsulinemia:ab,ti OR hypoinsulinemia:ab,ti OR 'insulin resistance':ab,ti OR 'glucose transporter':ab,ti

Search #3: 'brain'/exp OR brain OR 'central nervous system':ab,ti OR 'brain cortex':ab,ti OR 'corpus striatum':ab,ti OR 'substantia nigra':ab,ti OR 'ventral tegmentum':ab,ti OR 'nucleus accumbens':ab,ti OR 'limbic system':ab,ti OR hypothalamus:ab,ti OR 'pyramidal nerve cell':ab,ti OR 'nerve cell':ab,ti OR interneuron:ab,ti OR 'raphe nucleus':ab,ti OR synapse:ab,ti OR 'presynaptic nerve':ab,ti OR 'dendritic spine':ab,ti

Search #4: #1 AND #2 AND #3

The following search string was employed for **Scopus:** Search#1: TITLE-ABS ( "dopamine" ) OR TITLE-ABS ( "dat" ) OR TITLE-ABS ( "Dopamine transporter" ) OR TITLE-ABS ( "glutamate" ) OR TITLE-ABS ( "NMDA" ) OR TITLE-ABS ( "AMPA" ) OR TITLE-ABS ( "kainate" ) OR TITLE-ABS ( "GABA" ) OR TITLE-ABS ( "Serotonin" ) OR TITLE-ABS ( "5-HT" ) OR TITLE-ABS ( "tyrosine hydroxylase" ) OR TITLE-ABS ( "tryptophan hydroxylase" ) OR TITLE-ABS ( "gaba transaminase" ) OR TITLE-ABS ( "glutamic acid decarboxylase" ) OR TITLE-ABS ( "glutaminase" ) OR TITLE-ABS ( "glycine" ) OR TITLE-ABS ( "d-aspartate" ) OR TITLE-ABS ( "d-serine" ) OR TITLE-ABS ( "dopa decarboxylase" )

Search #2: TITLE-ABS ( "insulin" ) OR TITLE-ABS ( "hyperinsulinemia" ) OR TITLE-ABS ( "hyperinsulinism" ) OR TITLE-ABS ( "hypoinsulinemia" ) OR TITLE-ABS ( "insuline resistance" ) OR TITLE-ABS ( "GLUT" )

Search #3: TITLE-ABS ( "brain" ) OR TITLE-ABS ( "CNS" ) OR TITLE-ABS ( "cortex" ) OR TITLE-ABS ( "striatum" ) OR TITLE-ABS ( "substantia nigra" ) OR TITLE-ABS ( "VTA" ) OR TITLE-ABS ( "mesolimbic" ) OR TITLE-ABS ( "accumbens" ) OR TITLE-ABS ( "cortical" ) OR TITLE-ABS ( "hypothalamus" ) OR TITLE-ABS ( "raphe" ) OR TITLE-ABS ( "pyramidal" ) OR TITLE-ABS ( "neuron" ) OR TITLE-ABS ( "interneuron" ) OR TITLE-ABS ( "synapse" ) OR TITLE-ABS ( "dendritic spine" ) OR TITLE-ABS ( "post-synaptic density" ) OR TITLE-ABS ( "presynaptic" )

Search #4: #1 AND #2 AND #3

***3.1. Hyperinsulinemic model***

Overall, 97 interventional studies were found to explore the modification in the NT pathways involved in schizophrenia in animals with or without a hyperinsulinemic state. One study was found to investigate the effect of centrally administered insulin on human striatal dopaminergic activity. The studies were conducted in mice, rats, cats, dogs, fish, chickens, piglets, Lymnaea stagnalis, and Caiman latirostris. The most common model used to create a hyperinsulinemic condition was insulin injection or incubation; tolbutamide administration and acute exposure to a high-fat diet were used too. Brain area, model, study and animal types, and meta-regression for the time from insulin injection and blood glucose were helpful to explain the high levels of heterogeneity observed.

*3.1.1. Dopaminergic system*

A total of 31 animal interventional studies analyzed the alterations in the dopaminergic system under hyperinsulinemic conditions (insulin or tolbutamide treatment, or acute exposition to high fat diet) in mice, rats, cats, fish, and chicken. Twenty-four studies were conducted *ex vivo* ^1-24^, 4 *in vivo* ^25-28^, and 3 *in vitro* ^29-31^. One randomized, placebo-controlled, blinded, crossover trial was conducted on humans to explore the effect of centrally administered insulin on striatal dopaminergic activity ^32^. Hyperinsulinemic animals showed a significant increase in 3-methoxytyramine (3-MT) (Hedges’g=1.11; 95%C.I.=0.45, 1.76; p=0.001; I^2^=46.96%; based on 1 *ex vivo* study providing 6 comparisons) and 3,4-dihydroxyphenylacetic acid (DOPAC) concentrations (Hedges’g=0.51; 95%C.I.=0.21, 0.82; p=0.001; I^2^=40.67%; based on 21 comparisons from 6 studies), in dihydroxyphenylalanine (DOPA) accumulation (Hedges’g=1.94; 95%C.I.=0.30, 3.58; p=0.021; I^2^=84.90%; based on 2 *ex vivo* studies fetching 11 comparisons), in dopamine transporter (DAT) V_max_ (Hedges’g=0.33; 95%C.I.=0.12, 0.54; p=0.002; I^2^=28.02%; based on 1 *in vitro* study providing 6 comparisons), in dopamine D2 receptor (D2R) mRNA (Hedges’g=1.79; 95%C.I.=0.37, 3.20; p=0.013; I^2^=87.26%; based on 7 comparisons from 3 studies), and in tyrosine hydroxylase (TH) mRNA (Hedges’g=0.34; 95%C.I.=0.04, 0.64; p=0.027; I^2^=16.18%; based on 3 studies fetching 14 comparisons). Animals receiving insulin or tolbutamide also showed a lower monoamine oxidase (MAO) activity (Hedges’g=-0.86; 95%C.I.=-1.28, -0.44; p<0.001; I^2^=36.32%; based on 1 *ex vivo* study fetching 9 comparisons) and dopamine (DA) concentration (Hedges’g=-0.39; 95%C.I.=-0.77, -0.01; p=0.043; I^2^=82.47%; based on 68 comparisons from 15 studies).

Subgroup analysis among studies exploring DA concentration showed a significant reduction in *ex vivo* studies and a significant increase in *in vivo* studies; likewise, a significant drop in DA concentration was detected in the brainstem, cortex, hippocampus, olfactory lobe, and nucleus accumbens of the hyperinsulinemic group. For most animal and study types, hyperinsulinemic models and brain areas were effective in reducing heterogeneity. Publication bias was detected for DOPAC (*Egger test: beta*=3.44, p=0.044; Hedges’g=0.35; 95%C.I.=0.01, 0.69) and DA concentrations (*Egger test: beta*=-2.84, p=0.001; Hedges’g=-0.17; 95%C.I.=-0.58, 0.24).

*3.1.2. Glutamatergic system*

A total of 44 animal interventional studies explored the alterations in the glutamatergic system under hyperinsulinemic conditions (insulin injection or incubation, or acute exposition to a high-fat diet) in rats, mice, dogs, piglets, and Caiman latirostris. Eleven studies were run *in vitro* ^33-43^, 4 *in vivo* ^44-47^, and 27 *ex vivo* ^15, 48-73^. Two studies ^74, 75^ included both an *in vivo* and an *ex vivo* part. Hyperinsulinemic animals significantly differed from their controls in various comparisons, presenting higher mRNA levels of the kainate receptor subunits GLUR5 (Hedges’g=1.57; 95%C.I.=0.90, 2.23; p<0.001; I^2^=0%; based on 1 study providing 3 comparisons) and KA2 (Hedges’g=1.97; 95%C.I.=1.26, 2.67; p<0.001; I^2^=0%; based on 1 study fetching 3 comparisons), and a higher glutamine synthase activity rate (Hedges’g=1.61; 95%C.I.= 0.41, 2.81; p<0.001; I^2^=79.16%; based on 6 comparisons from 3 studies). Animals receiving insulin also showed lower glutamate (Hedges’g=-0.49; 95%C.I.=-0.83, -0.16; p=0.004; I^2^=82.286%; based on 71 comparisons from 17 animal interventional studies) and glutamine concentrations (Hedges’g=-1.17; 95%C.I.=-1.64, -0.70; p<0.001; I^2^=84.27%; based on 8 studies fetching 41 comparisons). Subgroup analysis among studies exploring glutamate concentration showed a significant reduction in *ex vivo* studies and a significant increase in *in vitro* and *in vivo* studies; glutamine concentration was not affected by the study type. For most, animal and study types, and brain area were effective in reducing heterogeneity. A meta-regression conducted in Sprague-Dawley rats showed a significantly further decrease in glutamine concentrations with the increase of the blood glucose ratio between controls and cases. Publication bias was detected for glutamate (*Egger test: beta*=-2.60, p=0.008; Hedges’g=-0.45; 95%C.I.=-0.80, -0.09) and glutamine concentrations (*Egger test: beta*=-8.57, p<0.001; Hedges’g=-0.43; 95%C.I.=-1.02, 0.15).

*3.1.3. Serotoninergic system*

A total of 23 animal interventional studies appraised the alterations in the serotoninergic system under hyperinsulinemic conditions (insulin or tolbutamide treatment) in rats, chickens, fish, and Lymnaea stagnalis. Twenty-one studies were conducted *ex vivo* ^1, 2, 5, 8, 10, 13, 19, 20, 22, 24, 76-86^, 1 *in vitro* ^87^ and 1 *in vivo* ^27^. Animals showed a significant increase in 5-hydroxy indole acetic acid (5-HIAA) concentration (Hedges’g=0.40; 95%C.I.=0.12, 0.68; p=0.005; I^2^=75.62%; based on 64 comparisons from 17 studies) and in tryptophan (Trp) accumulation (Hedges’g=2.22; 95%C.I.=1.39, 3.05; p<0.001; I^2^=41.79%; based on 1 *ex vivo* study fetching 5 comparisons) after insulin or tolbutamide administration. Animal type and brain area successfully explained the high value of heterogeneity for 5-HIAA concentration. Publication bias was detected for 5-HIAA concentration (*Egger test: beta*=1.83, p=0.04; Hedges’g=0.25; 95%C.I.=-0.05, 0.55).

*3.1.4. GABAergic system*

A total of 22 animal interventional studies investigated the alterations in the GABAergic system under hyperinsulinemic conditions (insulin injection or incubation) in rats, mice, and dogs. Fourteen studies were conducted *ex vivo* ^48, 54-57, 62, 64, 65, 68-70, 88-90^, 3 *in vivo* ^47, 91, 92^, and 4 *in vitro* ^35, 93-95^. One study ^96^ comprised both an *in vivo* and an *ex vivo* part. Animals treated with insulin significantly differed from their controls in several outcomes, showing lower glutamic acid decarboxylase (GAD) mRNA levels (Hedges’g=-6.35; 95%C.I.=-10.05, -2.64; p=0.001; I^2^=83.59%; based on 3 comparisons from 3 *ex vivo* studies), GABA-A_α1_ mRNA expression (Hedges’g=-8.91; 95%C.I.=-14.69, -3.13; p=0.003; I^2^=90.31%; based on 3 comparisons from 3 *ex vivo* studies), GABA Receptor B_max_ (Hedges’g=-5.87; 95%C.I.=-8.93, -2.82; p<0.001; I^2^=78.66%; based on 3 *ex vivo* studies fetching 3 comparisons), GABA turnover (Hedges’g=-1.40; 95%C.I.=-2.02, -0.77; p<0.001; I^2^=0%; based on 1 study fetching 3 comparisons), and GABA concentration (Hedges’g=-0.58; 95%C.I.=-0.85, -0.32; p<0.001; I^2^=75.09%; based on 70 comparisons from 14 studies). Animals receiving insulin also displayed higher GABA-A receptor β_2_/β_3_ subunits levels in the hippocampus (Hedges’g=0.48; 95%C.I.=0.27, 0.70; p<0.001; I^2^=17.51%; based on 1 *in vitro* study providing 5 comparisons) and tonic currents amplitude (mediated by GABA-A) in CA1 pyramidal neurons (Hedges’g=0.74; 95%C.I.=0.22, 1.26; p=0.006; I^2^=0%; based on 1 *in vitro* study fetching 3 comparisons). Subgroup analysis showed a significant reduction in GABA concentration after insulin administration only in *ex vivo* studies. Study and animal type, and brain area successfully explained the high value of heterogeneity for all the significant outcomes considered. Publication bias was detected for GABA concentration (*Egger test: beta*=-7.61, p<0.001; Hedges’g=-0.49; 95%C.I.=-0.78, -0.20).

***3.2 Hypoinsulinemic model***

Overall, 80 animal interventional studies explored NT pathways modifications induced by insulin deficiency due to streptozotocin or alloxan injection, genetic type 1 diabetes (T1D), low-protein diets, and caloric restriction. The studies were conducted on mice, rats, and pigs.

*3.2.1 Dopaminergic system*

A total of 40 animal interventional studies investigated how insulin deficit due to streptozotocin or alloxan injection, genetic spontaneous hypoinsulinemia, and caloric restriction, affects brain dopaminergic system in mice, rats, and pigs. Thirty-six studies were run *ex vivo* ^6, 11, 22, 97-129^ and 3 *in vivo* ^130-132^. One study comprised both an *in vivo* and an *ex vivo* part ^133^. Hypoinsulinemic animals showed a significant reduction in [^3^H]DA uptake (Hedges’g=-1.371; 95%C.I.=-1.710, -1.032; p=0.000; I^2^=0%; based on 1 study providing 4 comparisons) and in TH+ neurons (Hedges’g=-0.787; 95%C.I.=-1.528, -0.045; p=0.038; I^2^=34.127%; based on 1 study providing 3 comparisons), and a significant increase in DR2 mRNA expression (Hedges’g=3.259; 95%C.I.=0.497, 6.020; p=0.021; I^2^=90.61%; based on 3 study providing 3 comparisons), in TH activity (Hedges’g=1.060; 95%C.I.=0.681, 1.440; p=0.000; I^2^=0%; based on 1 study providing 8 comparisons), and in MAO activity (Hedges’g=0.545; 95%C.I.=0.158, 0.904; p=0.003; I^2^=0%; based on 1 study providing 8 comparisons). Subgroup analysis among studies exploring DR2 mRNA expression showed a significant increase in the hippocampus and striatum.

*3.2.2. Glutamatergic system*

A total of 24 animal interventional studies investigated the modification in the glutamatergic system due to insulin deficiency in mice and rats. The hypoinsulinemic condition was established through streptozotocin or alloxan injection or genetic T1D. Twenty-two studies were run *ex vivo* ^58, 63, 68-70, 102, 134-149^, and 2 *in vivo* ^130, 150^. Animals in the hypoinsulinemic group showed a significant increase in ^3^[H]-AMPA-R binding (Hedges’g=0.89; 95%C.I.=0.13, 1.65; p=0.022; I^2^=73.11%; based on 1 study providing 9 comparisons), NMDA-R B_max_ (Hedges’g=4.99; 95%C.I.=2.24, 7.74; p<0.001; I^2^=79%; based on 3 studies fetching 3 comparisons), and glutamate dehydrogenase (GDH) V_max_ (Hedges’g=5.93; 95%C.I.=2.59, 9.27; p<0.001; I^2^=88.04%; based on 2 studies fetching 5 comparisons). Streptozotocin-treated animals displayed a significant reduction in the hippocampal protein levels of glutamate ionotropic receptor NMDA type subunit 2B (NR2B) (Hedges’g=-2.03, 95%C.I.=-3.67, -0.39; p=0.015; I^2^=79.79%; based on 2 studies fetching 4 comparisons). The brain area, model, and animal type explained the high heterogeneity values in the significant outcomes.

*3.2.3. Serotoninergic system*

A total of 35 animal interventional studies explored the effect of hypoinsulinemia on serotoninergic neurotransmission. Streptozotocin, alloxan, caloric restriction, fasting, genetic spontaneous hypoinsulinemia and low-protein diets were adopted as models of insulin secretion decreasing conditions in mice, rats, and pigs. Thirty-two studies were run *ex vivo* ^22, 98-100, 102-109, 118, 120, 122-124, 126, 127, 151-163^ and 2 *in vivo* ^131, 164^. One study ^132^ included both an *in vivo* and *ex vivo* part. Hypoinsulinemic animals significantly diverged from healthy controls in several outcomes, presenting lower levels of 5-HIAA (Hedges’g=-0.37; 95%C.I.=-0.59, -0.16; p=0.001; I^2^=73.97%; based on 71 comparisons from 21 studies) and Trp concentration (Hedges’g=-1.35; 95%C.I.=-1.75, -0.96; p<0.001; I^2^=63.92%; based on 7 studies fetching 17 comparisons), lower Trp hydroxylase activity rate (Hedges’g=-3.93; 95%C.I.=-5.83, -2.03; p<0.001; I^2^=82.56%; based on 2 studies providing 5 comparisons) and Trp-5-hydroxylase V_max_ (Hedges’g=-45.64; 95%C.I.=-57.74, -33.53; p<0.001; I^2^=27.62%; based on 1 study providing 4 comparisons); only Trp-5-hydroxylase K_m_ resulted increase (Hedges’g=8.86; 95%C.I.=5.55, 12.17; p<0.001; I^2^=60.27%; based on 1 study providing 4 comparisons) in the hypoinsulinemic group. Brain area, animal and model type, and meta-regression conducted for the time from insulin alteration, blood glucose, and blood insulin successfully explained the high values of heterogeneity in most cases. The publication bias was detected for 5-HIAA concentration (*Egger test: beta*=-1.72, p=0.006; Hedges’g=-0.12; 95%C.I.=-0.37, 0.12) and Trp concentration (*Egger test: beta*=-6.77, p<0.001; Hedges’g=-0.90; 95%C.I.=-1.33, -0.46).

*3.2.4. GABAergic system*

A total of 12 animal interventional studies explored how a decrease in insulin secretion due to streptozotocin injection could affect the brain GABAergic pathway in mice and rats. Nine studies were run *ex vivo* ^68-70, 138, 143, 147, 165-167^ and 2 *in vivo* ^130, 168^. One study ^132^ was characterized by both an *ex vivo* and *in vivo* section. Animals treated with streptozotocin significantly diverged from their healthy controls, presenting lower GAD mRNA levels (Hedges’g=-4.23, 95%C.I.=-7.53, -1.02; p=0.01; I^2^=89.67%; based on 3 studies providing 3 comparisons) and GABA-R B_max_ (Hedges’g=-2.61, 95%C.I.=-4.70, -0.52; p=0.014; I^2^=84.69%; based on 3 studies fetching 3 comparisons), and displaying a higher GABA concentration (Hedges’g=1.37, 95%C.I.=0.45, 2.29; p=0.004; I^2^=83.60%; based on 12 comparisons from 6 studies) and GABA tonic current density (Hedges’g=1.11, 95%C.I.=0.15, 2.08; p=0.023; I^2^=69.73%; based on 1 study fetching 3 comparisons). Brain area and study type successfully explained the high values of heterogeneity. Publication bias was detected for GABA concentration (*Egger test: beta*=7.27, p=0.001; Hedges’g=0.76; 95%C.I.=-0.06, 1.58).

***3.3. Insulin-resistant model***

Overall, 34 animal interventional studies, 5 human cross-sectional studies ^169-173^, and 1 human randomized clinical trial ^174^ were found to investigate the impact of insulin resistance on the NT pathways involved in schizophrenia. In human studies, subjects affected by T2D, metabolic syndrome, and obesity were compared to healthy controls. Several models were used to reproduce an insulin-resistant condition in mice and rats, including long-term high-fat or high-calorie diet, Zucker fatty rats, Otsuka Long-Evans Tokushima Fatty (OLETF) rats, spontaneous T2D, monosodium glutamate, and a polygenic model of type 2 diabetes (NONcNZO10/LtJ mice).

*3.3.1. Dopaminergic system*

A total of 16 animal interventional studies ^10, 175-189^, 2 human cross-sectional studies ^169, 170^, and 1 human randomized clinical trial ^174^ explored the modifications in the dopaminergic system in spontaneous insulin-resistant subjects, in rats and mice after a long-term high-fat diet, in Zucker fatty rats and OLETF rats. Five studies were run *in vivo* ^169, 170, 174-176^ and 10 *ex vivo* ^10, 178, 180, 181, 183-188^. Three studies ^177, 179, 182^ comprised an *in vivo* and *ex vivo* part. Animals in the brain insulin-resistant group presented a significant impairment in DAT B_max_ (Hedges’g=-1.13, 95%C.I.=-1.90, -0.36; p=0.004; I^2^=0%; based on 1 *ex vivo* study fetching 3 comparisons), V_max_ (Hedges’g=-1.48, 95%C.I.=-2.18, -0.78; p<0.001; I^2^=11.47%; based on 1 *ex vivo* study fetching 3 comparisons), K_m_ (Hedges’g=-1.07, 95%C.I.=-1.85, -0.30; p=0.007; I^2^=19.27%; based on 1 *ex vivo* study fetching 3 comparisons), and DAT cell surface expression (Hedges’g=-1.14, 95%C.I.=-1.91, -0.37; p=0.004; I^2^=24.76%; based on 2 *ex vivo* studies providing 3 comparisons). Insulin-resistant animals also displayed a significant decrease in striatal DA clearance (Hedges’g=-1.48, 95%C.I.=-2.18, -0.78; p<0.001; I^2^=11.47%; based on 1 *in vivo* study fetching 5 comparisons) and a significant increase in DA extraction fraction (Hedges’g=0.97, 95%C.I.=0.15, 1.79; p<0.001; I^2^=11.47%; based on 1 *in vivo* study providing 3 comparisons). For most, model and animal types, and brain area were effective to explain the highest values of heterogeneity.

*3.3.2. Glutamatergic system*

A total of 14 animal interventional studies ^142, 190-202^ and 3 human cross-sectional studies ^171-173^ explored the effect of high-fat and high-calorie diet, monosodium glutamate and, spontaneous T2D on the glutamatergic neurotransmission in mice and rats. Five studies were run in vivo ^171-173, 190, 192^ and 11 ex vivo ^142, 191, 193-195, 197-202^. One study ^196^ comprised both an *in vivo* and *ex vivo* part. The insulin-resistant group displayed a significative drop in the hippocampal levels of several proteins involved in NMDA function, including glutamate ionotropic receptor NMDA type subunit 2A (NR2A) (Hedges’g=-0.95, 95%C.I.=-1.50, -0.39; p=0.001; I^2^=47.46%; based on 3 studies providing 8 comparisons), glutamate ionotropic receptor NMDA type subunit 2B (NR2B) (Hedges’g=-0.69, 95%C.I.=-1.35, -0.02; p=0.043; I^2^=62.09%; based on 3 studies fetching 8 comparisons), and postsynaptic density protein 95 (PSD-95) (Hedges’g=-0.91, 95%C.I.=-1.51, -0.32; p=0.003; I^2^=77.81%; based on 14 comparisons from 4 studies). Even though data were insufficient to provide a meta-analysis, glutamate ionotropic receptor NMDA type subunit 1 (NR1) ^194^ also decreased under insulin-resistant conditions. The protein levels of vesicular glutamate transporter 1 (VGLUT1) appeared to be significantly reduced (Hedges’g=-1.07, 95%C.I.=-1.55, -0.60; p<0.001; I^2^=59.48%; based on 12 comparisons from 4 studies) in insulin-resistant animals. The high heterogeneity values were explained in most cases by subgroup analysis conducted for brain area and animal and model type. A sensitivity analysis was conducted for NR2B protein levels, excluding an outlier score ^193^, and the result appeared still significant (Hedges’g=-0.50, 95%C.I.=-0.89, -0.11; p=0.013; I^2^=0%). Regarding PSD-95 and VGLUT1 protein levels following an insulin-resistant state, subgroup analysis of the brain area showed a significant drop, specifically in the hippocampus. Publication bias was detected for PSD-95 protein levels (*Egger test: beta*=-7.52, p=0.001; Hedges’g=-0.76; 95%C.I.=-1.38, -0.15), VGLUT1 protein levels (*Egger test: beta*=-5.14, p=0.01; Hedges’g=-0.73; 95%C.I.=-1.21, -0.25).

*3.3.3. Serotoninergic system*

A total of 6 animal intervention studies ^10, 181, 188, 203-205^ and 1 human cross-sectional study ^170^ investigated the modification of the serotoninergic system in mice and rats after a long-term high-fat diet, in Zucker fatty rats, and in spontaneous T2D. Two studies were run *in vivo* ^170, 204^, and 5 *ex vivo* ^10, 181, 188, 203, 205^. Meta-analysis revealed no significant alteration in the insulin-resistant group. However, a meta-regression conducted in Zucker rats for 5-HIAA concentrations showed that the age of animals significantly explained heterogeneity across studies, with an association between increasing age and lower effect sizes (Hedges’s g).

*3.3.4. GABAergic system*

A total of 5 interventional studies ^191, 196, 201, 202, 206^ in mice and rats, and 1 human cross-sectional study ^173^ were found to analyze the effect of insulin resistance on GABA neurotransmission in subjects with spontaneous T2D, after a long-term high-fat diet, or in NONcNZO10/LtJ mice. One study was performed *in vivo* ^173^ and 4 *ex vivo* ^191, 201, 202, 206^. One study ^196^ included both an *in vivo* and *ex vivo* part. Insulin-resistant animals showed a significant reduction in vesicular GABA transporter (VGAT) protein levels (Hedges’g=-0.75, 95%C.I.=-1.10, -0.39; p<0.001; I^2^=0%; based on 2 studies fetching 7 comparisons) and a significant increase in GABA concentration (Hedges’g=0.34, 95%C.I.=0.02, 0.66; p=0.04; I^2^=0%; based on 2 studies providing 8 comparisons) and glutamic acid decarboxylase 67 (GAD-67) protein levels (Hedges’g=0.77, 95%C.I.=0.22, 1.32; p=0.006; I^2^=0%; based on 1 study fetching 4 comparisons).

***3.4. Brain insulin-resistant model***

Overall, 9 animal interventional studies ^207-215^ were found to explore the effect of brain insulin-resistance on the NT pathways involved in the pathogenesis of schizophrenia. Eight studies were run *ex vivo* and 1 *in vivo.* Several models were adopted to create a condition of brain insulin resistance i.e., intracerebroventricular administration of streptozotocin or alloxan in rats, inactivation of the insulin receptor gene in astrocytes (GIRKO mice), brain/neuron-specific insulin receptor knockout (NIRKO mice), mice with central nervous system restricted deletion of insulin receptor substrate 2 (NesCreIrs2KO), and hippocampal-specific insulin resistance due to a lentiviral vector expressing an IR antisense sequence (LV-IRAS). Brain area and animal and model types successfully explained the high value of heterogeneity in all the cases. Publication bias and meta-regression analysis were not performed since no outcome with at least ten comparisons were available.

*3.4.1. Dopaminergic system*

A total of 3 animal interventional studies ^207-209^ investigated the modifications in the dopaminergic system in a brain insulin-resistant model (intracerebroventricular alloxan injection in Wistar rats, and GIRKO and NIRKO mice). All the studies were conducted *ex vivo*. Brain insulin-resistant animals showed a significant increase in DA levels (Hedges’g=0.87, 95%C.I.=0.05, 1.69; p=0.037; I^2^=44.29%; based on 2 studies fetching 3 comparisons). The heterogeneity was successfully explained by model type.

*3.4.2. Glutamatergic system*

A total of 6 animal interventional studies ^210-215^ explored the alteration in the glutamatergic system due to brain insulin resistance (LV-IRAS, NesCreIrs2KO mice and intracerebroventricular administration of streptozotocin) in mice and rats. All the studies were run *ex vivo*. Animals in the brain insulin-resistant group presented a significant drop in the hippocampal protein levels of NR2A (Hedges’g=-2.11; 95%C.I.=-4.14, -0.09; p=0.041; I^2^=88.00%; based on 3 studies providing 3 comparisons). Although not significant, hippocampal protein levels of NR2B (Hedges’g=-0.69; 95%C.I.=-1.98, 0.60; p=0.293; I^2^=78.02%) appeared to be reduced too. Even though data were not sufficient to provide a quantitative analysis, brain insulin-resistant animals showed a decrease in the hippocampal levels of NR1 ^214, 215^. Animal and model type successfully explained the high value of heterogeneity.

*3.4.3. GABAergic system*

Two animal interventional studies ^213, 214^ explored the modifications of GABA pathway after the intracerebroventricular administration of streptozotocin in rats. One *ex vivo* ^214^ study provided the values of GABA_B1_ and GABA_B2_ in the hippocampus of Sprague-Dawley rats. Another *in vivo* ^213^ study measured GABA concentration in the hippocampus and cortex of Wistar rats through ^1^H MR Spectroscopy. The data were insufficient to perform a meta-analysis.

**Supplementary Table 1: SYRCLE quality assessment of included animal studies**

| Author(s), year | SYRCLE 1 | SYRCLE 2 | SYRCLE 3 | SYRCLE 4 | SYRCLE 5 | SYRCLE 6 | SYRCLE 7 | SYRCLE 8 | SYRCLE 9 | SYRCLE 10 | SYRCLE total |
| --- | --- | --- | --- | --- | --- | --- | --- | --- | --- | --- | --- |
| Abdul-Ghani A.-S. et al., 1989 | Unclear | Yes | Unclear | Unclear | Unclear | Unclear | Unclear | Unclear | Yes | Yes | 3 |
| Abraham P.M. et al., 2010 (a) | Unclear | Yes | Unclear | Unclear | Unclear | Unclear | Unclear | Unclear | Yes | Yes | 3 |
| Abraham P.M. et al., 2010 (b) | Yes | Yes | Unclear | Unclear | Unclear | Unclear | Unclear | No | Yes | Yes | 4 |
| Agardh C.-D. et al., 1979 | Unclear | Yes | Unclear | Unclear | Unclear | Unclear | Unclear | No | Yes | No | 2 |
| Alhamami H.N. et al., 2017 | Unclear | Yes | Unclear | Unclear | Unclear | Unclear | Unclear | Yes | Yes | Yes | 4 |
| Alshamrani A.A. et al., 2020 | Yes | Yes | Unclear | Unclear | Unclear | Unclear | Unclear | Yes | Yes | No | 4 |
| Anderzhanova E. et al., 2007 | No | No | Unclear | Unclear | Unclear | Unclear | Unclear | Yes | Yes | Yes | 3 |
| Anitha M. et al., 2012 | Yes | Yes | Unclear | Unclear | Unclear | Unclear | Unclear | Yes | Yes | Yes | 5 |
| Aounuma H. et al., 2018 | Unclear | Yes | Unclear | Unclear | Unclear | Unclear | Yes | Yes | Yes | No | 4 |
| Atienza G. et al., 1995 | Unclear | Yes | Unclear | Unclear | Unclear | Unclear | Unclear | No | Yes | Yes | 3 |
| Barry L.R. et al., 2018 | Unclear | Yes | Unclear | Unclear | Unclear | Unclear | Unclear | No | Yes | Yes | 3 |
| Berggren U. et al., 1983 | Unclear | Yes | Unclear | Unclear | Unclear | Unclear | Unclear | No | Yes | No | 2 |
| Beverly J.L. et al., 1990 | Unclear | Yes | Unclear | Unclear | Unclear | Unclear | Unclear | No | Yes | No | 2 |
| Cai W. et al., 2018 | Unclear | No | Unclear | Unclear | Unclear | Yes | Unclear | No | Yes | Yes | 2 |
| Chance W.T. et al., 1989 | Yes | Yes | Unclear | Unclear | Unclear | Unclear | Unclear | No | Yes | Yes | 4 |
| Chaouloff F., et al 1989 | Yes | Yes | Unclear | Unclear | Unclear | Unclear | Unclear | No | Yes | Yes | 4 |
| Chaves Y.C., et al 2020 | Unclear | Yes | Unclear | Unclear | Yes | Unclear | Unclear | No | Yes | Yes | 4 |
| Chu P.C., et al 1986 | Unclear | Yes | Unclear | Unclear | Unclear | Unclear | Unclear | Yes | Yes | Yes | 4 |
| Crandall E.A., et al 1980 | Unclear | Yes | Unclear | Unclear | Unclear | Unclear | Unclear | Yes | Yes | No | 3 |
| Cruz B., et al 2021 | Yes | Yes | Unclear | Unclear | Yes | Unclear | Yes | Yes | Yes | Yes | 7 |
| Cumming P., et al 2014 | Unclear | No | Unclear | Unclear | Unclear | Unclear | Unclear | Yes | Yes | Yes | 3 |
| de Leeuw van Weenen J.E., et al 2008 | Yes | Yes | Unclear | Unclear | Unclear | Unclear | Unclear | Yes | Yes | Yes | 5 |
| Di Giulio A.M., et al 1989 | Unclear | Yes | Unclear | Unclear | Unclear | Unclear | Unclear | Yes | Yes | Yes | 4 |
| Figlewicz D.P., et al 1996 | Unclear | Yes | Unclear | Unclear | Unclear | Unclear | Unclear | No | Yes | Yes | 3 |
| Figlewicz D.P., et al 1994 | Unclear | Yes | Unclear | Unclear | Unclear | Unclear | Unclear | Yes | Unclear | Yes | 3 |
| Figlewicz D.P., et al 1997 | Unclear | No | Unclear | Unclear | Unclear | Unclear | Unclear | Unclear | Yes | Yes | 2 |
| Georgy G., et al 2013 | Unclear | Yes | Unclear | Unclear | Unclear | Unclear | Unclear | Unclear | Yes | Yes | 3 |
| Gotoh M., et al 2006 | Unclear | Yes | Unclear | Unclear | Unclear | Unclear | Unclear | Yes | Yes | Yes | 4 |
| Grunstein H.S., et al 1986 | Unclear | Yes | Unclear | Unclear | Unclear | Unclear | Unclear | Yes | Yes | Yes | 4 |
| Gupta D., et al 2014 | Yes | Yes | Unclear | Unclear | Unclear | Unclear | Unclear | Yes | Yes | No | 4 |
| Gupta D., et al 1991 | Unclear | Yes | Unclear | Unclear | Unclear | Unclear | Unclear | No | Yes | Yes | 3 |
| Guzmàn D.C., et al 2019 | Unclear | Yes | Unclear | Unclear | Unclear | Unclear | Unclear | Yes | Yes | Yes | 4 |
| Guzmàn D.C., et al (a) 2019 | Unclear | Yes | Unclear | Unclear | Unclear | Unclear | Unclear | Yes | Yes | Yes | 4 |
| Hajnal A., et al 1997 | Unclear | Yes | Unclear | Unclear | Unclear | Unclear | Unclear | Yes | Yes | Yes | 4 |
| Heyes M.P., et al 1990 | Unclear | Yes | Unclear | Unclear | Unclear | Unclear | Unclear | No | Yes | Yes | 3 |
| Huang C.C., et al 2003 | Unclear | Yes | Unclear | Unclear | Unclear | Unclear | Unclear | Yes | Yes | Yes | 4 |
| Huang C.W., et al 2016 | Yes | Yes | Unclear | Unclear | Unclear | Unclear | Unclear | Yes | Yes | Yes | 5 |
| Ibrahim M.M.H., et al 2020 | Unclear | Yes | Unclear | Unclear | Unclear | Unclear | Unclear | Yes | Yes | Yes | 4 |
| Ichord R.N., et al 2001 | Unclear | Yes | Unclear | Unclear | Unclear | Unclear | Yes | Yes | Yes | Yes | 5 |
| Jaranarayanan S., et al 2013 | Unclear | Yes | Unclear | Unclear | Unclear | Unclear | Unclear | Unclear | Yes | Yes | 3 |
| Kimura H., et al 1974 | Unclear | Yes | Unclear | Unclear | Unclear | Unclear | Unclear | Yes | Yes | No | 3 |
| Kino M., et al 2004 | Unclear | Yes | Unclear | Unclear | Unclear | Unclear | Unclear | No | Yes | Yes | 3 |
| Kleinridders A., et al 2015 | Unclear | No | Unclear | Unclear | Unclear | Unclear | Unclear | No | Yes | Yes | 2 |
| Koenig J.I., et al 2005 | Unclear | Yes | Unclear | Unclear | Unclear | Unclear | Unclear | No | Yes | Yes | 3 |
| Kohsaka S., et al 1979 | Unclear | Yes | Unclear | Unclear | Unclear | Unclear | Unclear | No | Yes | Yes | 3 |
| Kolta M.G., et al 1986 | Unclear | Yes | Unclear | Unclear | Unclear | Unclear | Unclear | No | Yes | Yes | 3 |
| Labouèbe G., et al 2013 | Unclear | Yes | Unclear | Unclear | Unclear | Unclear | Unclear | Yes | Yes | Yes | 4 |
| Lewczuk B., et al 2018 | Yes | Yes | Unclear | Unclear | Unclear | Unclear | Unclear | Yes | Yes | Yes | 5 |
| Li H., et al 2012 | Yes | Yes | Unclear | Unclear | Unclear | Unclear | Unclear | Yes | Yes | Yes | 5 |
| Lin L., et al 2018 | Unclear | Yes | Unclear | Yes | Unclear | Unclear | Unclear | Yes | Yes | Yes | 5 |
| Lozovsky D., et al 1981 | Unclear | Yes | Unclear | Unclear | Unclear | Unclear | Unclear | Yes | Yes | No | 3 |
| Lozovsky D., et al 1985 | Unclear | Yes | Unclear | Unclear | Unclear | Unclear | Unclear | No | Yes | Yes | 3 |
| MacKensie M.G., et al 1978 | Unclear | Yes | Unclear | Unclear | Unclear | Unclear | Unclear | No | Yes | No | 2 |
| Mahata S.K., et al 1990 | Unclear | Yes | Unclear | Unclear | Unclear | Unclear | Unclear | Yes | Yes | Yes | 4 |
| Martìn F.J., et al 1995 | Unclear | Yes | Unclear | Unclear | Unclear | Unclear | Unclear | Yes | Yes | Yes | 4 |
| McGowan J.E., et al 1995 | Unclear | Yes | Unclear | Unclear | Unclear | Unclear | Unclear | Yes | Yes | Yes | 4 |
| Miyata S., et al 2007 | Unclear | Yes | Unclear | Unclear | Unclear | Unclear | Unclear | No | Yes | Yes | 3 |
| Montefusco O., et al 1983 | Unclear | Yes | Unclear | Unclear | Unclear | Unclear | Unclear | No | Yes | Yes | 3 |
| Morris J.K., et al 2011 | Unclear | Yes | Unclear | Unclear | Unclear | Unclear | Unclear | No | Yes | Yes | 3 |
| Motawi T., et al 2016 | Unclear | Yes | Unclear | Unclear | Unclear | Unclear | Unclear | No | Yes | No | 2 |
| O'Dell L.E., et al 2014 | Unclear | Yes | Unclear | Unclear | Unclear | Unclear | Unclear | No | Yes | Yes | 3 |
| Ohtani N., et al 1997 | Unclear | Yes | Unclear | Unclear | Unclear | Unclear | Unclear | No | Yes | Yes | 3 |
| Okuda M.H., et al 2014 | Yes | Yes | Unclear | Unclear | Unclear | Unclear | Unclear | No | Yes | Yes | 4 |
| Orosco M., et al 1990 | Unclear | No | Unclear | Unclear | Unclear | Unclear | Unclear | Yes | Yes | Yes | 3 |
| Orosco M., et al 1991 | Unclear | Yes | Unclear | Unclear | Unclear | Unclear | Unclear | Yes | Yes | Yes | 4 |
| Owens A.W., et al 2005 | Unclear | Yes | Unclear | Unclear | Unclear | Unclear | Unclear | Yes | Yes | Yes | 4 |
| Paternain L., et al 2012 | Yes | Yes | Unclear | Unclear | Unclear | Unclear | Unclear | Yes | Yes | Yes | 5 |
| Patterson T.A., et al 1998 | Unclear | Yes | Unclear | Unclear | Unclear | Unclear | Unclear | No | Yes | Yes | 3 |
| Peeyush K.T., et al 2010 | Unclear | Yes | Unclear | Unclear | Unclear | Unclear | Unclear | Yes | Yes | Yes | 4 |
| Portero-Tressera M., et al 2020 | Unclear | Yes | Unclear | Unclear | Unclear | Unclear | Unclear | Yes | Yes | Yes | 4 |
| Ramakrishnan R., et al 2005 | Unclear | Yes | Unclear | Unclear | Unclear | Unclear | Unclear | No | Yes | Yes | 3 |
| Ramakrishnan R., et al 2009 | Unclear | Yes | Unclear | Unclear | Unclear | Unclear | Unclear | No | Yes | Yes | 3 |
| Robinson R., et al 2009 | Unclear | Yes | Unclear | Unclear | Unclear | Unclear | Unclear | No | Yes | Yes | 3 |
| Routh V.H., et al 1994 | Unclear | No | Unclear | Unclear | Unclear | Unclear | Unclear | No | Yes | Yes | 2 |
| Ruibal C., et al 2002 | Unclear | Yes | Unclear | Yes | Unclear | Unclear | Unclear | Yes | Yes | Yes | 5 |
| Salkovic M., et al 1994 | Unclear | Yes | Unclear | Unclear | Unclear | Unclear | Unclear | Yes | Yes | Yes | 4 |
| Sauter A., et al 1982 | Unclear | Yes | Unclear | Unclear | Unclear | Unclear | Unclear | No | Yes | No | 2 |
| Eswar Shankar P.N., et al 2006 | Unclear | Yes | Unclear | Unclear | Unclear | Unclear | Unclear | No | Yes | Yes | 3 |
| Sharma S., et al 2018 | Unclear | Yes | Unclear | Unclear | Unclear | Unclear | Unclear | No | Yes | Yes | 3 |
| Sherin A. et al 2011 | Unclear | Yes | Unclear | Unclear | Unclear | Unclear | Unclear | No | Yes | Yes | 3 |
| Shimizu H. et al. 1989 | Unclear | Yes | Unclear | Unclear | Unclear | Unclear | Unclear | Yes | Yes | Yes | 4 |
| Silverstein FS, 1990 | Unclear | Yes | Unclear | Unclear | Unclear | Unclear | Unclear | Yes | Yes | Yes | 4 |
| Soengas J.L. 2003 | Unclear | Yes | Unclear | Unclear | Unclear | Unclear | Unclear | Yes | Yes | Yes | 4 |
| Speed N. et al 2011 | Unclear | Yes | Unclear | Unclear | Unclear | Unclear | Unclear | No | Yes | Yes | 3 |
| Steger R.W. et al. 1990 | Unclear | Yes | Unclear | Unclear | Unclear | Unclear | Unclear | No | Yes | Yes | 3 |
| Melissa A. Stouffer et al 2015 | Yes | Yes | Unclear | Unclear | Yes | Unclear | Unclear | No | Yes | Yes | 5 |
| Trulson M.E. et al 1983 (a) | Unclear | Yes | Unclear | Unclear | Unclear | Unclear | Unclear | Yes | Yes | Yes | 4 |
| Trulson M.E: et al 1983 (b) | Unclear | Yes | Unclear | Unclear | Unclear | Unclear | Unclear | Yes | Yes | Yes | 4 |
| R. J. Vaidya et al 2017 | Yes | Yes | Unclear | Unclear | Unclear | Unclear | Unclear | Yes | Yes | No | 4 |
| Viswaprakash N. et al. 2015 | Unclear | Yes | Unclear | Unclear | Unclear | Unclear | Unclear | Yes | Yes | Yes | 4 |
| Woodger T.L. et al 1979 | Yes | Yes | Unclear | Yes | Unclear | Unclear | Unclear | Yes | Yes | No | 5 |
| Xue X. et al. 2017 | Yes | Yes | Unclear | Unclear | Unclear | Unclear | Unclear | Yes | Yes | Yes | 5 |
| Yang Y.F. et al 1995 | Unclear | Yes | Unclear | Unclear | Unclear | Unclear | Unclear | Yes | Yes | Yes | 4 |
| Yehuda R. et al 1984 | Unclear | Yes | Unclear | Unclear | Unclear | Unclear | Unclear | No | Yes | No | 2 |
| Zemdegs J. et al 2018 | Yes | Yes | Unclear | Unclear | Unclear | Unclear | Unclear | Yes | Yes | Yes | 5 |
| Abdel Rasheed N.O. et al. 2018 | Yes | Yes | Unclear | Unclear | Unclear | Unclear | Unclear | Yes | Yes | No | 4 |
| Accardi M.V. et al 2015 | Unclear | Yes | Unclear | Unclear | Unclear | Unclear | Unclear | Yes | Yes | Yes | 4 |
| Adams W.K. et al. 2015 | Unclear | Yes | Unclear | Unclear | Unclear | Unclear | Unclear | No | Yes | Yes | 3 |
| Amin S.N. et al. 2013 | Unclear | Yes | Unclear | Unclear | Unclear | Unclear | Unclear | Yes | Yes | Yes | 4 |
| Sherin A. et al. 2010 | Unclear | Yes | Unclear | Unclear | Unclear | Unclear | Unclear | No | Yes | Yes | 3 |
| Arieff A.I. et al. 1974 | Unclear | Yes | Unclear | Unclear | Unclear | Unclear | Unclear | No | Yes | Yes | 3 |
| Auer M.K. et al 2015 | Unclear | Yes | Unclear | Unclear | Unclear | Unclear | Unclear | Yes | Yes | Yes | 4 |
| Azmitia E.C. et al 1990 | Unclear | Yes | Unclear | Unclear | Unclear | Yes | Yes | Yes | Yes | Yes | 6 |
| Balakrishnan S. et al. 2009 (a) | Unclear | Yes | Unclear | Unclear | Unclear | Unclear | Unclear | No | Yes | Yes | 3 |
| Balakrishnan S. et al 2009 (b) | Unclear | Yes | Unclear | Unclear | Unclear | Unclear | Unclear | No | Yes | Yes | 3 |
| Barber M. et al., 2003 | No | Yes | No | No | No | No | No | Yes | Yes | Yes | 4 |
| Behar K.L. et al., 1984 | No | Unclear | No | No | No | No | No | Unclear | Unclear | Yes | 1 |
| Beverly J.L. et al., 2001 | No | Yes | No | No | No | No | No | Yes | Yes | Yes | 4 |
| Bittencourt A. et al., 2020 | Yes | Yes | No | No | No | No | No | Yes | Yes | Yes | 5 |
| Boychuk C.R. et al., 2015 | No | Yes | No | No | No | No | No | Yes | Yes | Yes | 4 |
| Boychuk C.R. et al., 2016 | No | Yes | No | No | No | No | No | Yes | Yes | Yes | 4 |
| Boychuk C.R. et al., 2017 | Yes | Yes | No | No | No | No | No | Yes | Yes | Yes | 5 |
| Briski K.P. et al., 2020 | Yes | Yes | No | No | No | No | No | Yes | Yes | Yes | 5 |
| Briski K.P. et al., 2021 | Yes | Yes | No | No | No | No | No | Yes | Yes | Yes | 5 |
| Butterworth R.F. et al., 1980 | No | Yes | No | No | No | No | No | Yes | Yes | Unclear | 3 |
| Butterworth R.F. et al., 1982 | No | Yes | No | No | No | No | No | Yes | Yes | Unclear | 3 |
| Cardoso S. et al, 2011 | Yes | Yes | No | No | No | No | No | Unclear | Yes | Yes | 4 |
| Chan O.et al., 2013 | No | Unclear | No | No | No | No | No | Yes | Yes | Yes | 3 |
| Chowdhury G.M.I. et al., 2017 | No | Yes | No | No | No | No | No | Yes | Yes | Yes | 4 |
| Coleman E.S. et al., 2010 | No | Yes | No | No | No | No | No | Yes | Yes | Yes | 4 |
| Costello D.A. et al., 2012 | No | No | No | No | No | No | No | Yes | Yes | Yes | 3 |
| D'Souza S.S. et al., 2016 | No | Yes | No | No | No | No | No | Yes | Yes | Unclear | 3 |
| Darling B.K. Et al., 2001 | No | Yes | No | No | No | No | No | Yes | Yes | Yes | 4 |
| Datusalia A.K. Et al., 2018 | No | Yes | No | No | No | No | No | Yes | Yes | Yes | 4 |
| Del Rio D. et al., 2016 | No | Yes | No | No | No | No | No | Yes | Yes | Yes | 4 |
| Di Luca M. et al., 1999 | No | Yes | No | No | No | No | No | Yes | Yes | Yes | 4 |
| Duarte A. et al., 2000 | No | No | No | No | No | No | No | Yes | Yes | Yes | 3 |
| Duarte A.I. et al., 2003 | No | Yes | No | No | No | No | No | Yes | Yes | Yes | 4 |
| Duarte J.M.N. et al., 2012 | Yes | No | No | No | No | No | No | Yes | Yes | Yes | 4 |
| Fernstrom J.D. et al., 1971 | No | Unclear | No | No | No | No | No | Unclear | Yes | Unclear | 1 |
| Figlewicz D.P. et al., 2002 | Yes | Yes | No | No | No | No | No | Yes | Yes | Yes | 5 |
| Fine J.M. et al., 2019 | Yes | Yes | No | No | No | Yes | No | Yes | Yes | Yes | 6 |
| Gardoni F. et al., 2002 | No | Yes | No | No | No | No | No | Yes | Yes | Yes | 4 |
| Girault F-M et al., 2017 | No | No | No | No | No | No | No | Yes | Yes | Yes | 3 |
| Gordon A.E. et al., 1970 | No | Unclear | No | No | No | No | No | Unclear | Yes | Unclear | 1 |
| Gorell J.M. et al., 1976 | No | Yes | No | No | No | No | No | Unclear | Yes | Unclear | 2 |
| Grillo C.A. et al., 2015 | No | Yes | No | No | No | No | No | Yes | Yes | Yes | 4 |
| Gundersen V. et al., 2001 | No | Yes | No | No | No | No | No | Yes | Yes | Yes | 4 |
| Guzman D.C. et al., 2014 | No | Yes | No | No | No | No | No | Yes | Yes | Yes | 4 |
| Hammoud H. et al., 2020 | No | Yes | No | No | No | No | No | Yes | Yes | Yes | 4 |
| Hawkins R.A. et al., 2010 | No | Yes | No | No | No | No | No | Yes | Yes | Yes | 4 |
| Herrera R. et al., 2005 | No | Unclear | No | No | No | No | No | Unclear | Yes | Unclear | 2 |
| Jangra A. et al., 2013 | No | Yes | No | No | No | No | No | Yes | Yes | Yes | 4 |
| Jin L. et al., 2018 | Yes | Yes | No | No | No | No | No | Yes | Yes | Yes | 5 |
| Jin Z. et al., 2011 | No | Yes | No | No | No | No | No | Yes | Yes | Yes | 4 |
| Jones K.T. et al., 2017 | No | Yes | No | No | No | No | No | Yes | Yes | Yes | 4 |
| Joseph A. et al., 2008 | No | Yes | No | No | No | No | No | Yes | Yes | Yes | 4 |
| Kamei J. Et al., 1997 | No | Unclear | No | No | No | No | No | Unclear | Yes | Unclear | 1 |
| Kawamura N. et al., 2021 | Yes | Yes | No | No | No | No | No | Yes | Yes | Yes | 5 |
| Kim S-J. Et al., 2005 | No | Yes | No | No | No | No | No | Yes | Yes | Yes | 4 |
| Knusel B. et al., 1991 | No | Yes | No | No | No | No | No | Yes | Yes | Yes | 4 |
| Kwok R.P.S. et al., 1985 | No | No | No | No | No | No | No | Yes | Yes | Yes | 3 |
| Labak M. et al., 2010 | No | Yes | No | No | No | No | No | Yes | Yes | Yes | 4 |
| Liu J. et al., 2020 | No | Yes | No | No | No | No | No | Yes | Yes | Yes | 4 |
| Liu X. et al., 2019 | No | Yes | No | No | No | No | No | Yes | Yes | Yes | 4 |
| Lizarbe B. et al.,2019 | Yes | Yes | No | No | No | yes | No | Yes | Yes | Yes | 6 |
| Matsunga Y. et al., 2016 | No | No | No | No | No | No | No | Yes | Yes | Yes | 3 |
| McGowan J.E. et al., 2001 | Yes | Yes | No | No | No | No | No | Yes | Yes | Yes | 5 |
| McNeilly A.D. et al., 2014 | Yes | Yes | No | No | No | No | Yes | Yes | Yes | Yes | 6 |
| Mebel D.M. et al., 2012 | No | Yes | No | No | No | No | No | Yes | Yes | Yes | 4 |
| Mechlovich D. et al., 2014 | Yes | Yes | No | No | No | No | No | Yes | Yes | Yes | 5 |
| Muller A.P. et al., 2010 | No | Yes | No | No | No | No | No | Yes | Yes | Yes | 4 |
| Murtishaw A.S. et al., 2015 | Yes | Yes | No | No | No | No | No | Yes | Yes | Yes | 5 |
| Nair A.R. et al., 1998 | Yes | Yes | No | No | No | No | No | Yes | Yes | Yes | 5 |
| Nair P. et al., 1996 | Yes | Yes | No | No | No | No | No | Unclear | Yes | Unclear | 3 |
| Nardin P. et al., 2016 | No | Yes | No | No | No | Yes | No | Yes | Yes | Yes | 5 |
| Paulose C.S. et al., 2008 | No | Yes | No | No | No | No | No | Yes | Yes | Yes | 4 |
| Paulsen R. et al., 1986 | No | Yes | No | No | No | No | No | Yes | Yes | Unclear | 3 |
| Petroff O.A.C. et al., 1987 | Yes | Yes | No | No | No | No | No | Yes | Yes | Yes | 5 |
| Poblete-Naredo I. et al., 2009 | No | Yes | No | No | No | No | No | Yes | Unclear | Yes | 3 |
| Porter J.R. et al, 1997 | No | Yes | No | No | No | No | No | Yes | Yes | Unclear | 3 |
| Rusnàk M. et al., 1998 | No | Yes | No | No | No | No | No | Yes | Yes | Unclear | 3 |
| Sasaki-Hamada S. et al., 2015 | No | Yes | No | No | No | No | No | Yes | Yes | Yes | 4 |
| Sherin A. et al., 2010 | No | Yes | No | No | No | No | No | Yes | Yes | Yes | 4 |
| Shinbori C. et al., 2008 | Yes | Yes | No | No | No | No | No | Yes | Yes | Yes | 5 |
| Shonesy B.C. et al., 2012 | Yes | Yes | No | No | No | No | No | Yes | Yes | Yes | 5 |
| Smythe G.A. et al., 1984 | No | Yes | No | No | No | No | No | Yes | Yes | Unclear | 3 |
| Spinelli M. et al., 2009 | Yes | Yes | No | No | Yes | Yes | Unclear | Yes | Yes | Yes | 7 |
| Suzuki M. et al., 2012 | No | Yes | No | No | No | No | No | Yes | Yes | Yes | 4 |
| Teleushkin P.K. et al., 2005 | No | Yes | No | No | No | No | No | Unclear | Yes | Yes | 3 |
| Tsai S-F. et al., 2018 | Yes | Yes | No | No | Yes | No | Unclear | Yes | Yes | Yes | 6 |
| Tu L-L. et al. 2019 | Yes | No | No | No | No | Yes | No | Yes | Yes | Yes | 5 |
| Valastro B. et al., 2002 | No | Yes | No | No | No | No | No | Yes | Yes | Yes | 4 |
| van der Heide L.P. et al., 2005 | No | Yes | No | No | No | No | No | Yes | Yes | Yes | 4 |
| Wan Q. et al., 1997 | No | Yes | No | No | No | No | No | Yes | Yes | Yes | 4 |
| Wesselmann U. et al., 1987 | No | Yes | No | No | No | No | No | Yes | Yes | Yes | 4 |
| Williams J.M. et al., 2007 | No | Yes | No | No | No | No | No | Yes | Yes | Yes | 4 |
| Zhang H. et al., 2015 | No | Yes | No | No | No | No | No | Yes | Yes | Unclear | 3 |
| Agardh C-D. et al, 1978 | No | Yes | No | No | No | No | No | Yes | Yes | Yes | 4 |
| Brass B.J. Et al., 1992 | No | Yes | No | No | No | No | No | Yes | Yes | Unclear | 3 |
| Duarte A.I. et al., 2004 | No | No | No | No | No | No | No | Yes | Yes | Yes | 3 |
| Engelsen B. et al., 1986 | No | Yes | No | No | No | No | No | Yes | Yes | Unclear | 3 |
| Guyot L.L. et al., 1999 | No | Yes | No | No | No | No | No | Yes | Yes | Unclear | 3 |
| Hernandez T. et al., 1968 | No | Yes | No | No | No | No | No | Unclear | Yes | Unclear | 2 |
| Jackson J. et al., 1999 | Yes | Yes | No | No | No | No | No | Yes | Yes | Yes | 5 |
| Jackson J. et al., 2000 | Yes | Yes | No | No | No | No | No | Yes | Yes | Yes | 5 |
| Jhanwar-Uniyal M. et al., 1994 | Yes | Yes | No | No | No | No | No | Yes | Yes | Yes | 5 |
| Mackenzie R.G. et al., 1978 | No | Yes | No | No | No | No | No | Yes | Yes | Yes | 4 |
| Plitzko D. et al., 2001 | No | Yes | No | No | No | No | No | Yes | Yes | Yes | 4 |
| Ramakrishnan R. et al., 2004 | No | Yes | No | No | No | No | No | Yes | Yes | Yes | 4 |
| Regalia J. Et al., 2001 | No | Yes | No | No | No | No | No | Yes | Yes | Yes | 4 |
| Rusnàk M. et al., 2001 | No | Yes | No | No | No | No | No | Yes | Yes | Yes | 4 |
| Saad S.F., 1972 | No | Yes | No | No | No | No | No | Yes | Yes | Unclear | 3 |
| Sena A. et al., 1988 | No | Yes | No | No | No | No | No | Unclear | Yes | Unclear | 2 |
| Shiraishi J-I et al., 2010 | No | Yes | No | No | No | No | No | Unclear | Yes | Unclear | 2 |
| Spicarova D. et al., 2010 | No | Yes | No | No | No | No | No | Yes | Yes | Yes | 4 |
| Tholey G. et al., 1986 | No | Unclear | No | No | No | No | No | Unclear | Yes | Yes | 2 |
| Tossman U. et al., 1985 | No | Unclear | No | No | No | No | No | Unclear | Unclear | Yes | 1 |

Quality rating for included animal studies was performed according to the Systematic Review Centre for Laboratory Animal Experimentation (SYRCLE) Tool.

| Author(s), year | Study design | NIH  1 | NIH  2 | NIH  3 | NIH  4 | NIH  5 | NIH  6 | NIH  7 | NIH  8 | NIH  9 | NIH  10 | NIH  11 | NIH  12 | NIH  13 | NIH  14 | NIH total |
| --- | --- | --- | --- | --- | --- | --- | --- | --- | --- | --- | --- | --- | --- | --- | --- | --- |
| Haley A.P. et al., 2010 | Observational | Yes | Yes | Yes | Yes | Yes | Yes | Yes | NR | Yes | No | Yes | No | Yes | Yes | 11 |
| Fried P.J. et al., 2019 | Observational | Yes | Yes | Yes | Yes | Yes | Yes | Yes | NR | Yes | No | Yes | No | Yes | Yes | 11 |
| Thanarajah S.E. et al., 2019 | RCT | Yes | Yes | No | Yes | No | Yes | Yes | yes | Yes | Yes | Yes | CD | Yes | No | 10 |
| Thielen J-W. et al., 2019 | Observational | Yes | Yes | Yes | Yes | No | Yes | Yes | NR | Yes | No | Yes | No | Yes | Yes | 10 |
| van der Zwaal E.M. et al., 2016 | Observational | Yes | No | Yes | Unclear | Yes | Yes | Yes | No | Unclear | Unclear | Unclear | Unclear | Unclear | No | 7 |
| Ruth I. Versteeg et al., 2016 | Case-control | Yes | Yes | Unclear | Unclear | Unclear | No | Unclear | Yes | No | Yes | No | Yes | NA | NA | 5 |
| Kullmann et al., 2021 | RCT | Yes | Yes | Yes | Yes | Yes | Yes | Yes | Yes | Yes | Yes | Yes | Unclear | Yes | Yes | 13 |

**Supplementary Table 2: NIH quality assessment of included human studies**

Quality rating for included human studies was performed according to the National Institutes of Health (NIH) Study Quality Assessment Tool. CD: cannot determine; NA: not applicable; NR: not reported; RCT: randomized clinical trial.

**Supplementary Figure n.1 PRISMA Checklist
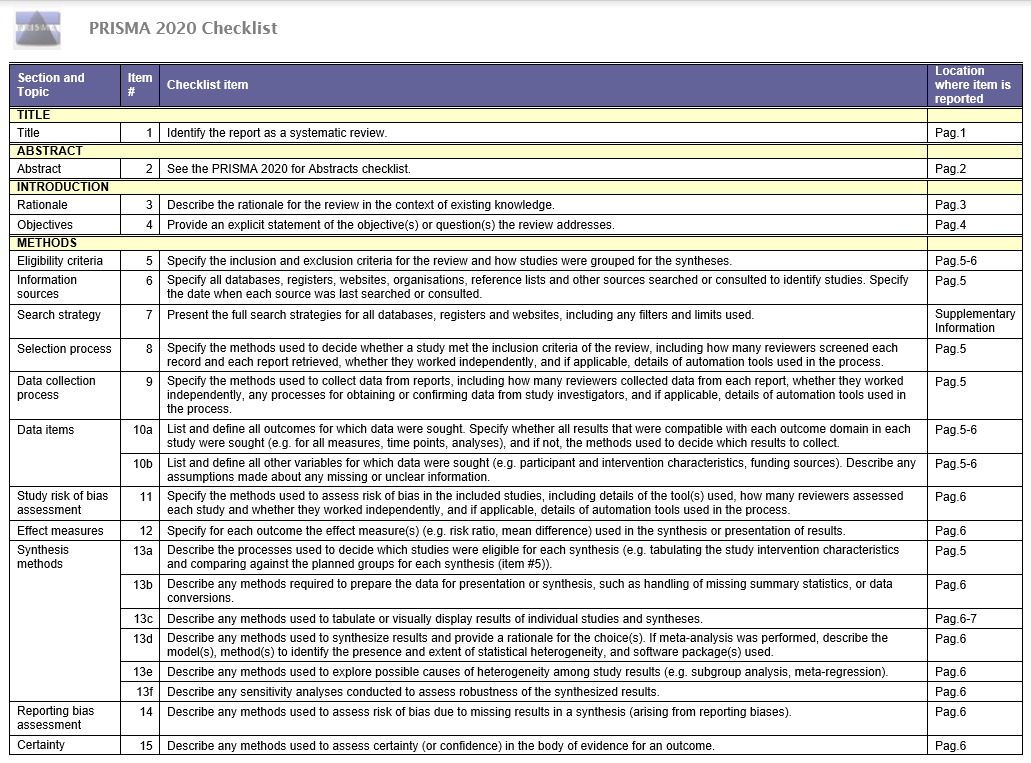
**


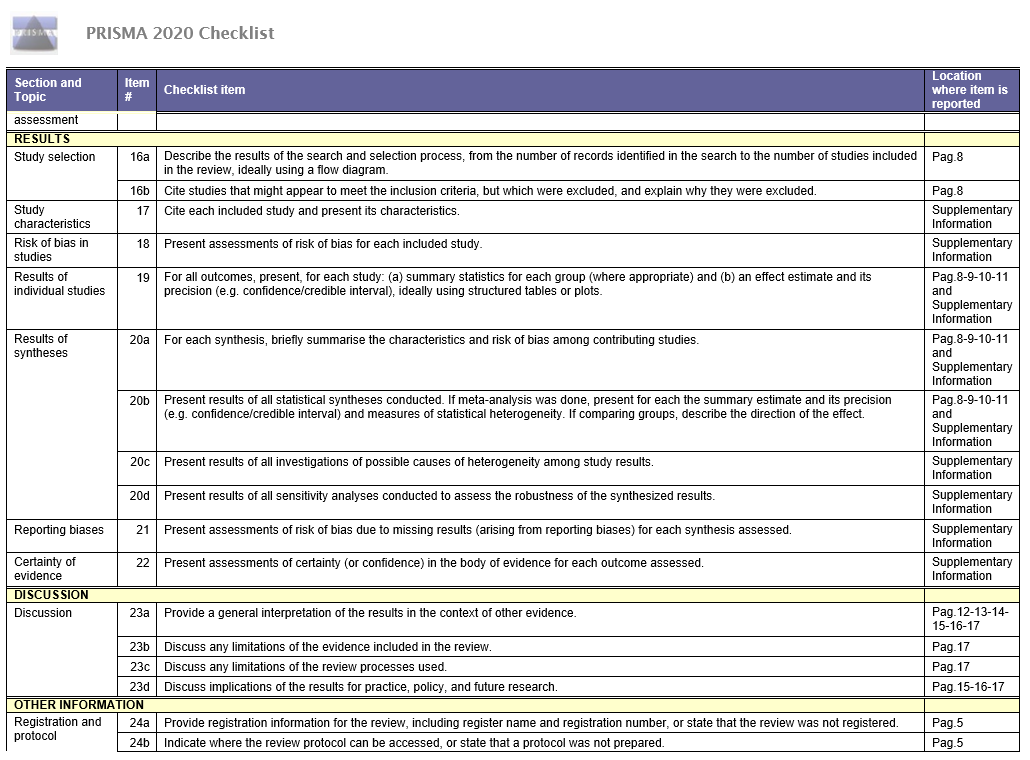


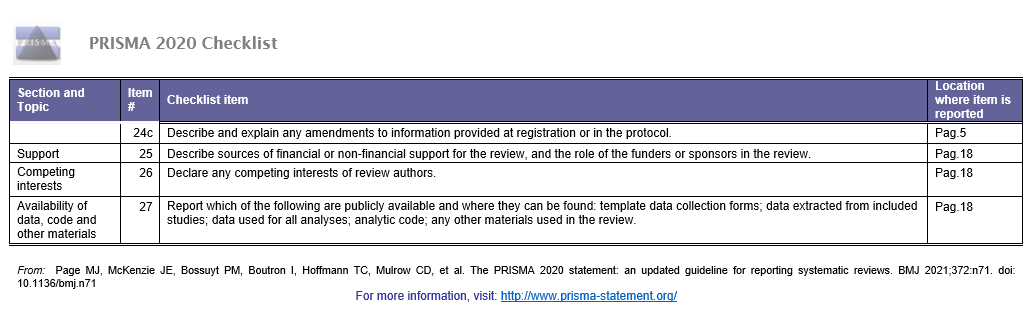


**Supplementary Figure n.2 - 3-MT concentration in hyperinsulinemia, main analysis**

**
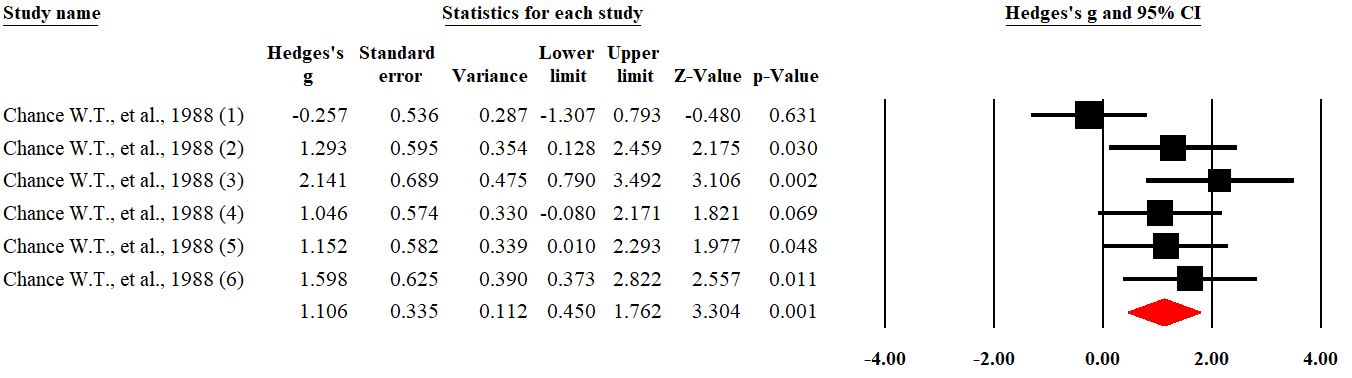
**


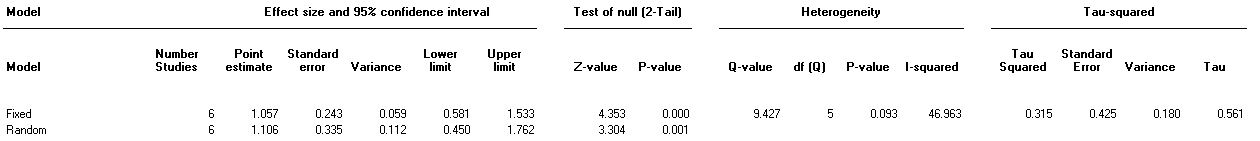


**Supplementary Figure n.3 - DAT Vmax in hyperinsulinemia, main analysis**

**
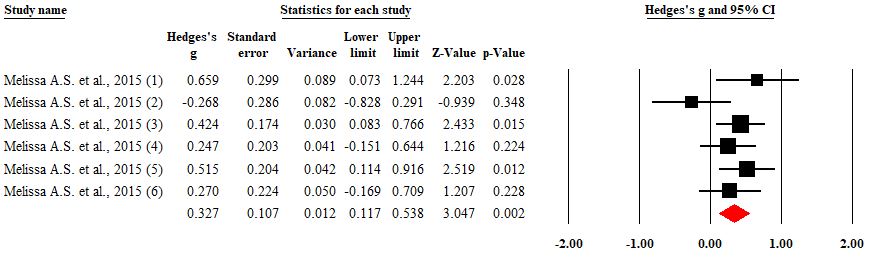
**


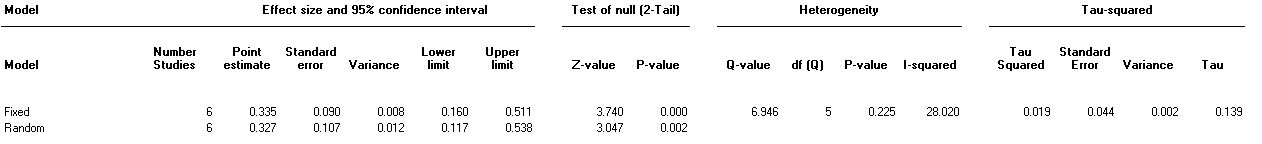


**Supplementary Figure n.4 - DOPA accumulation in hyperinsulinemia, main and subgroup analysis, publication bias, and meta-regression**

**
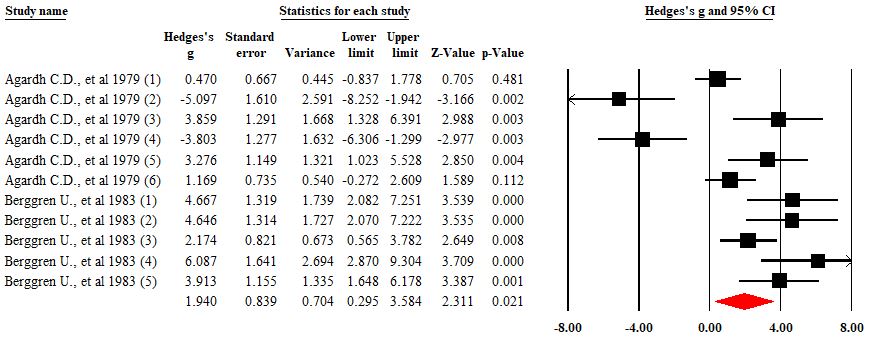
**


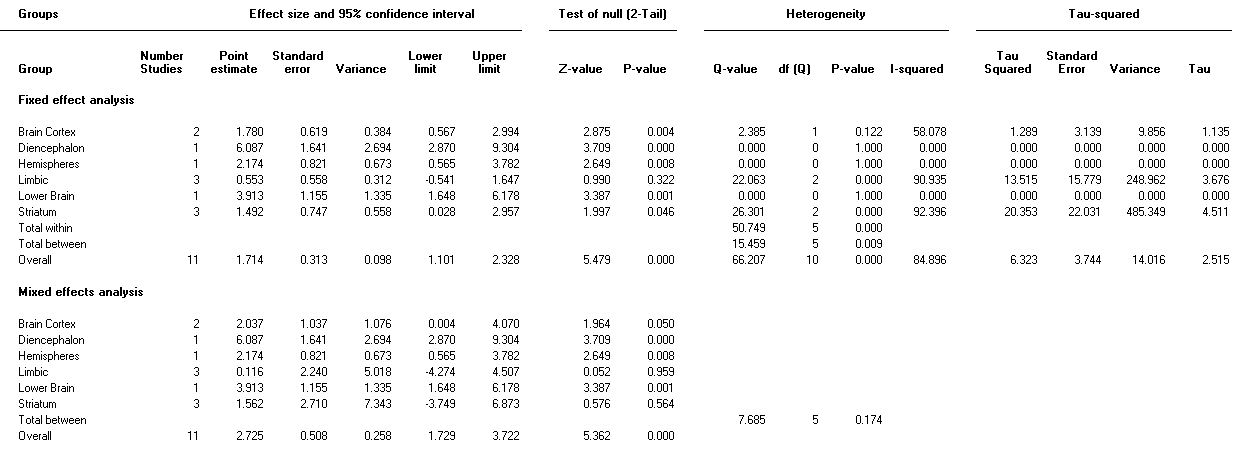


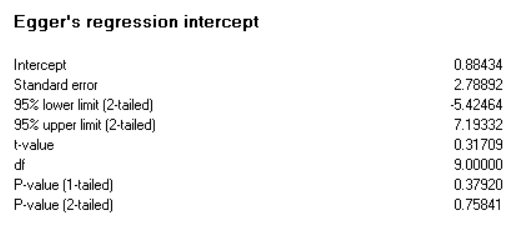


**
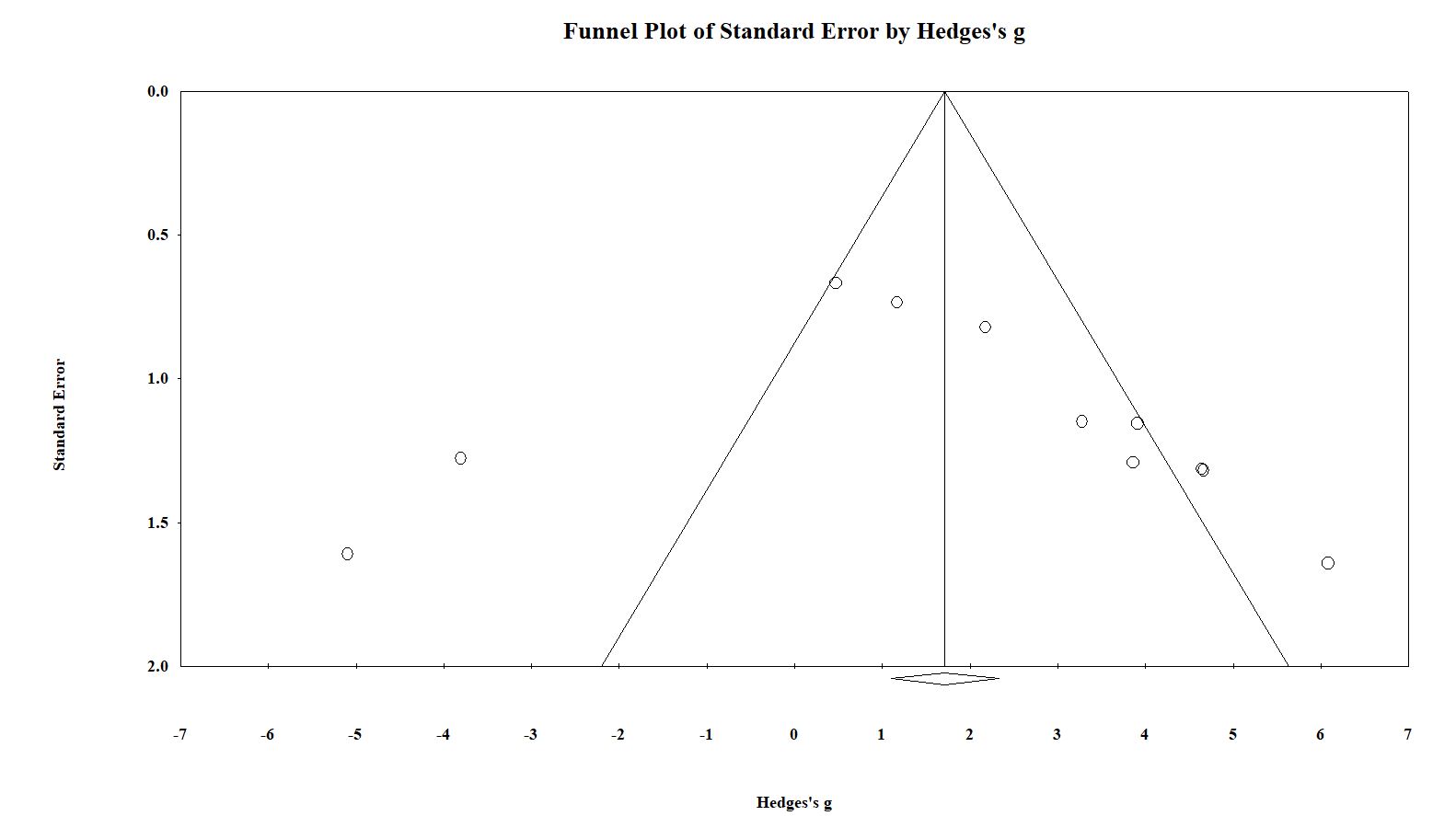
**

**Meta-regression of time from insulin injection**


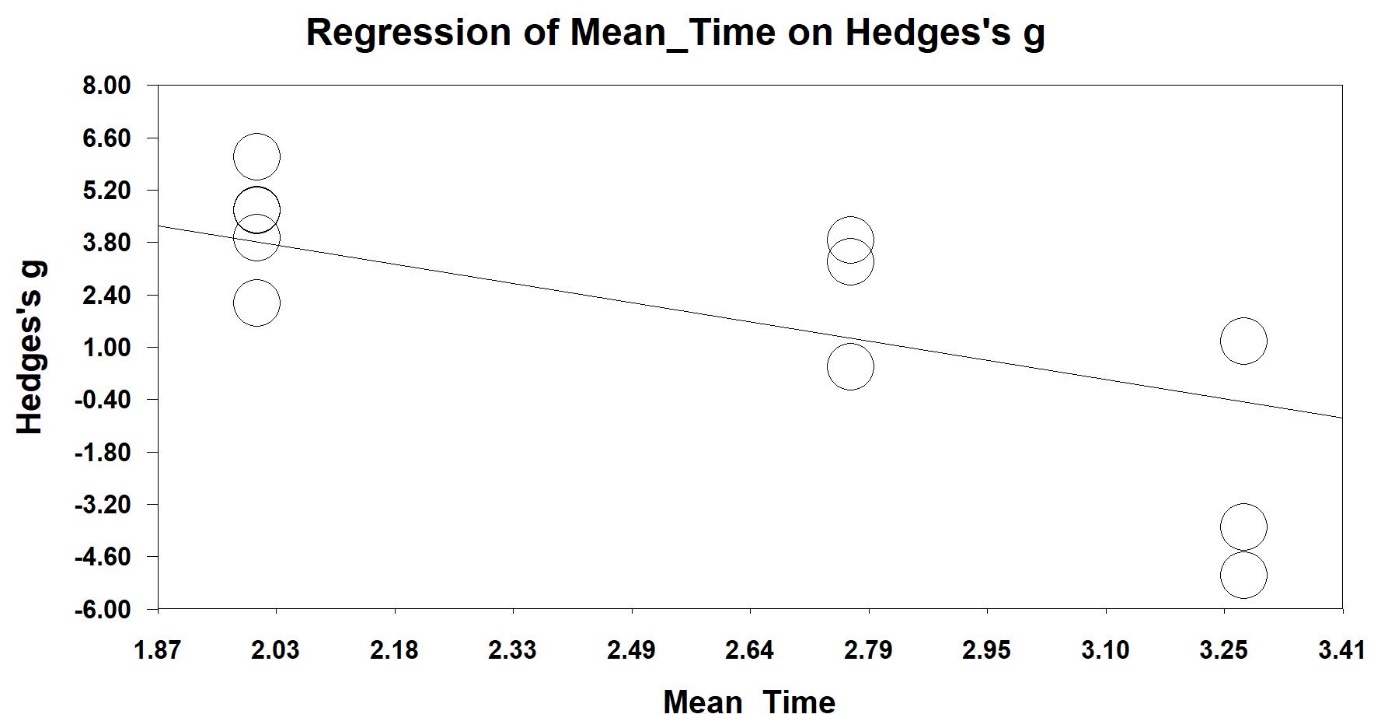


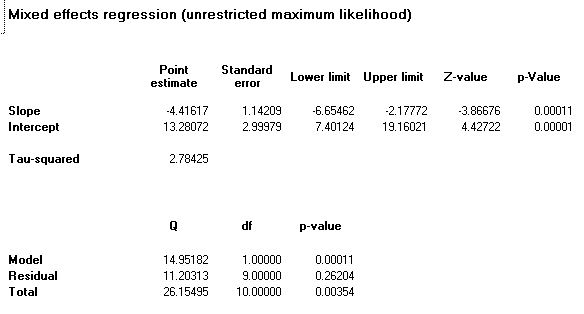


**Supplementary Figure n.5 - DOPAC concentration in hyperinsulinemia, main and subgroup analysis, publication bias, and meta-regression**


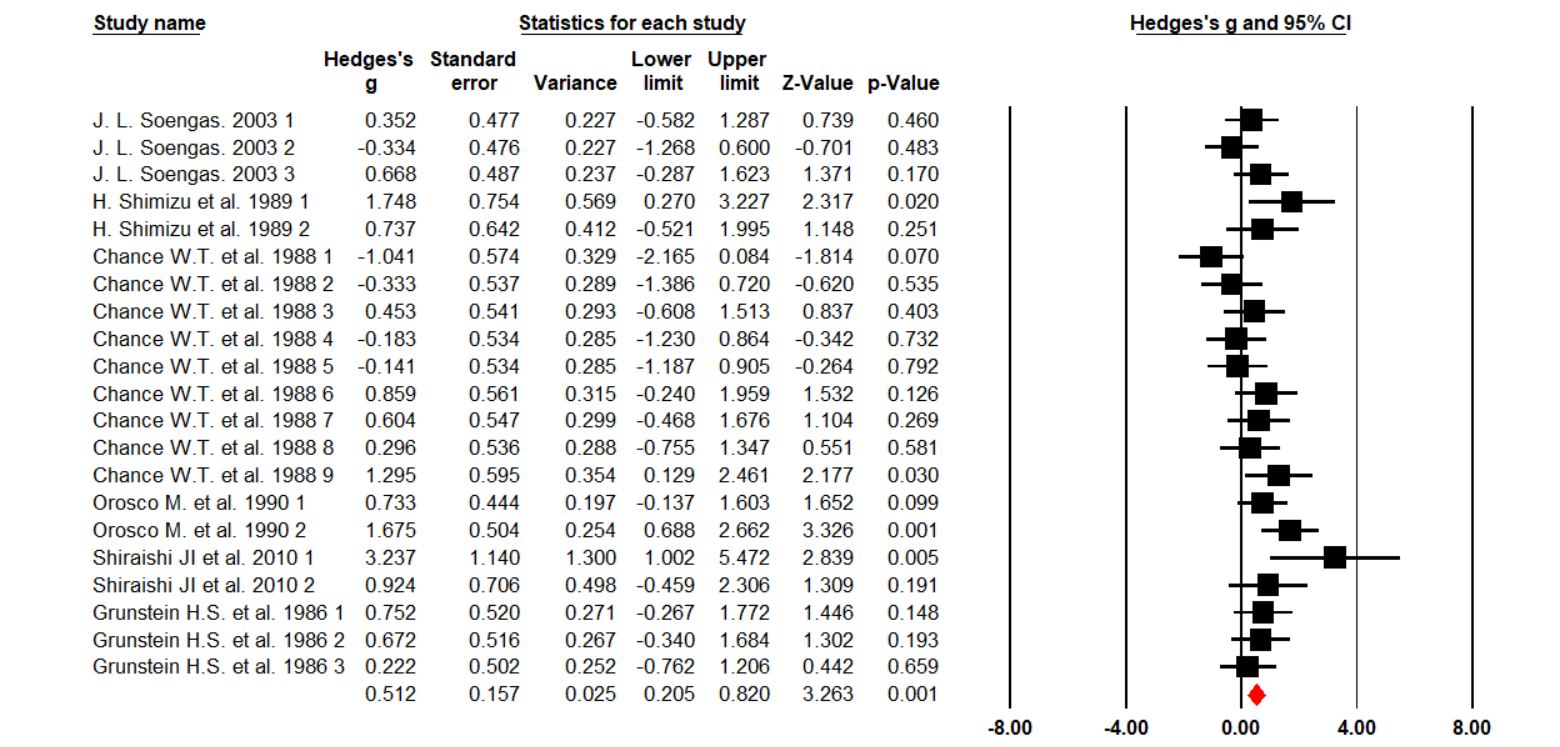


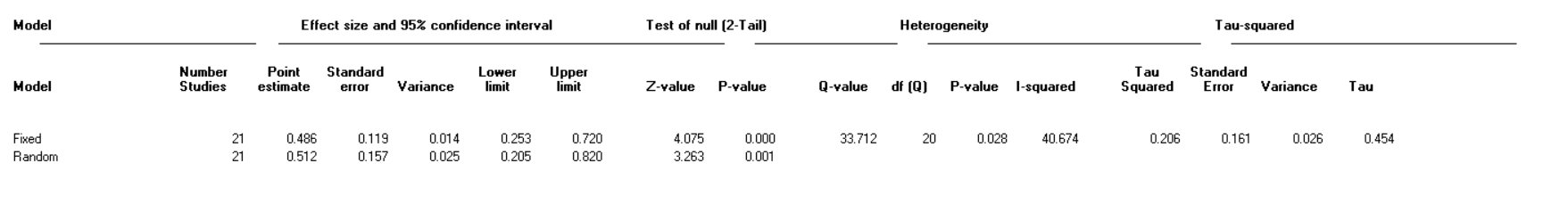


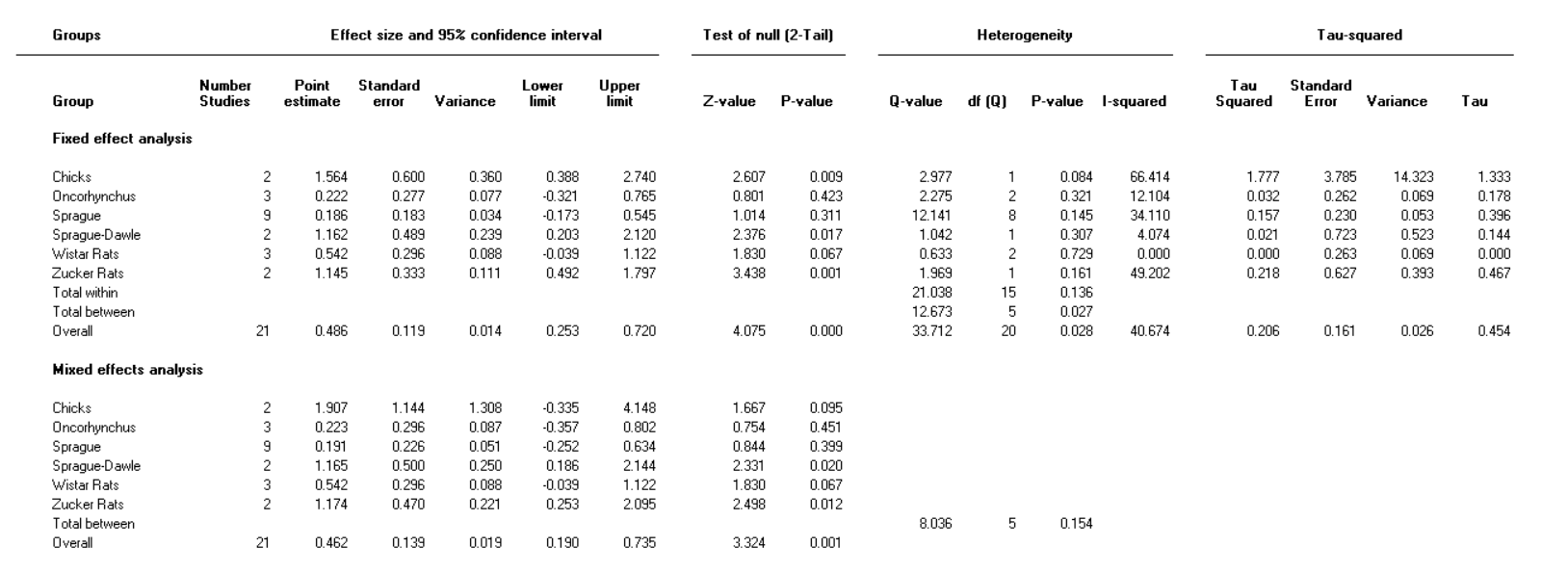


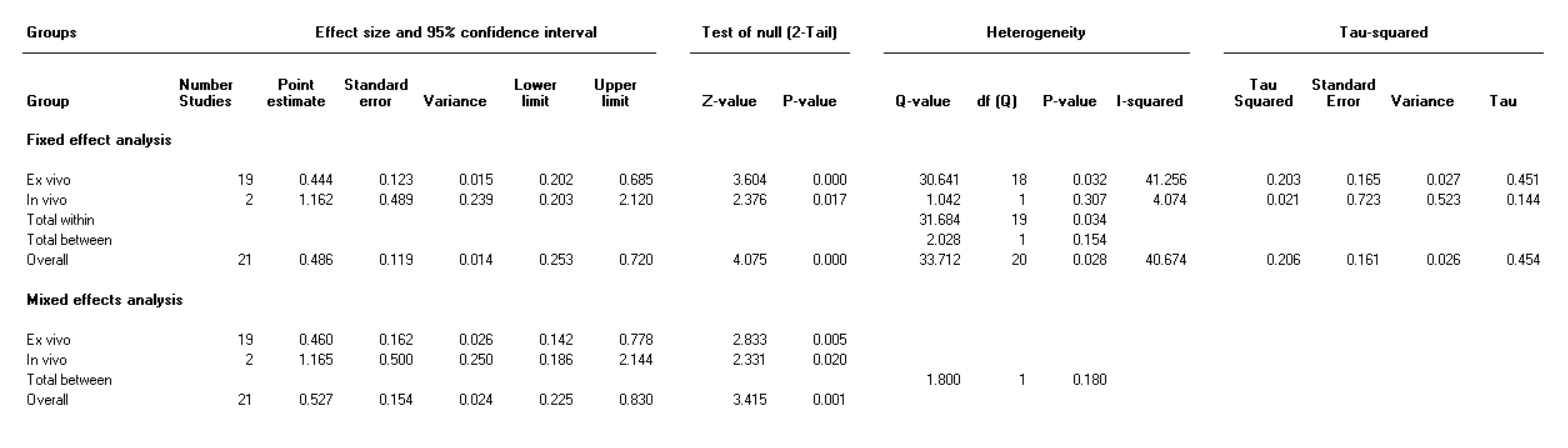


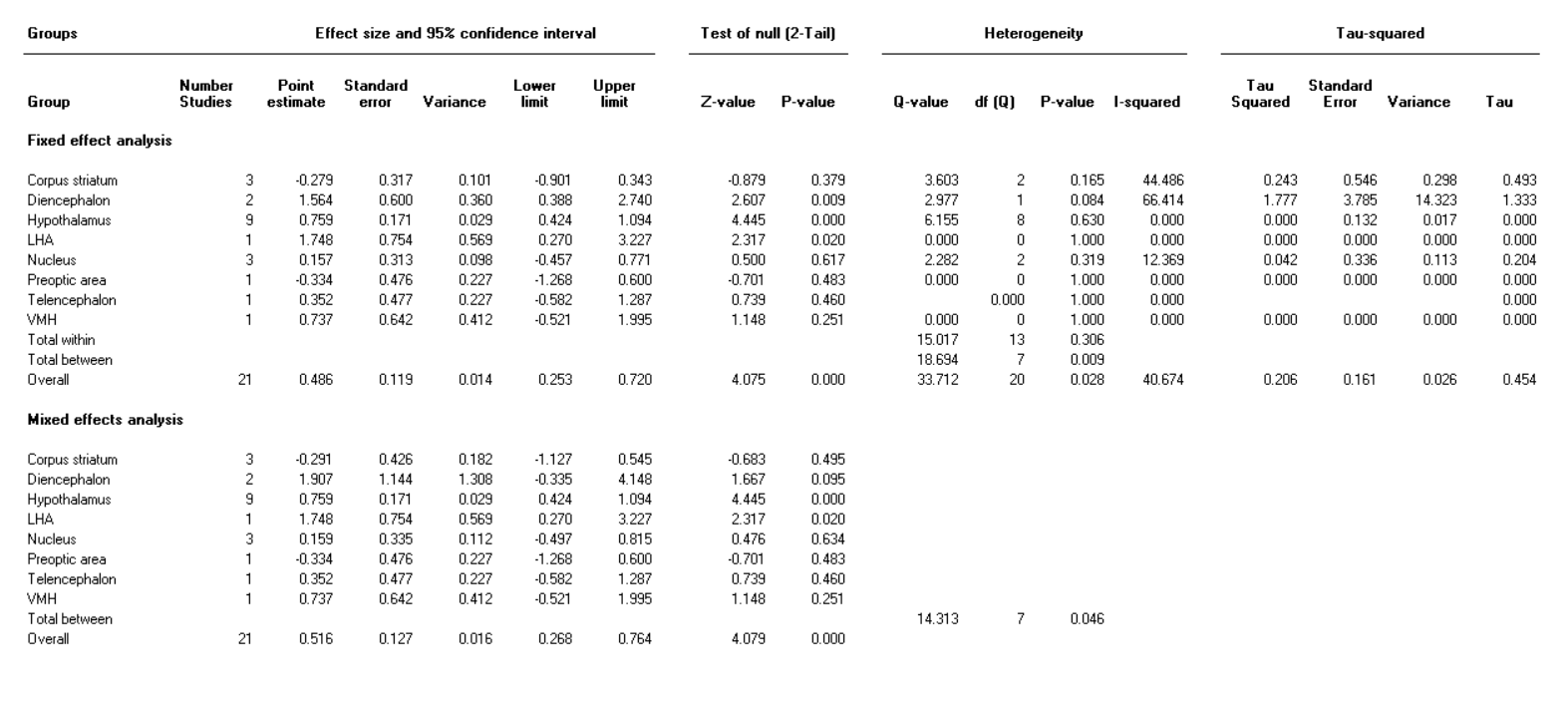


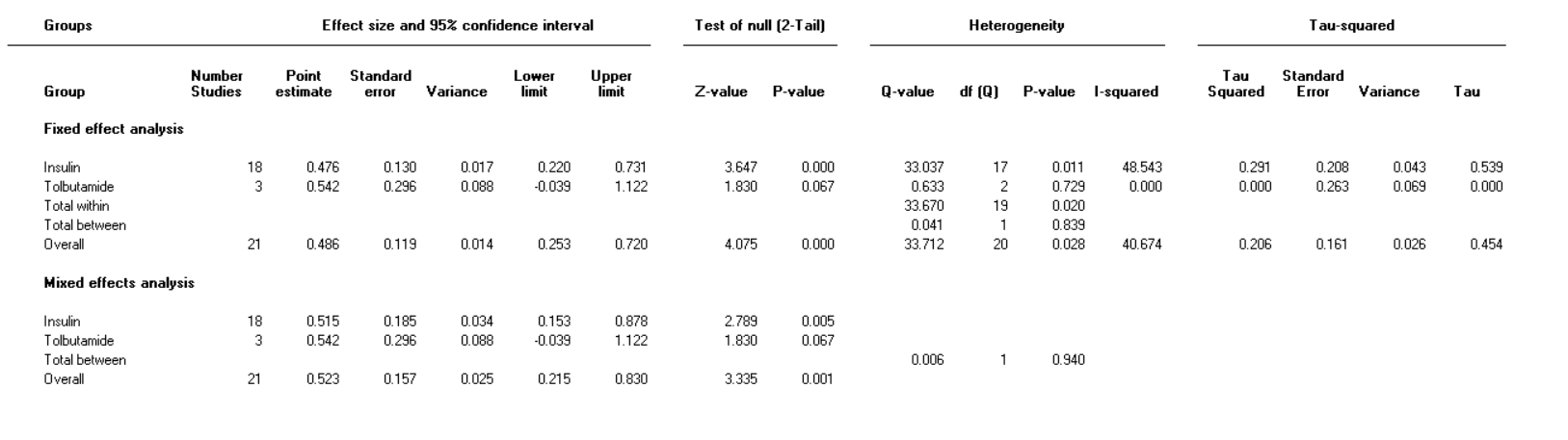


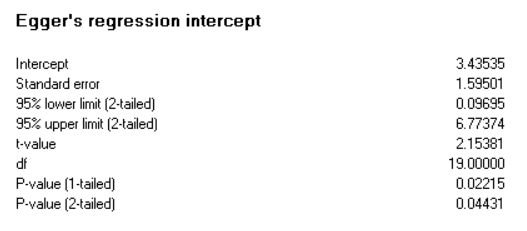


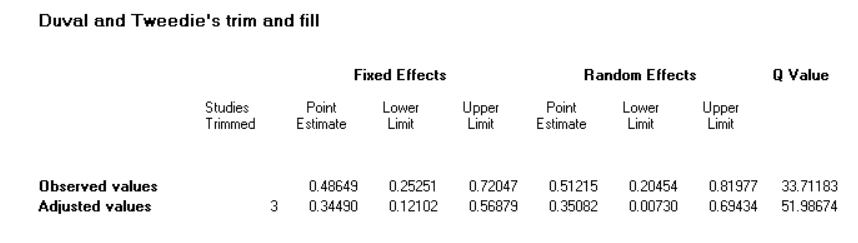


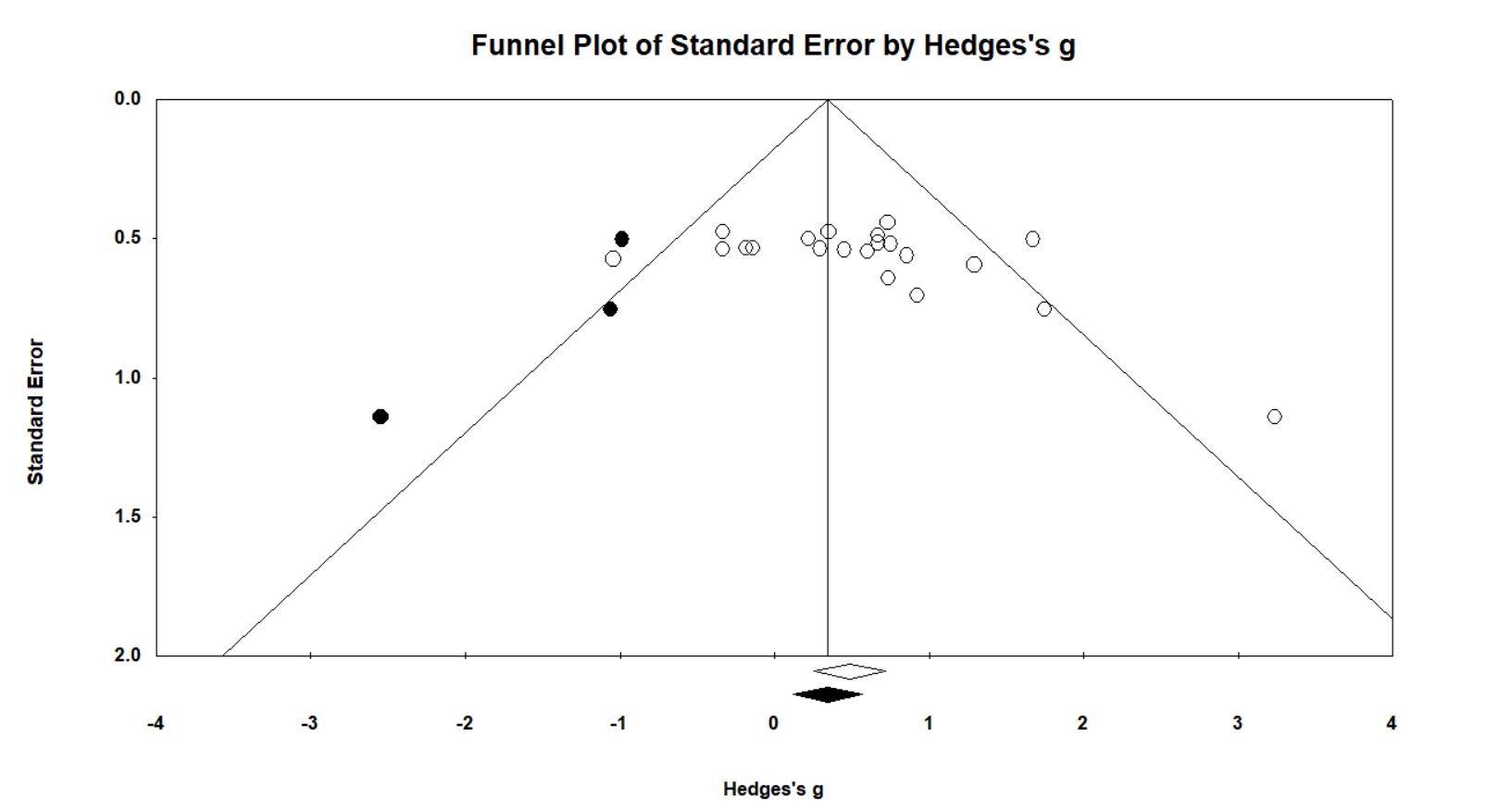


**
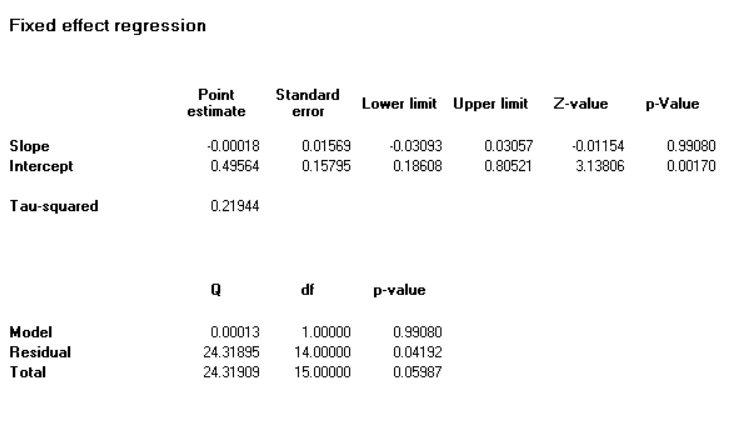
**

**Meta-regression of time from insulin administration rats**

**
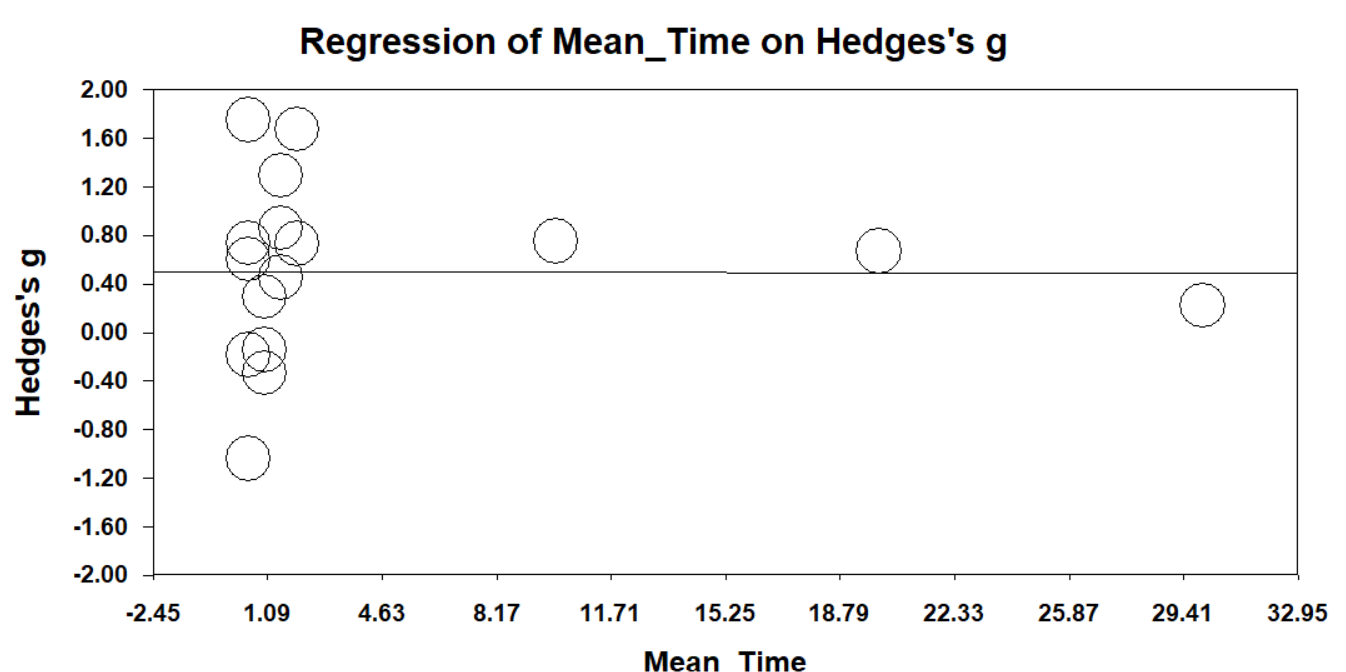
**

**Meta-regression of blood glucose ratio (cases/controls)**

**
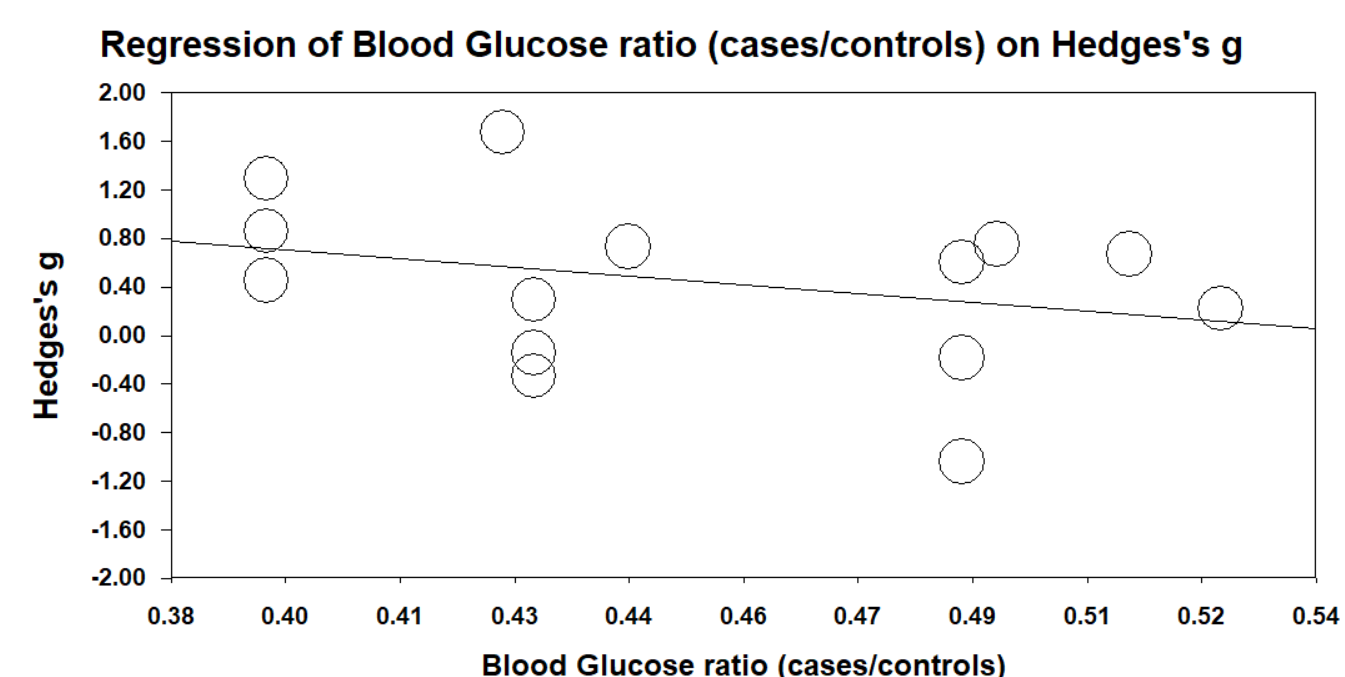
**

**
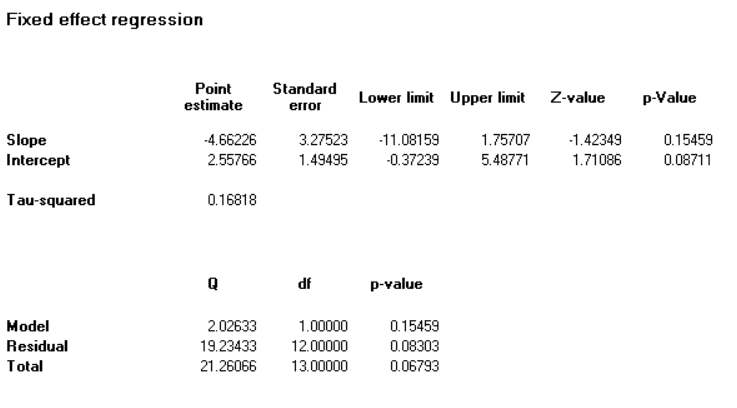
**

**Supplementary Figure n.6 - Dopamine-Beta-Hydroxylase (DβH) Protein Expression in hyperinsulinemia, main and subgroup analysis**

**
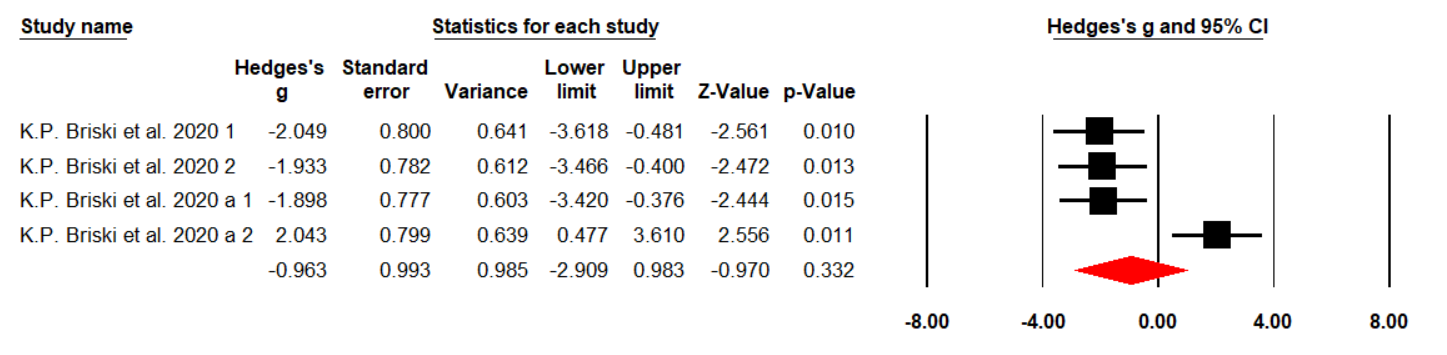
**

**
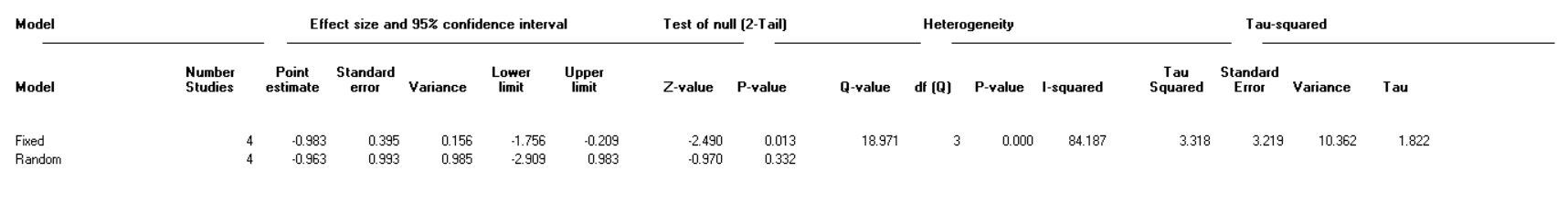
**

**Supplementary Figure n.7 - Dopamine concentration in hyperinsulinemia, main and subgroup analysis, publication bias, and meta-regression**

**
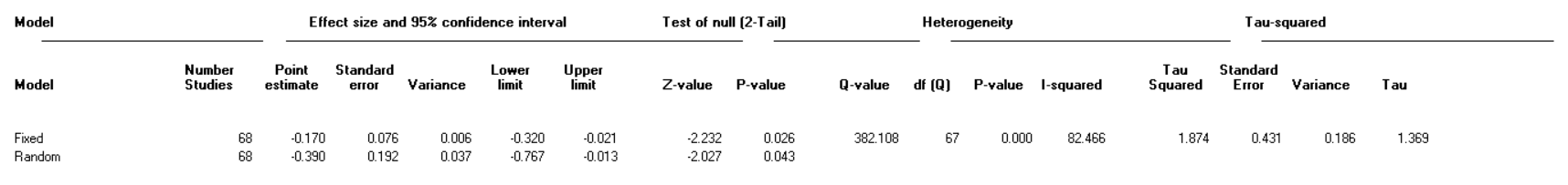
**

**
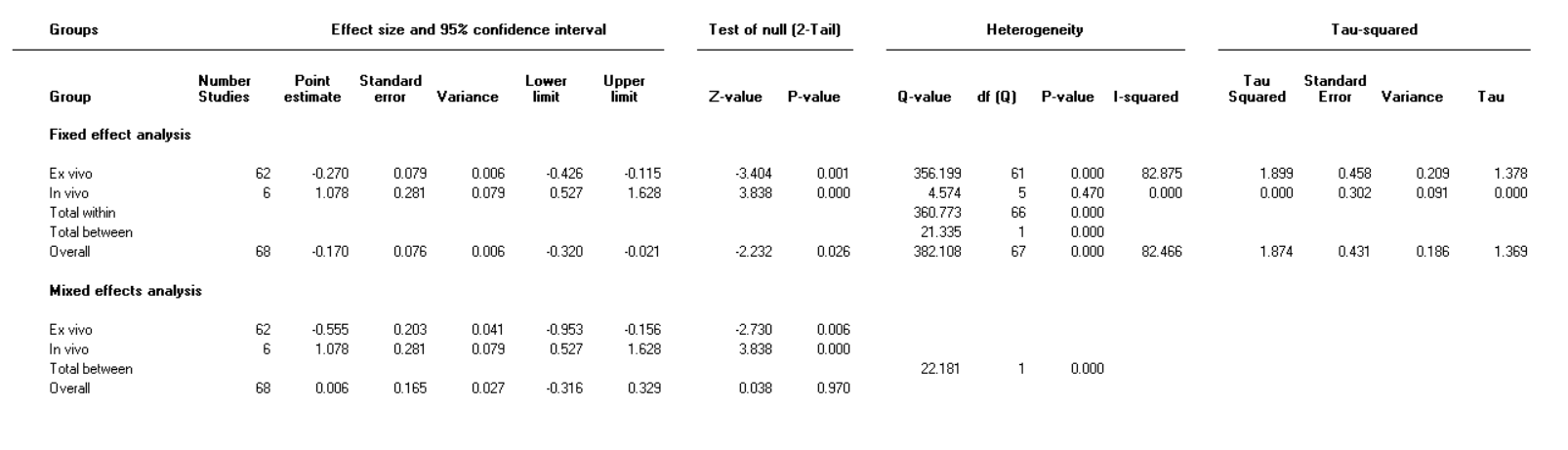
**

**
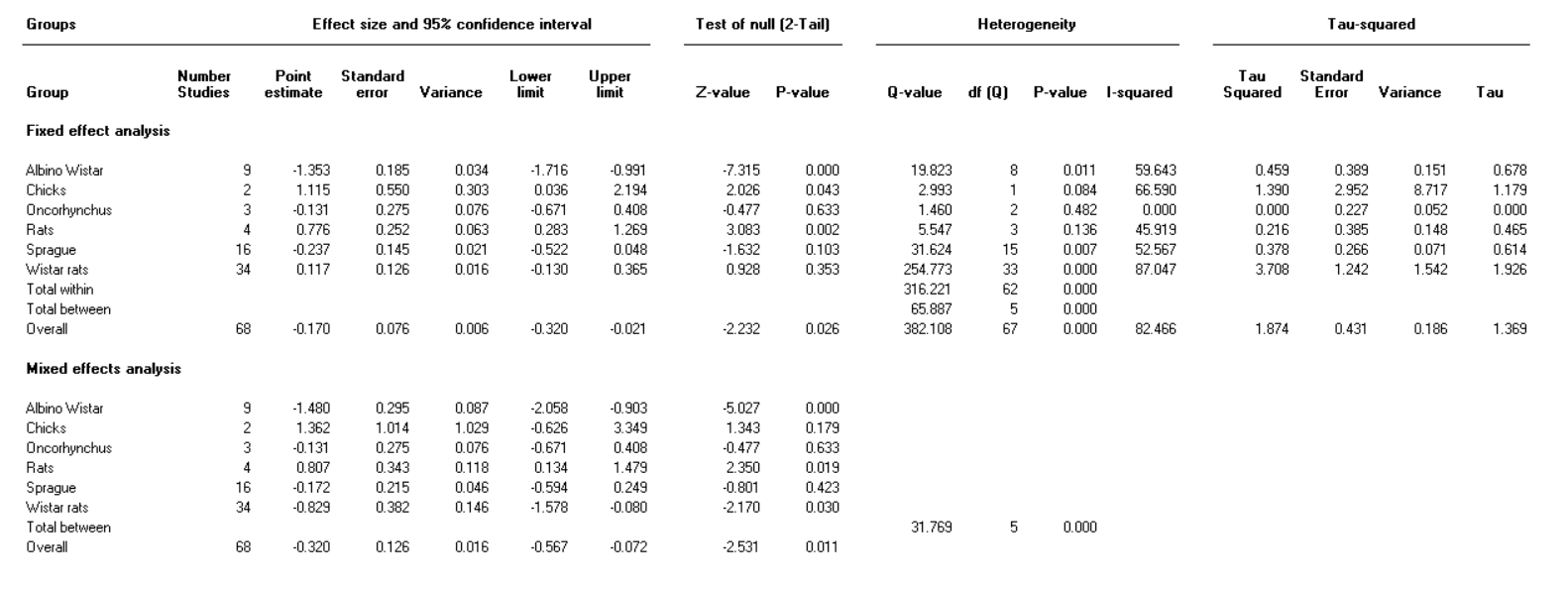
**

**
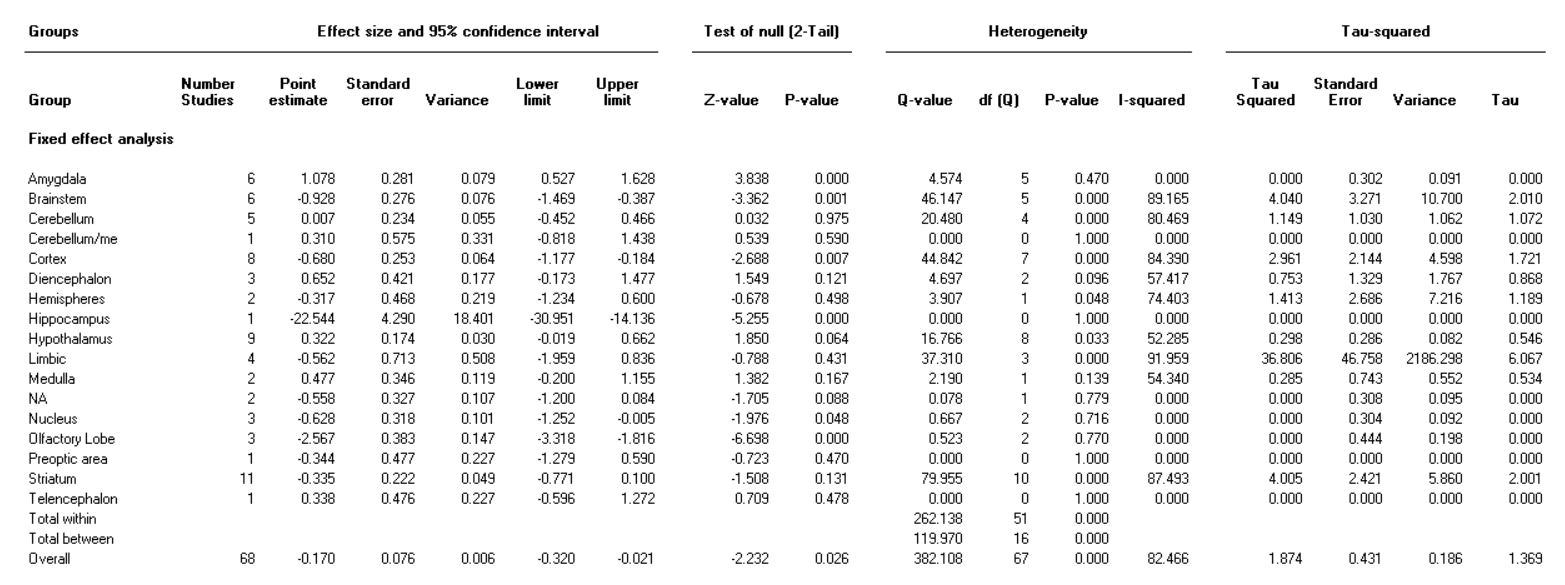
**

**
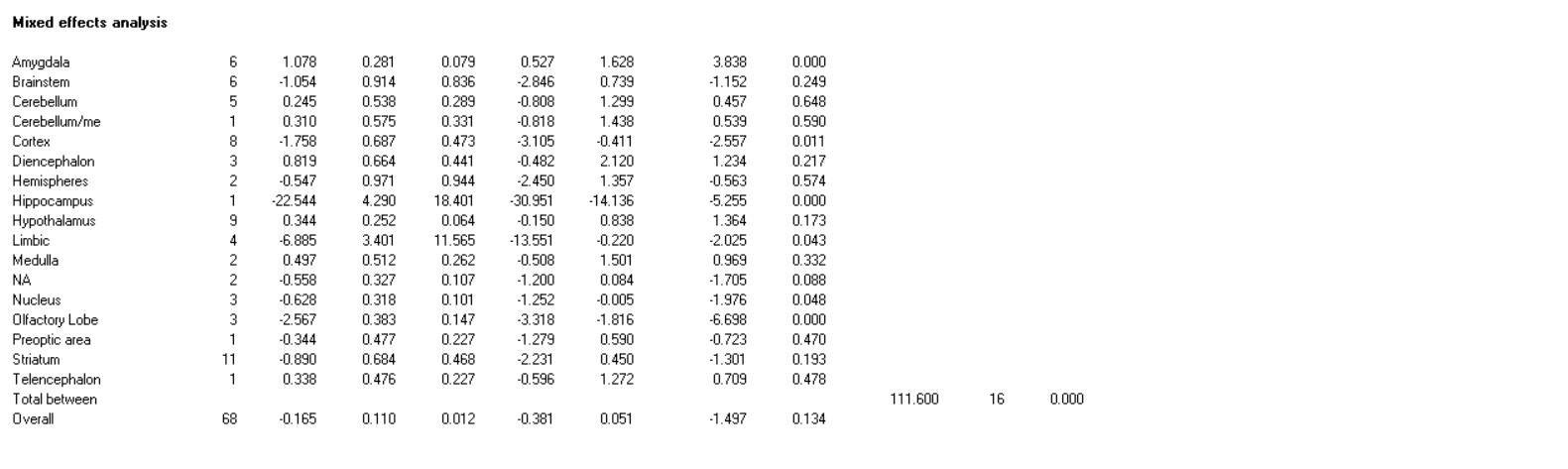
**

**
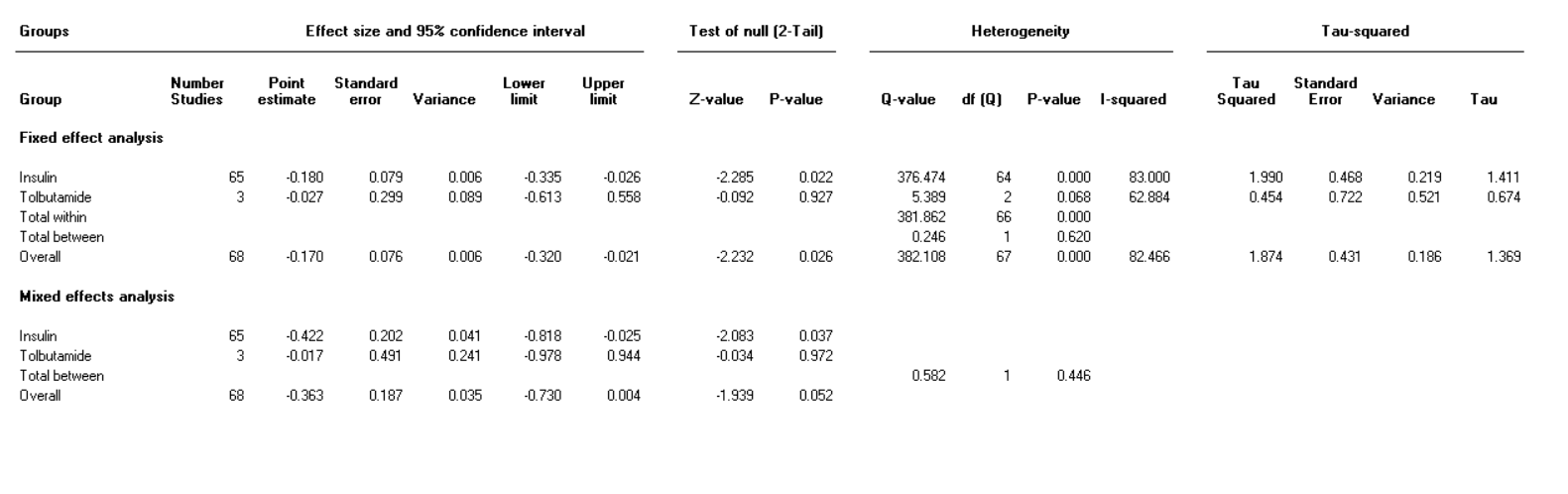
**

**
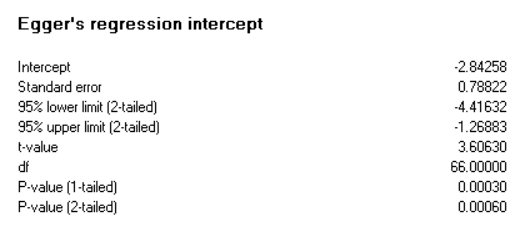
**

**
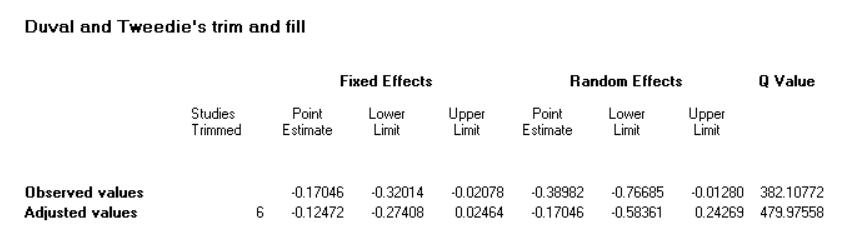
**

**
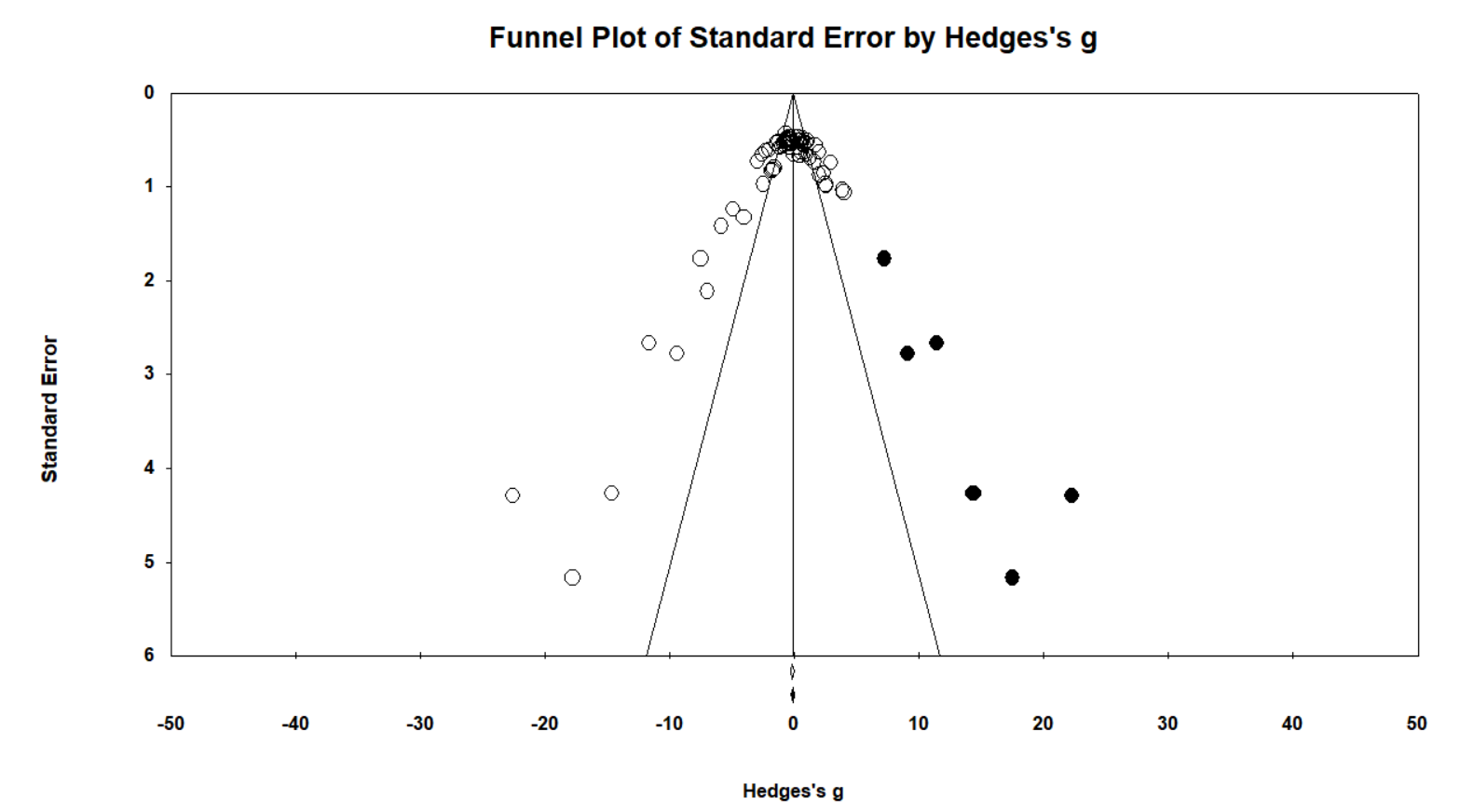
**

**Meta-regression of time from insulin administration in rats**

**
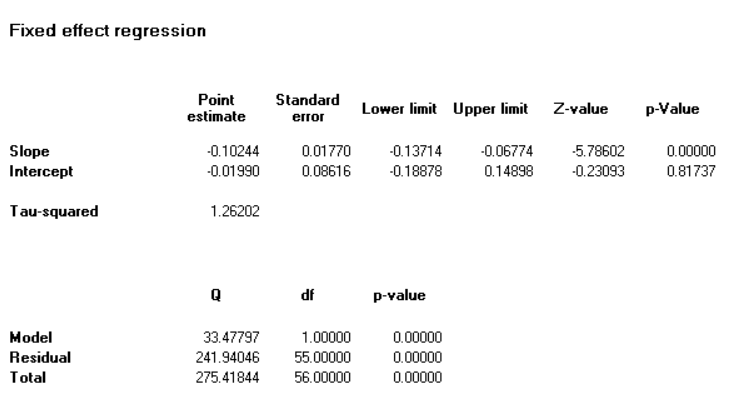
**

**
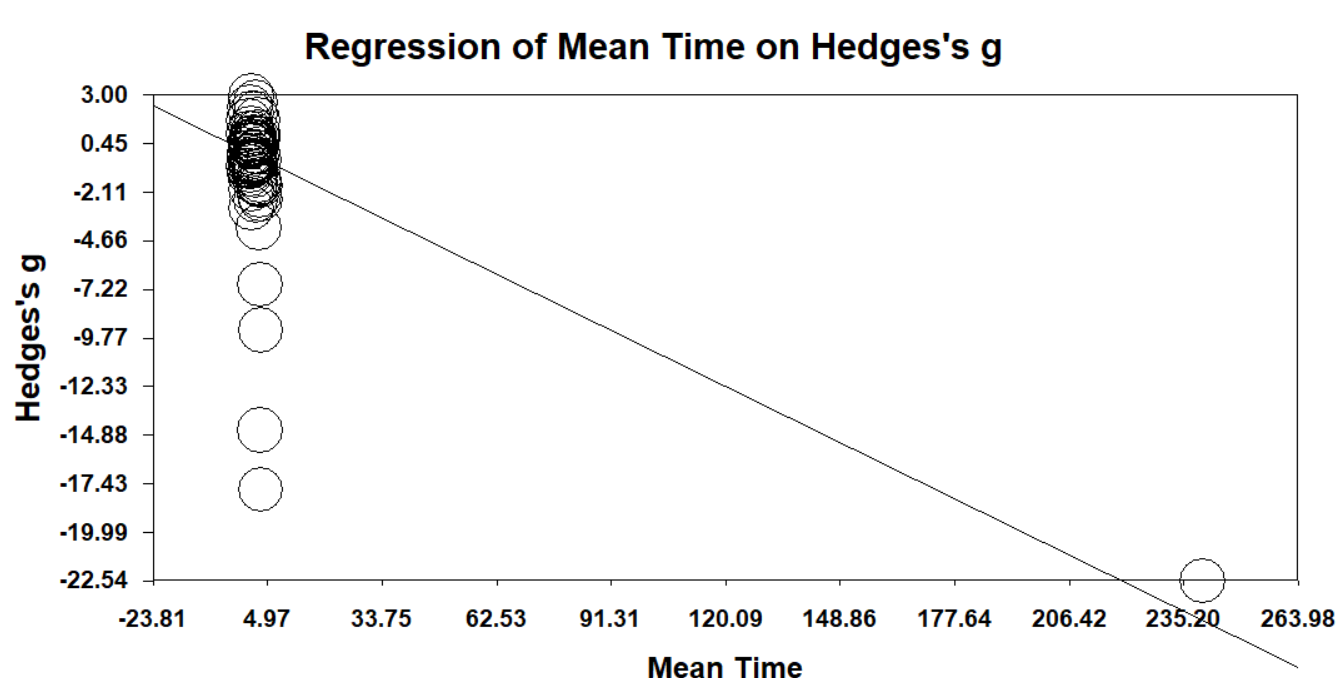
**

**Meta-regression of blood glucose ratio (cases/controls) in Wistar rats**

**
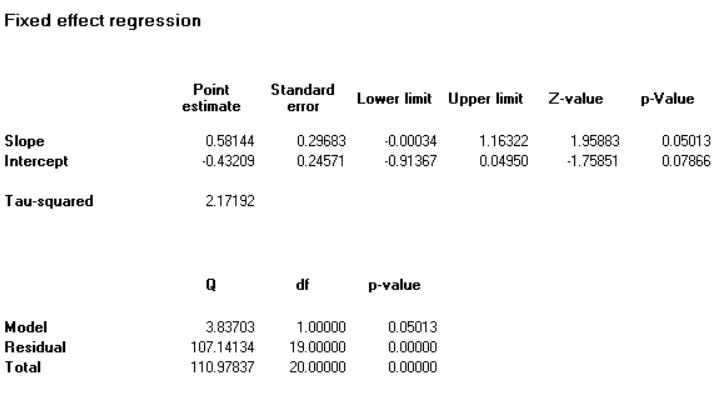
**

**
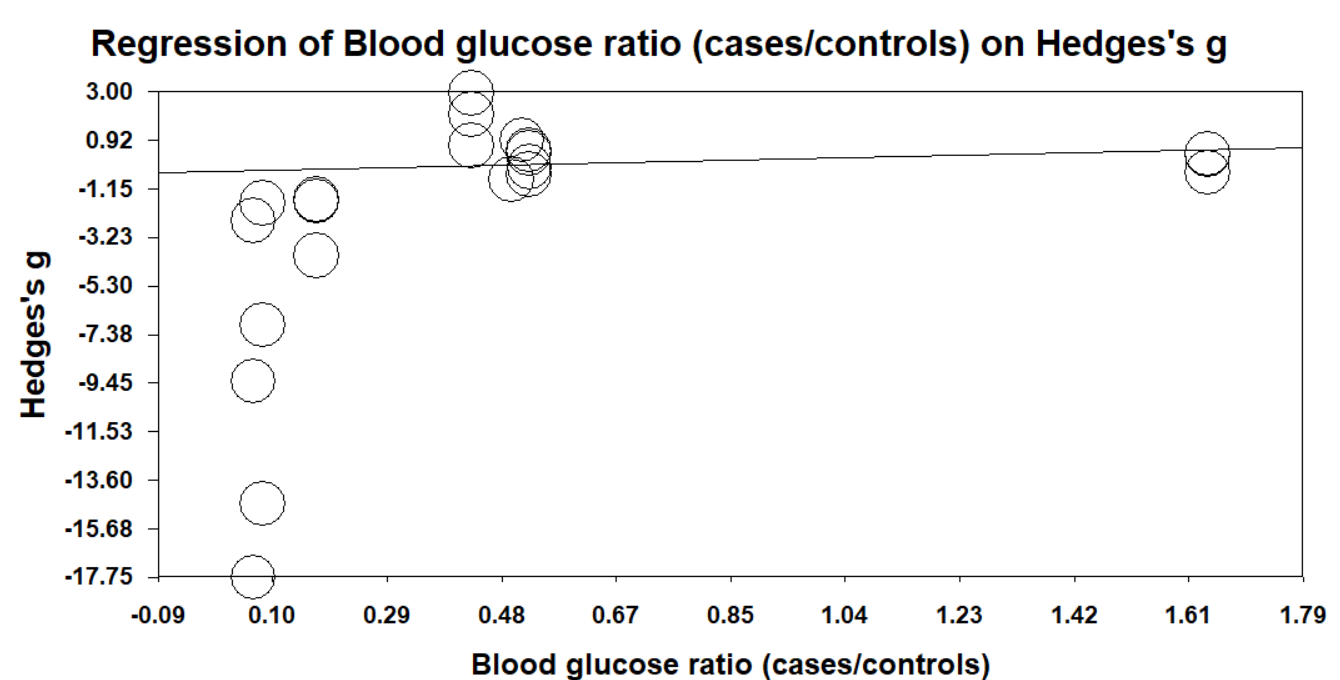
**

**Meta-regression of blood glucose ratio (cases/controls) in Sprague-Dawley rats**

**
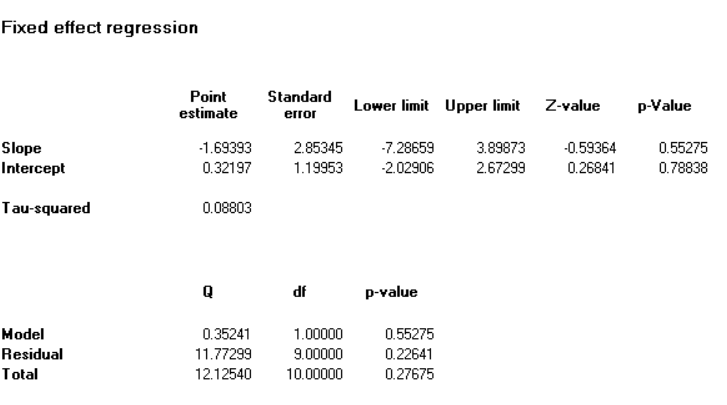
**

**
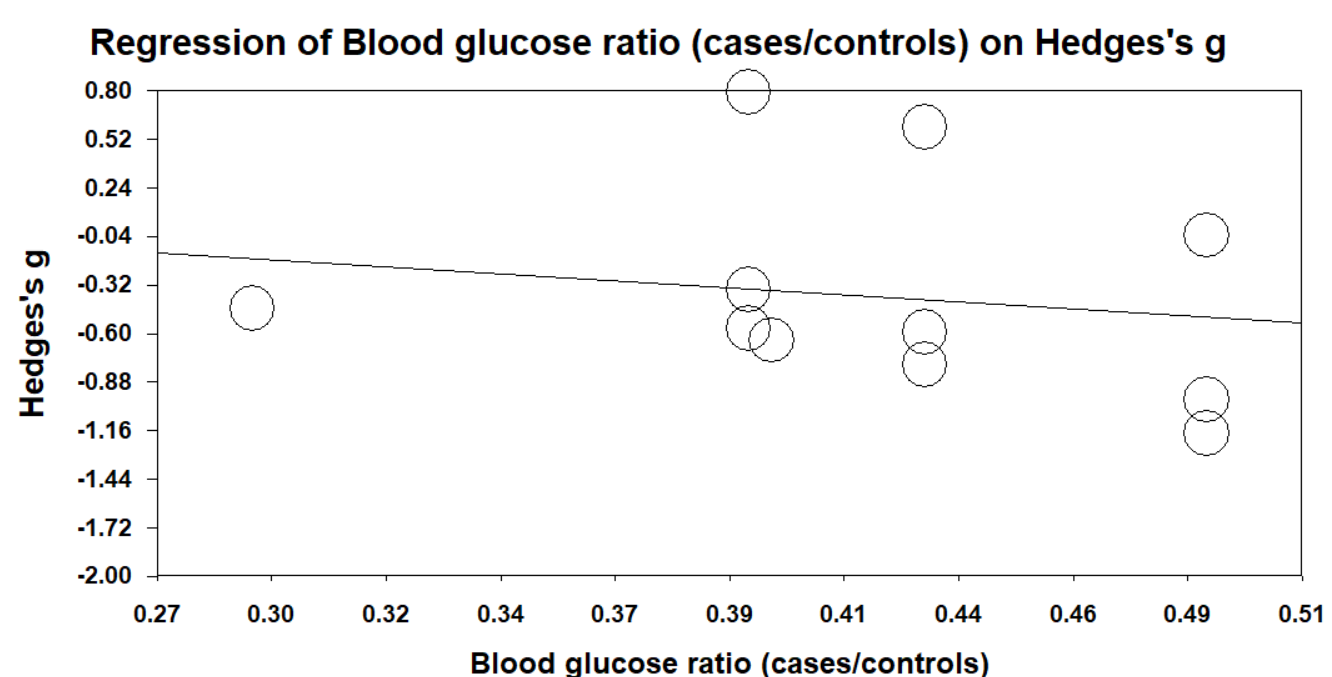
**

**Meta-regression of Age (weeks) in Wistar rats**

**
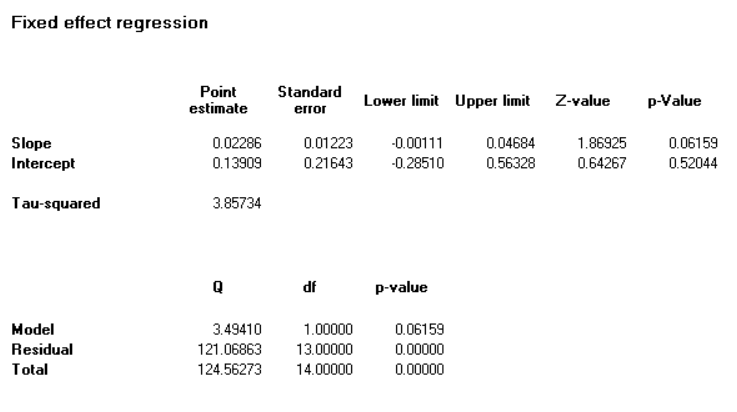
**

**
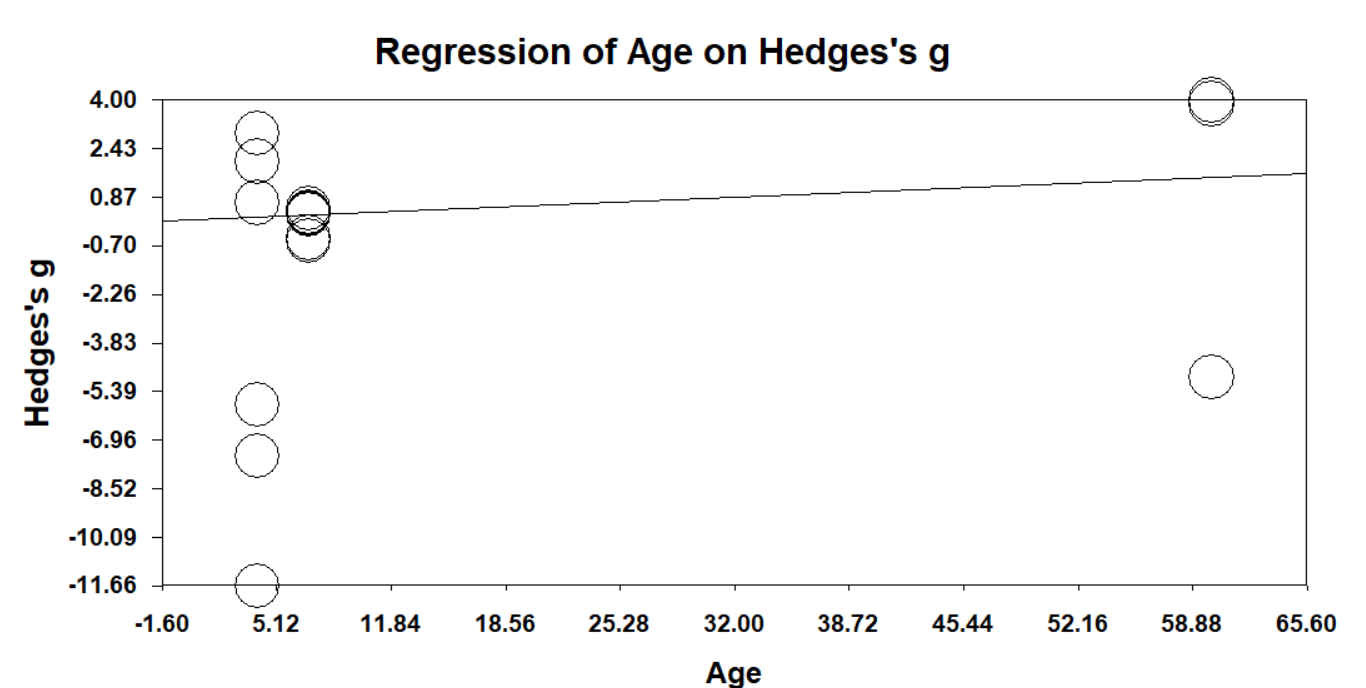
**

**Supplementary Figure n.8 – Dopamine D1 Receptor mRNA in hyperinsulinemia, main and subgroup analysis**

**
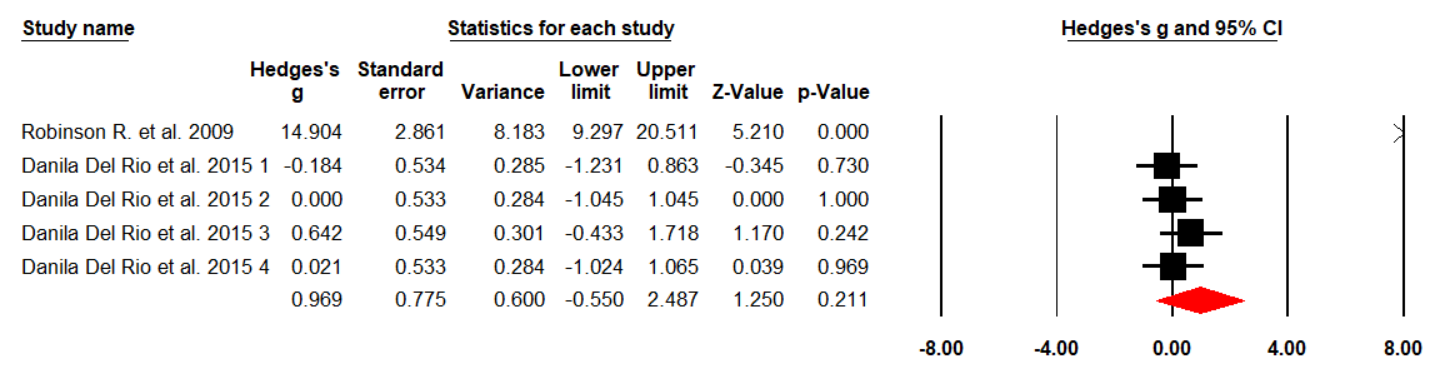
**

**
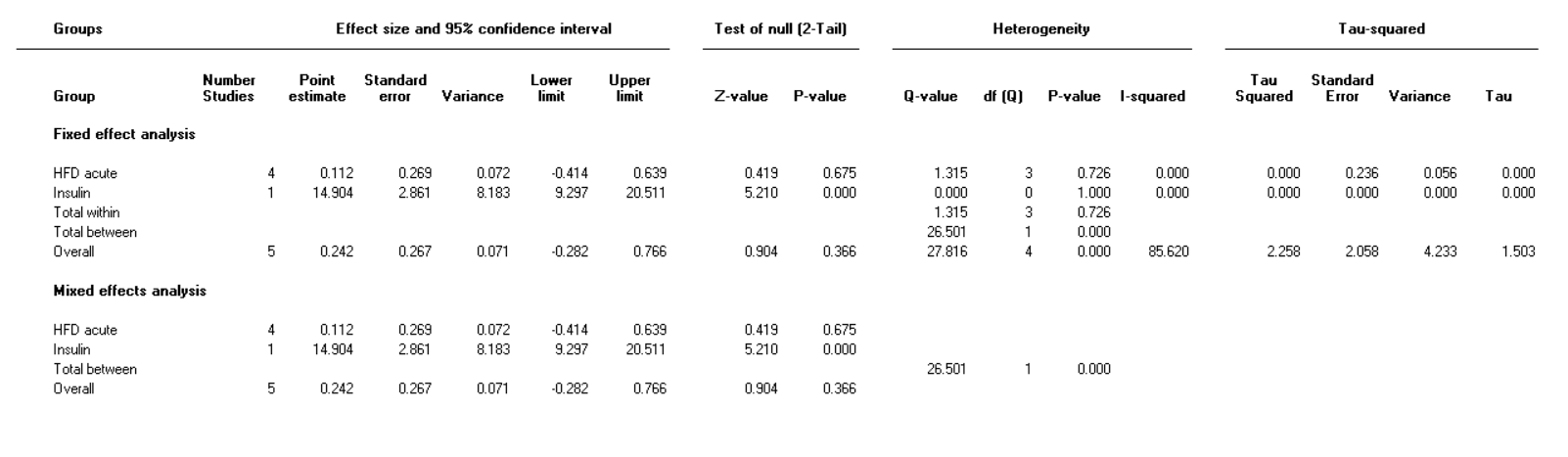
**

**
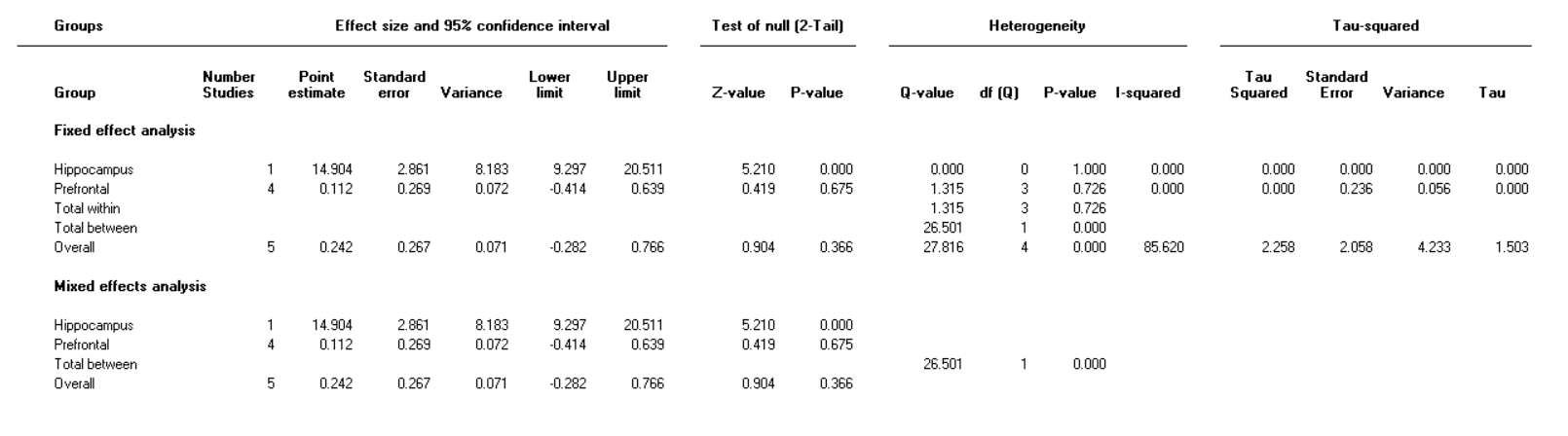
**

**Supplementary Figure n.9 -** **Dopamine D2 Receptor mRNA in hyperinsulinemia, main and subgroup analysis**

**
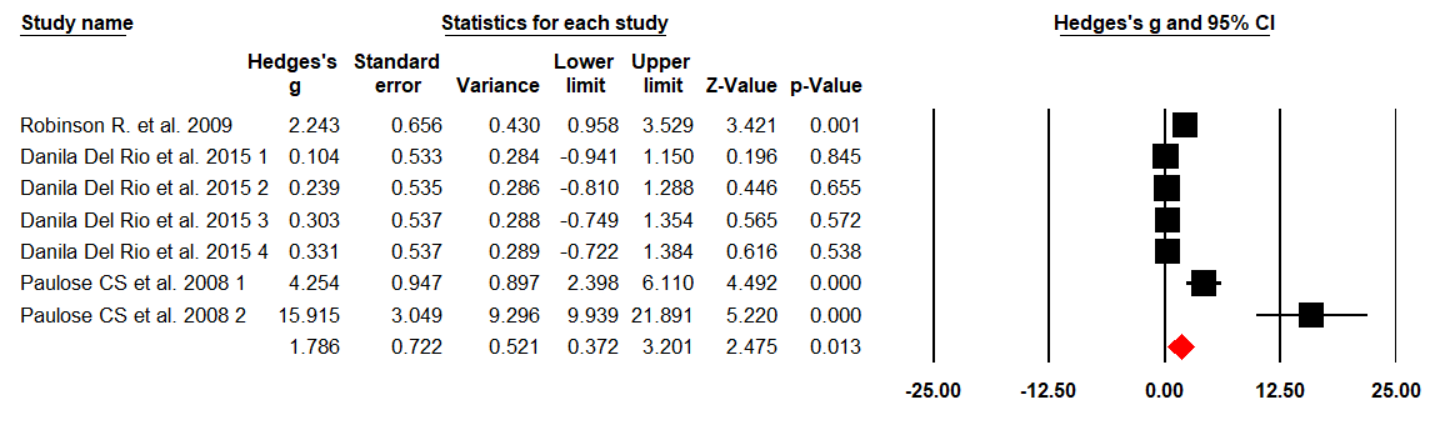
**

**
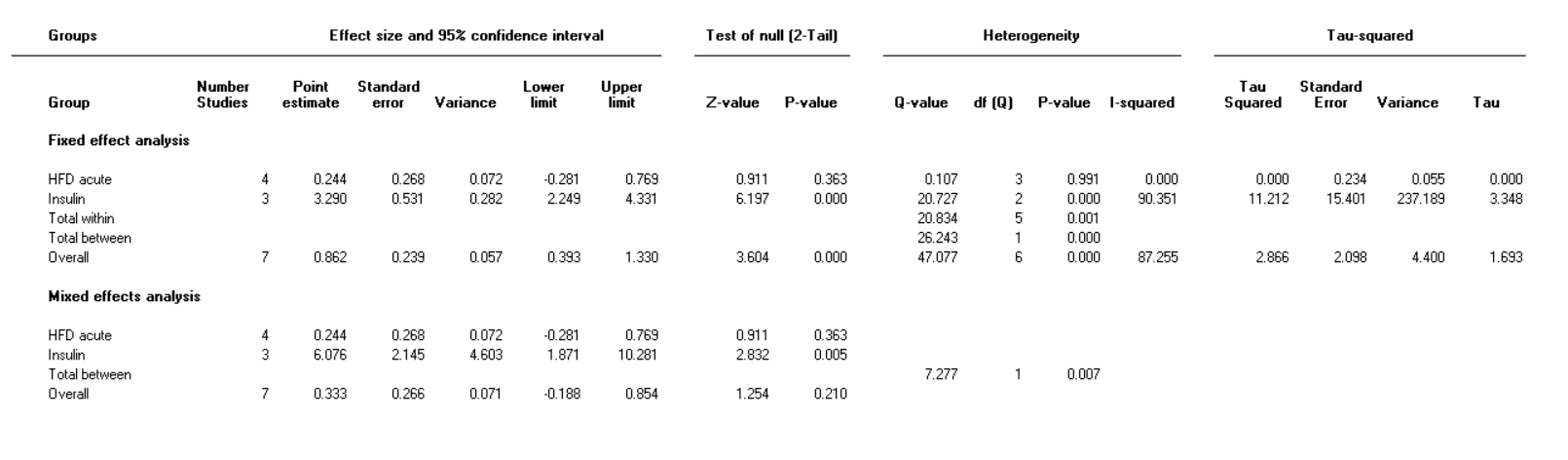
**

**
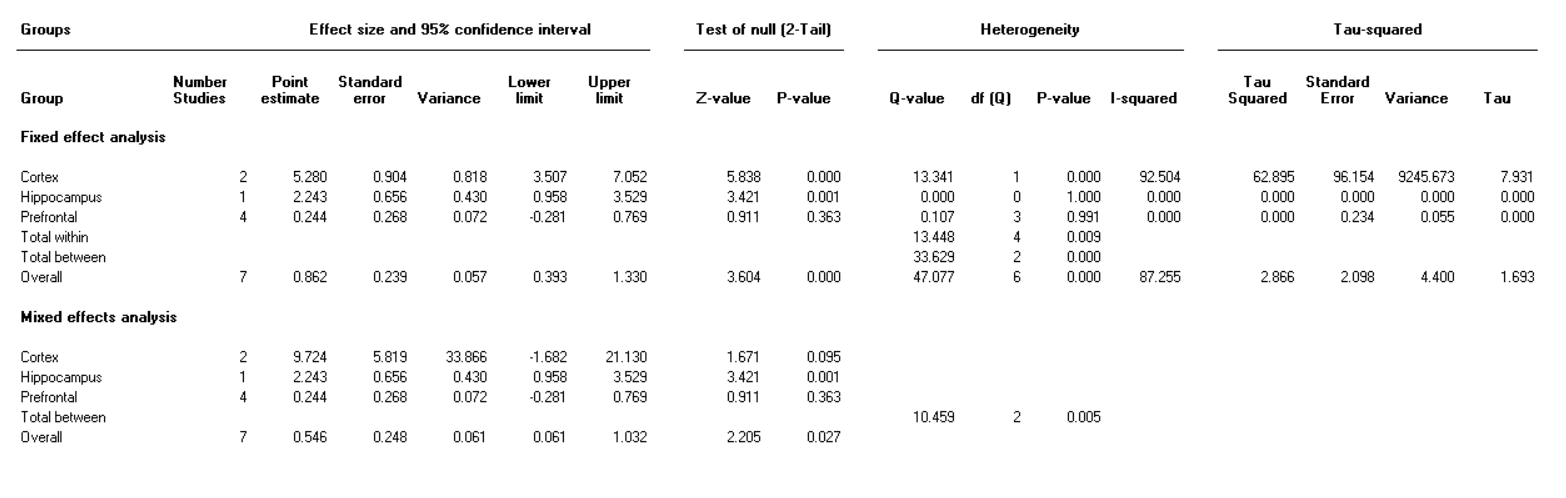
**

**Supplementary Figure n.10 - Dopamine Receptor sensitivity in hyperinsulinemia, main analysis**

**
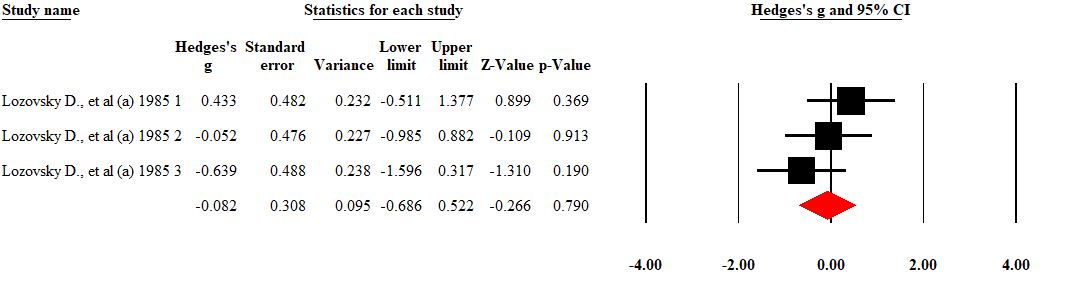
**

**
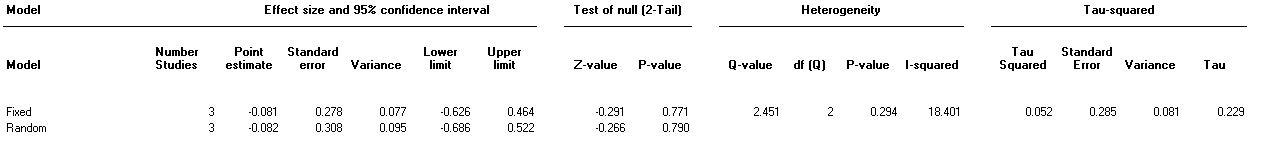
**

**Supplementary Figure n.11 - HVA concentration in hyperinsulinemia, main and subgroup analysis, publication bias, and meta-regression**

**
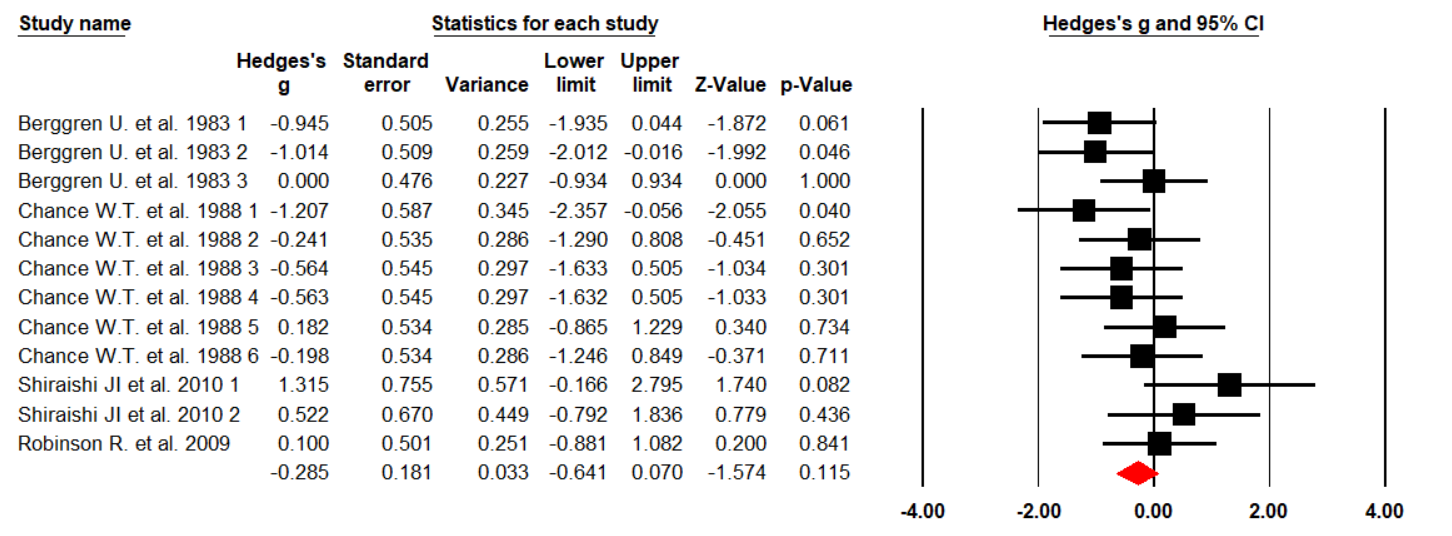
**

**
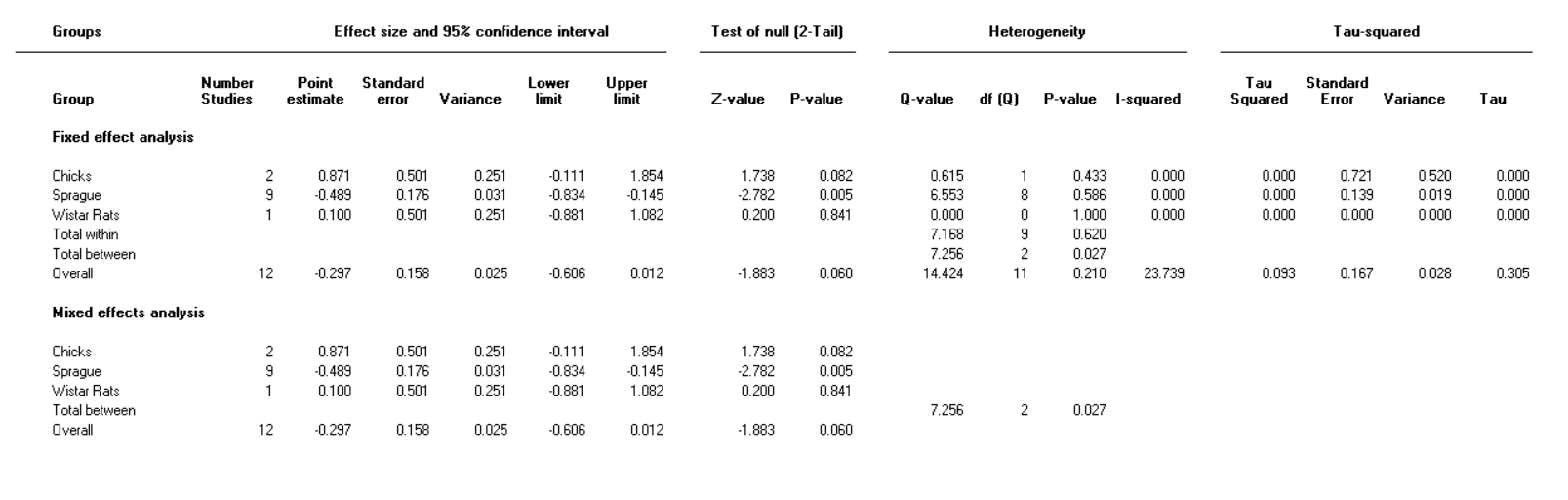
**

**
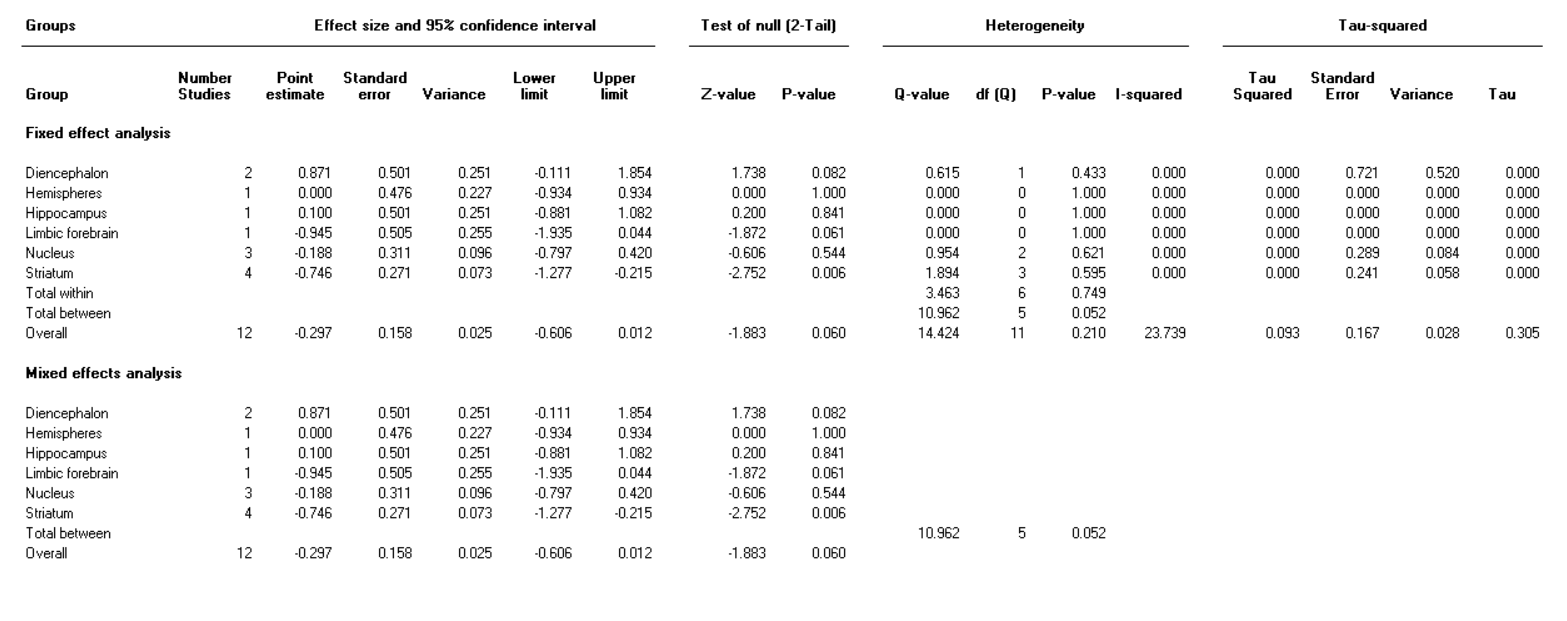
**

**
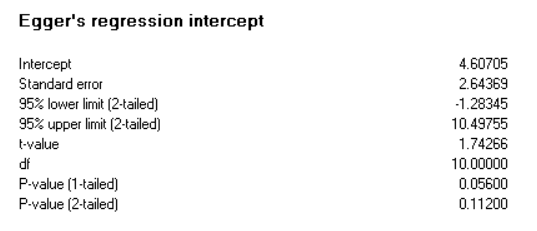
**

**
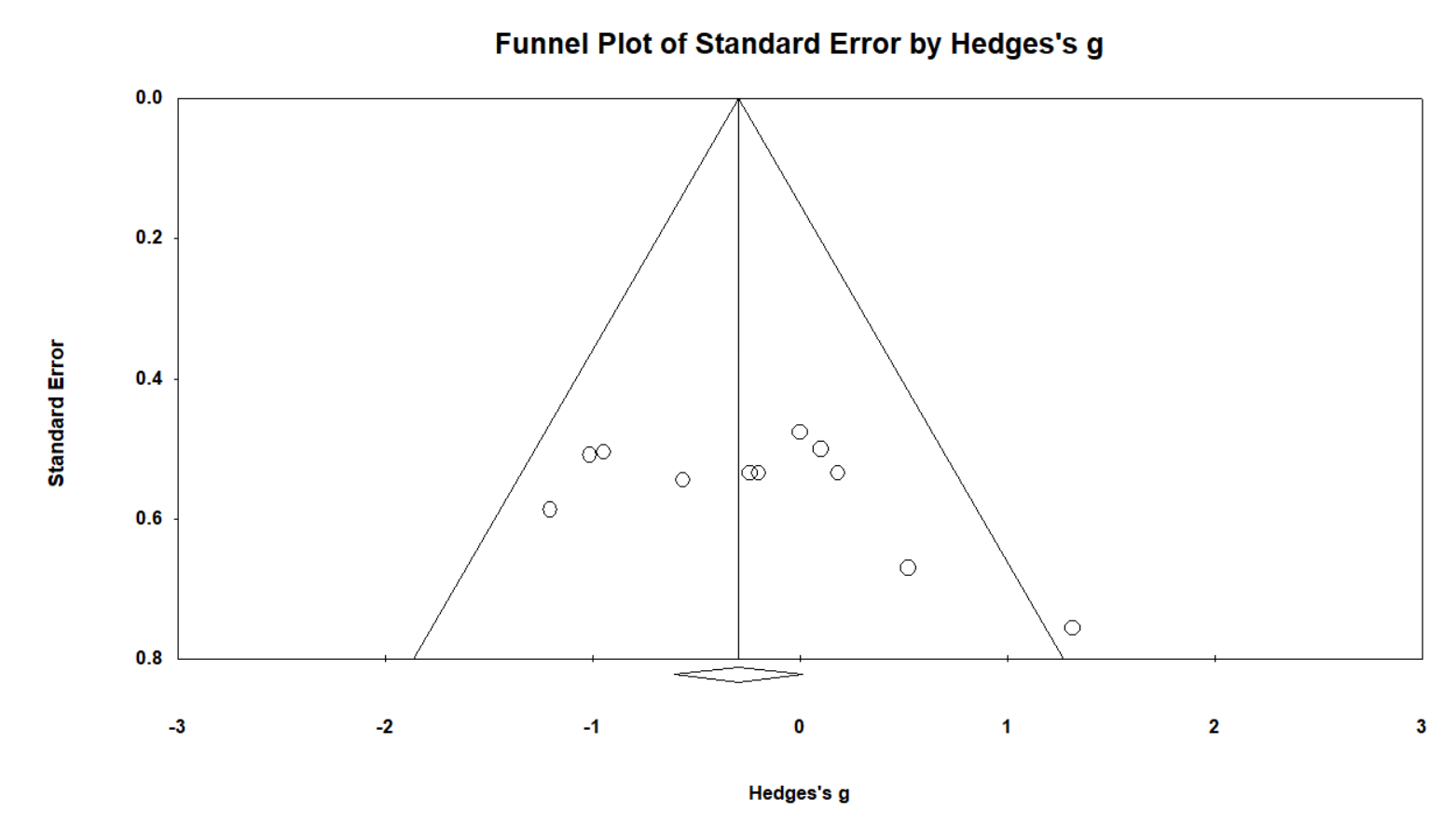
**

**Meta-regression of time from insulin injection in rats**

**
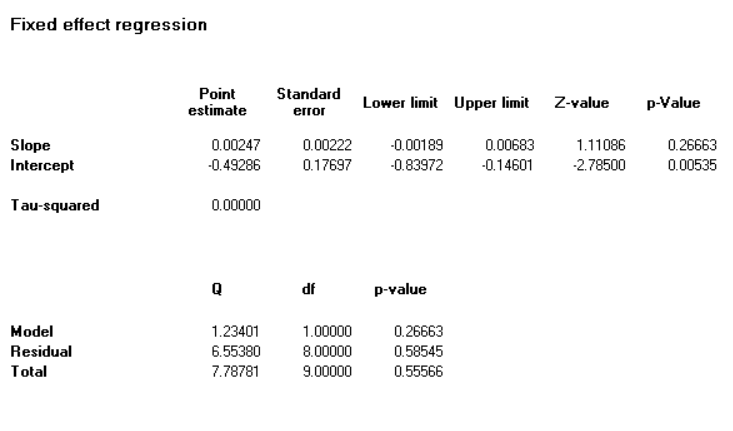

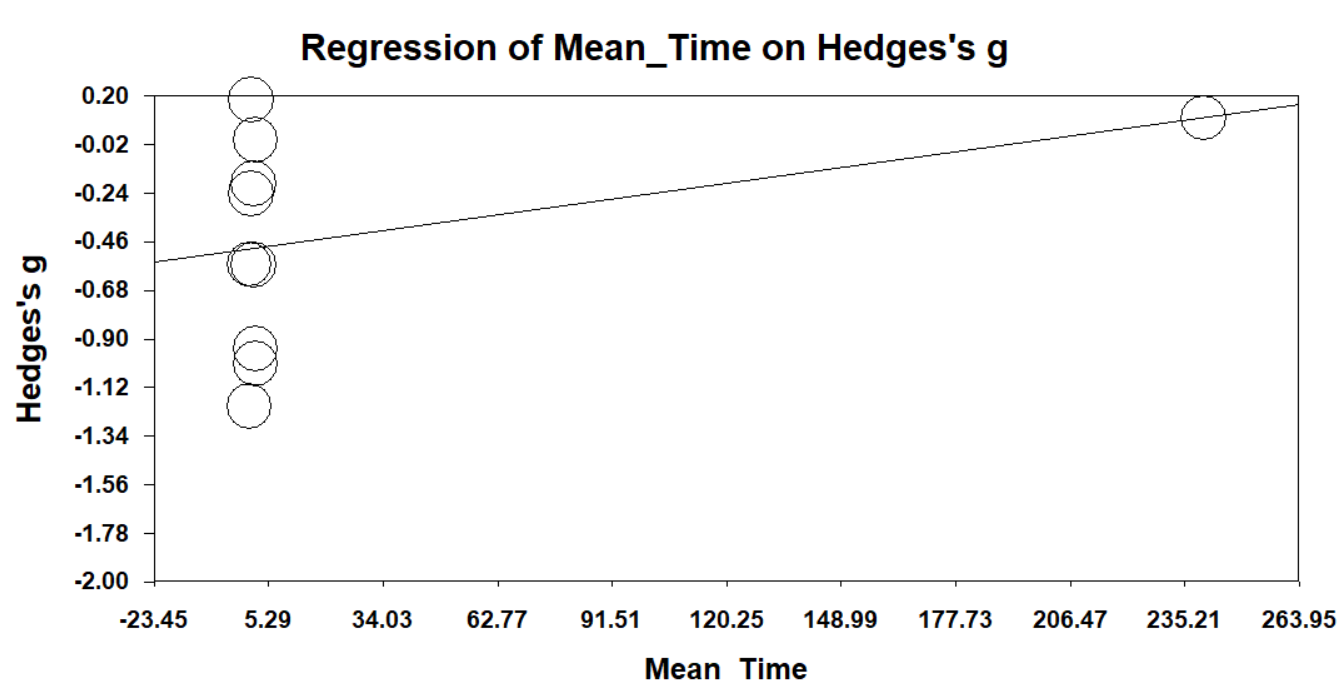
**

**Supplementary Figure n.12 - MAO activity in hyperinsulinemia, main and subgroup analyses**

**
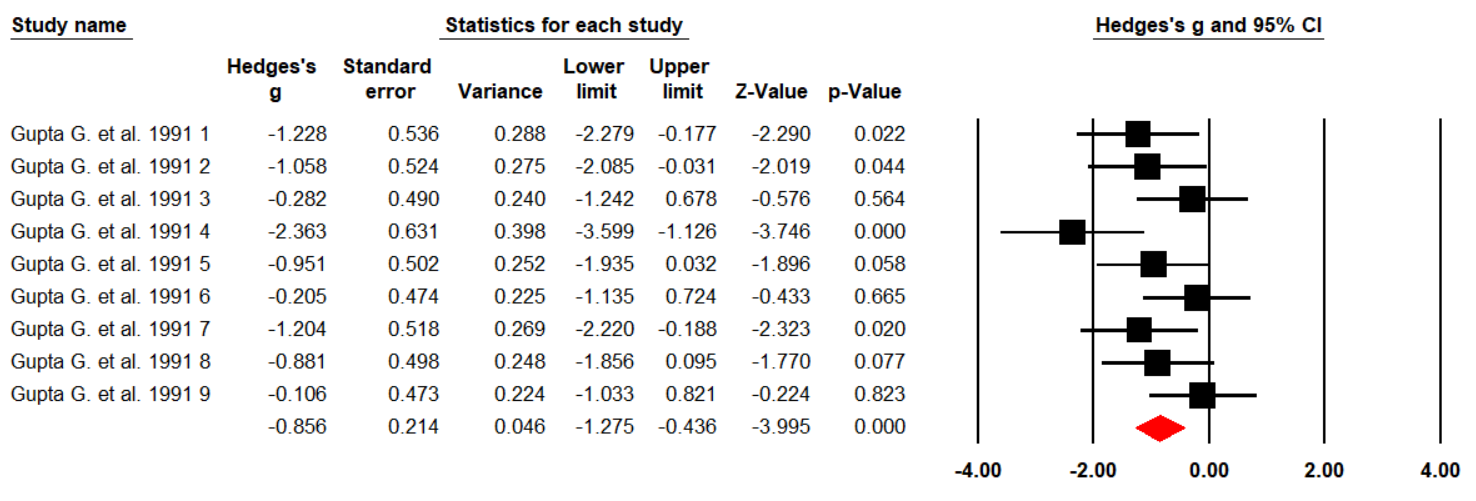
**

**
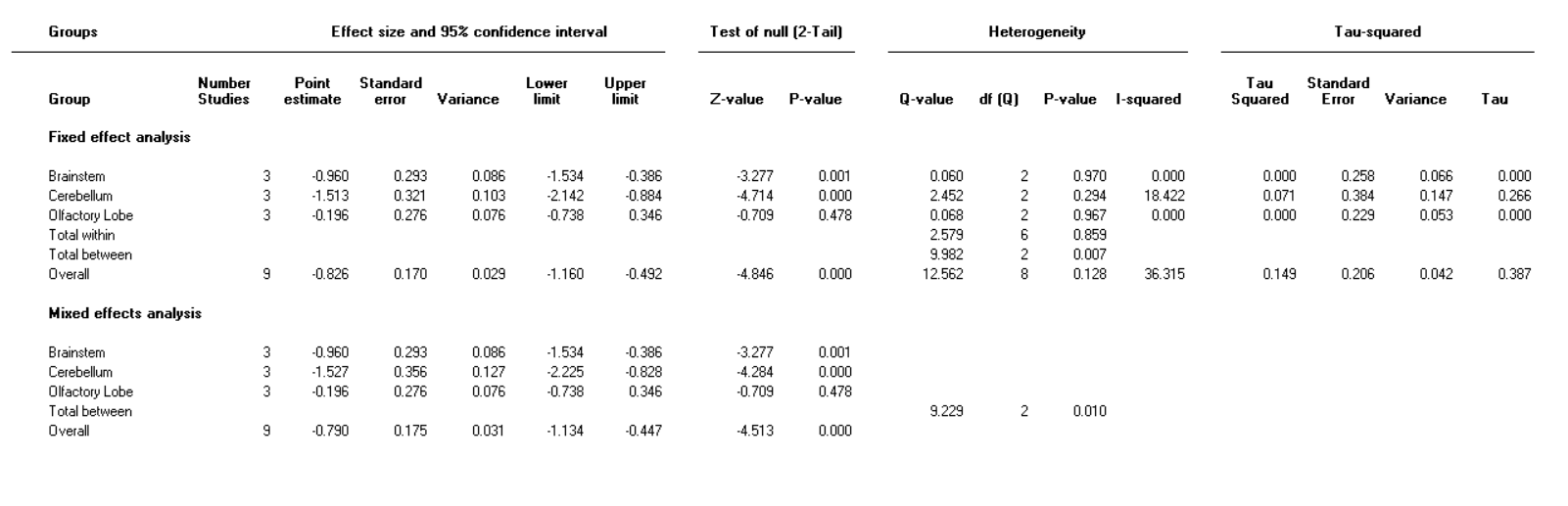
**

**Supplementary Figure n.13 - TH activity in hyperinsulinemia, main and subgroup analyses**

**
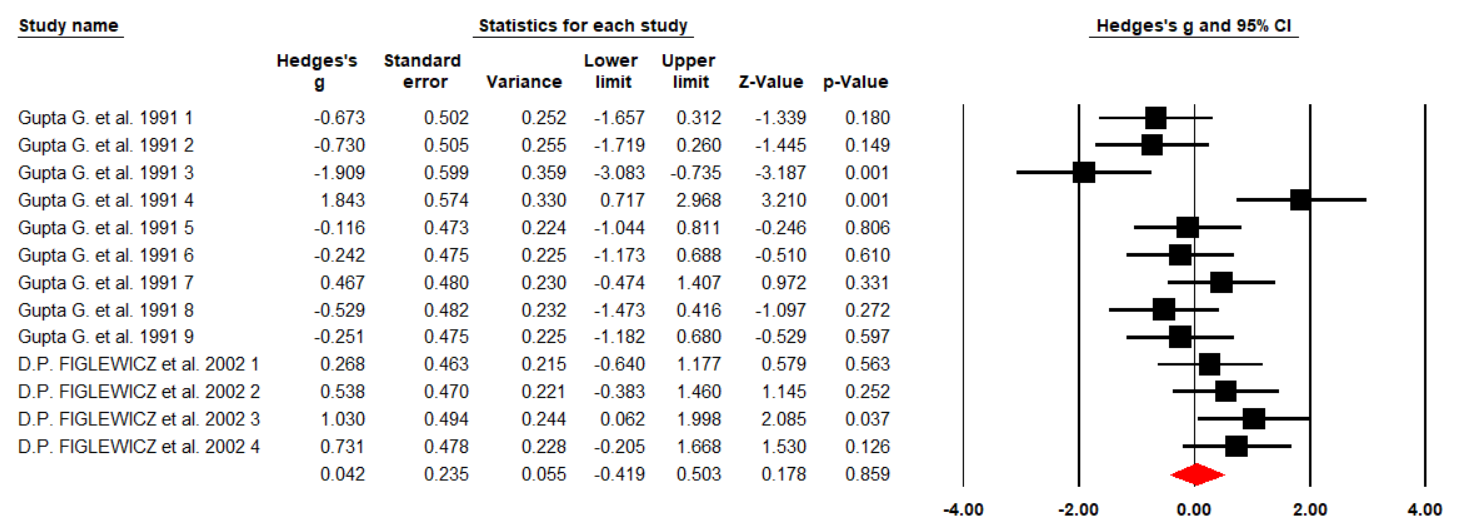
**

**
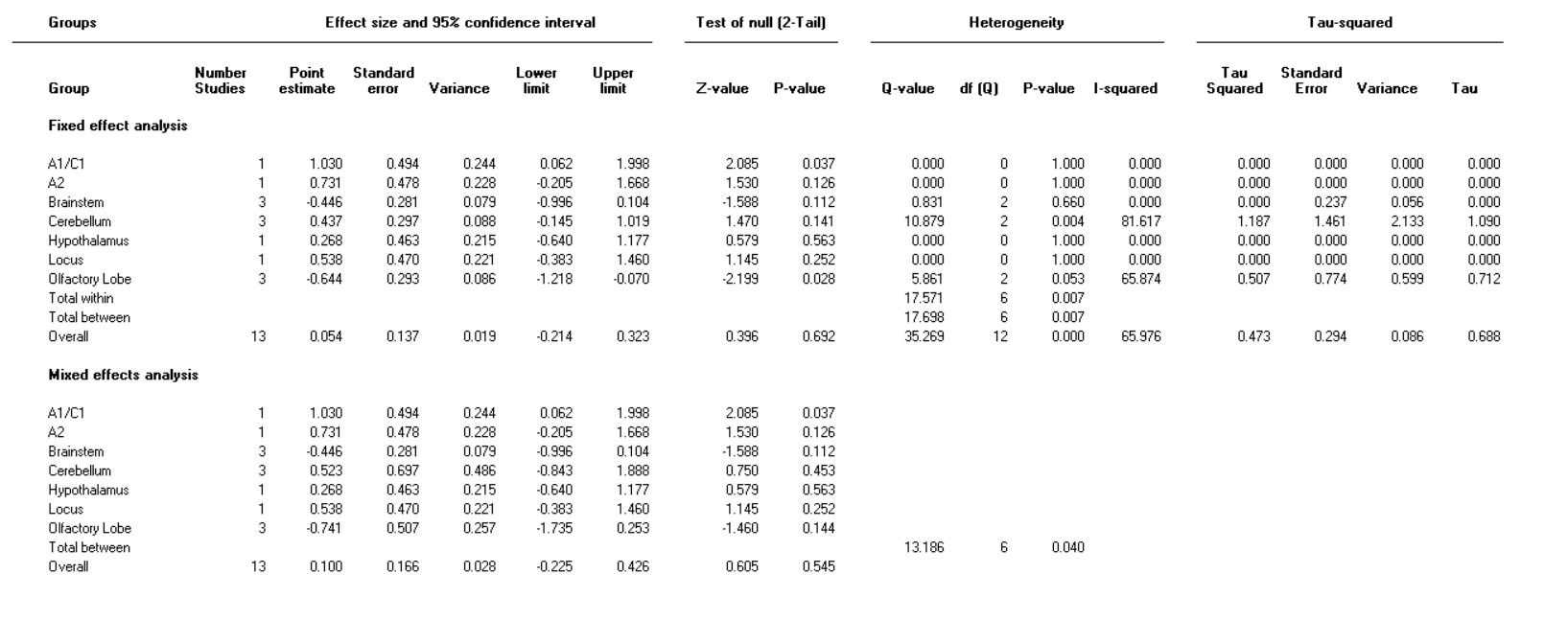
**

**
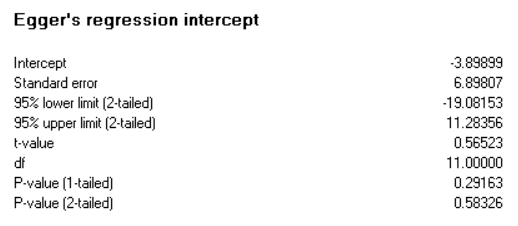
**

**
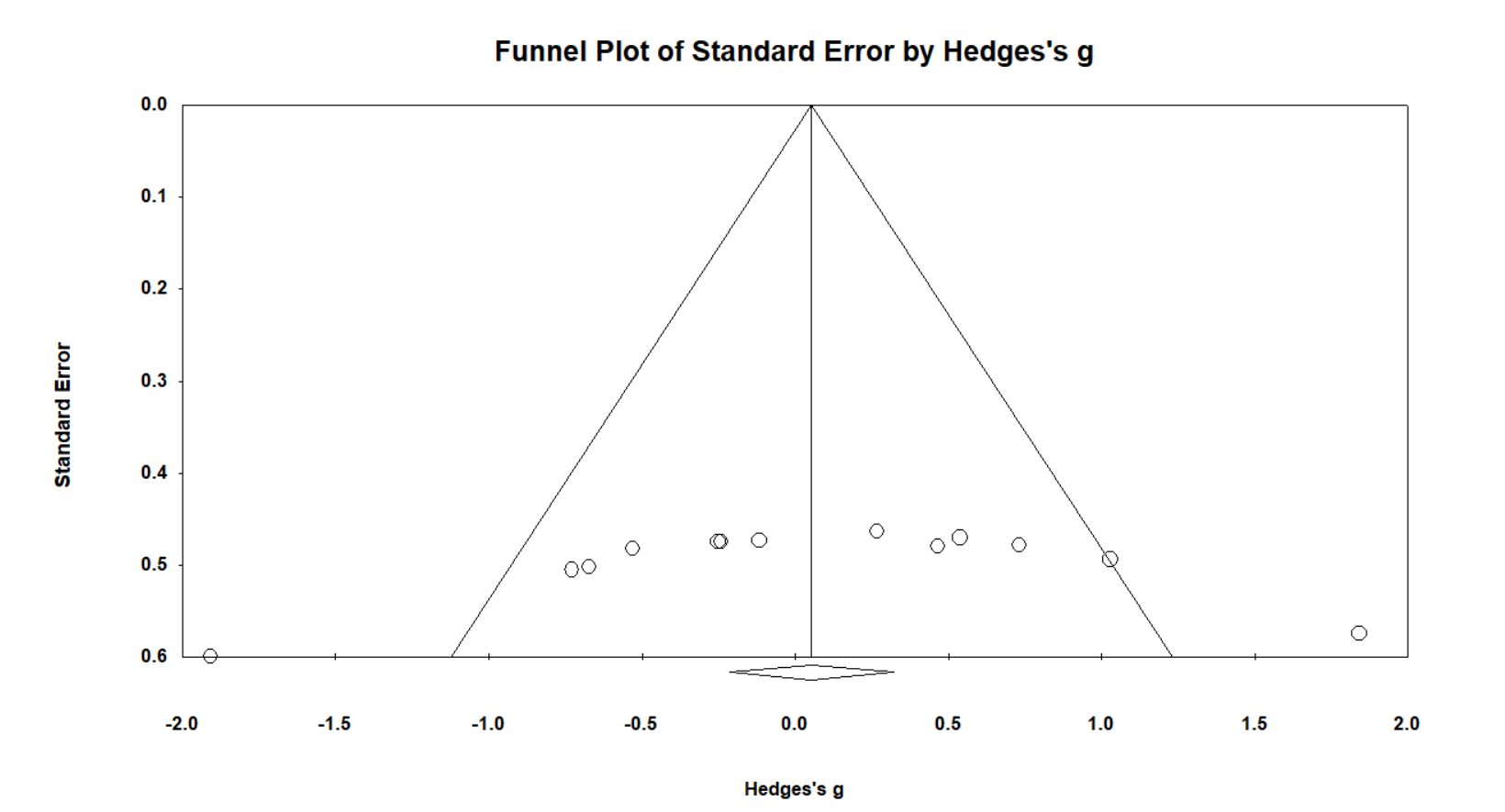
**

**Meta-regression of blood glucose ratio (cases/controls)**

**
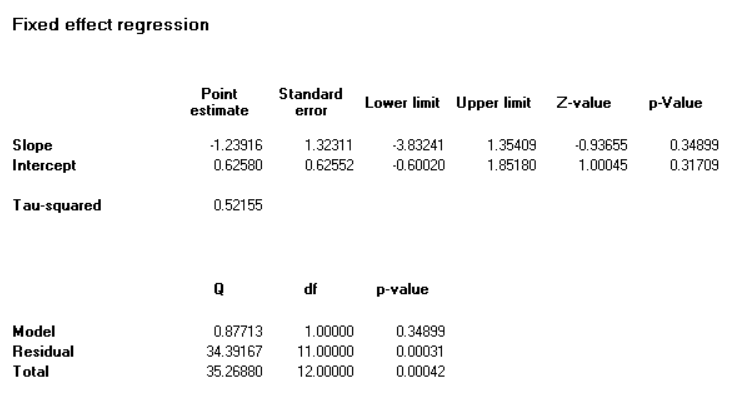
**

**
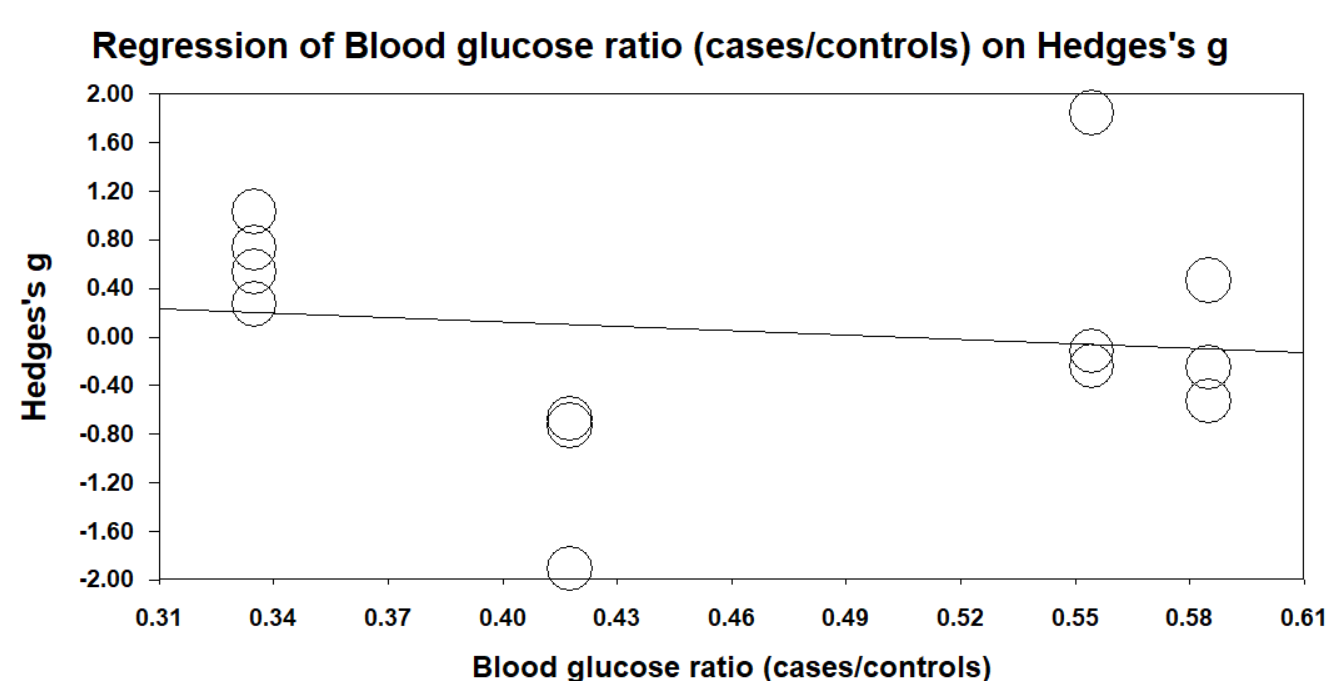
**

**Supplementary Figure n.14 - TH mRNA in hyperinsulinemia, main and subgroup analyses**

**
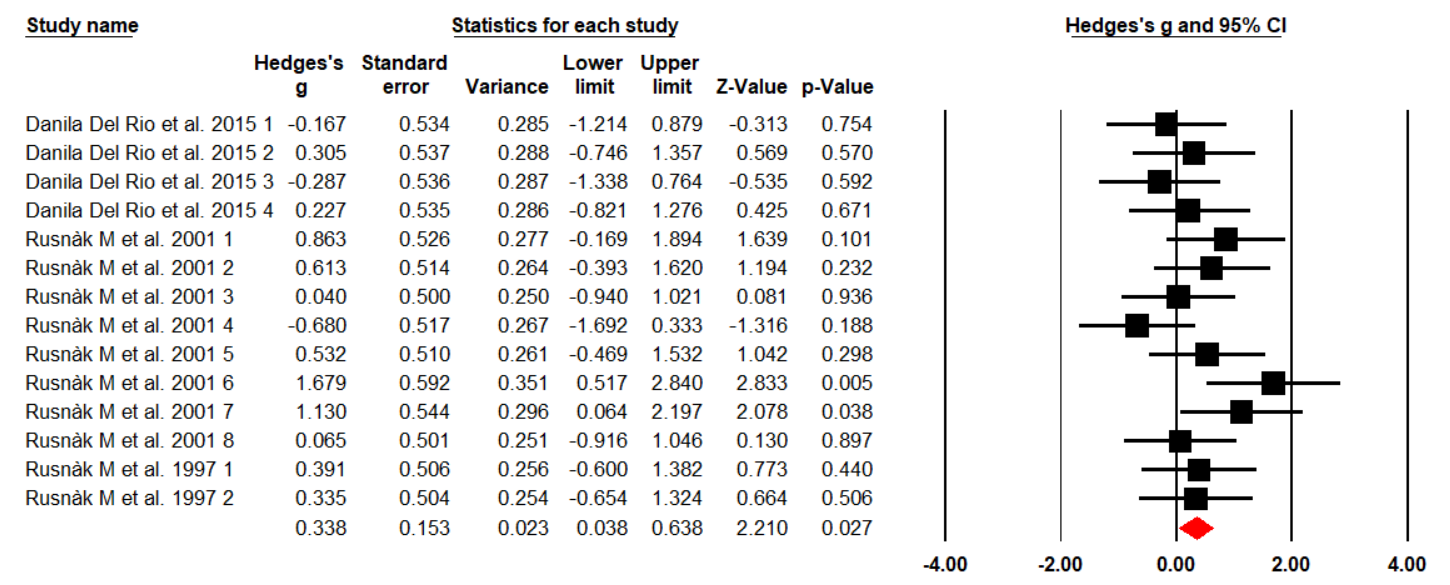
**

**
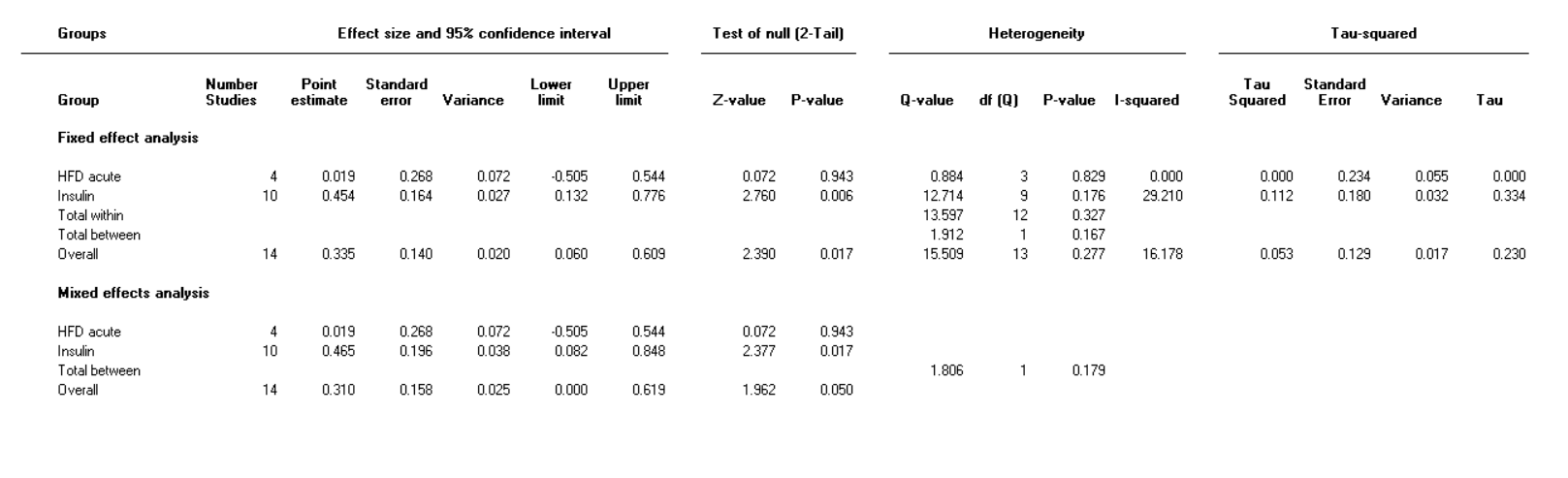
**

**
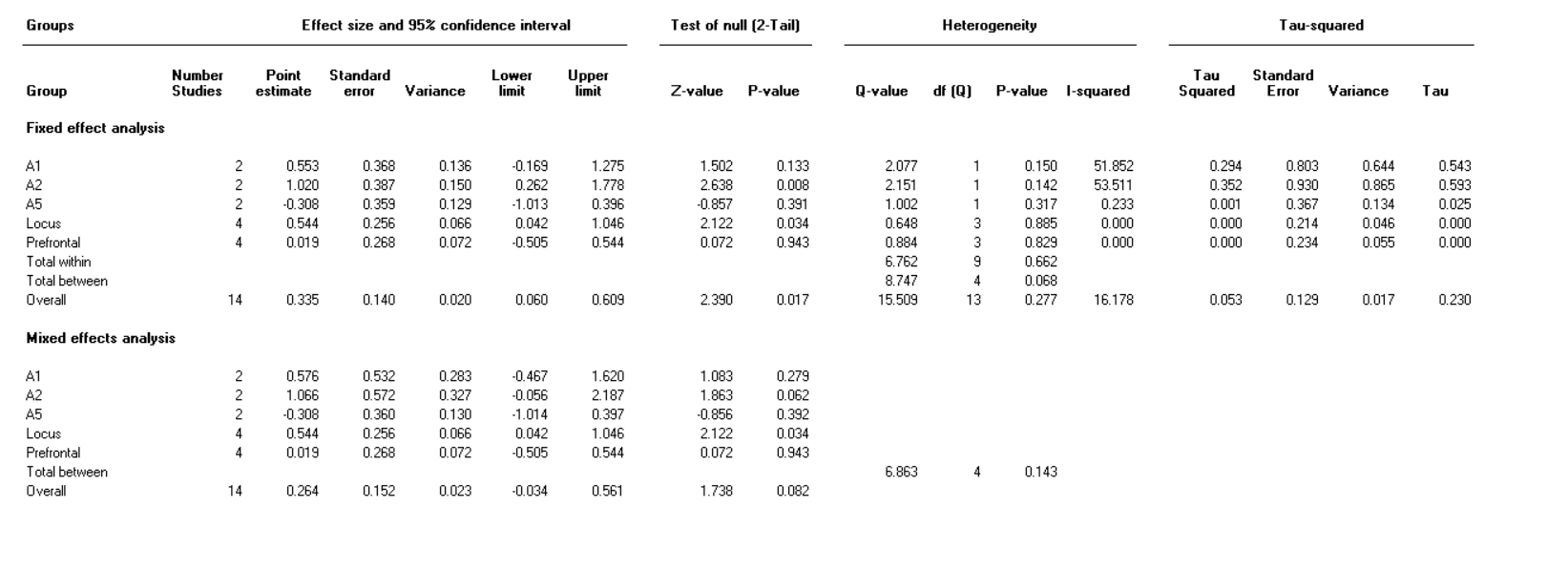
**

**
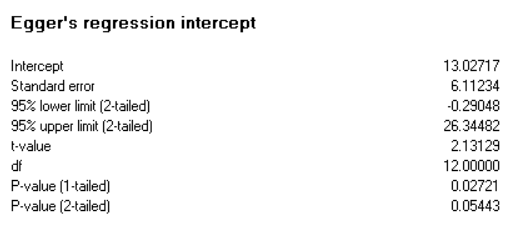
**

**
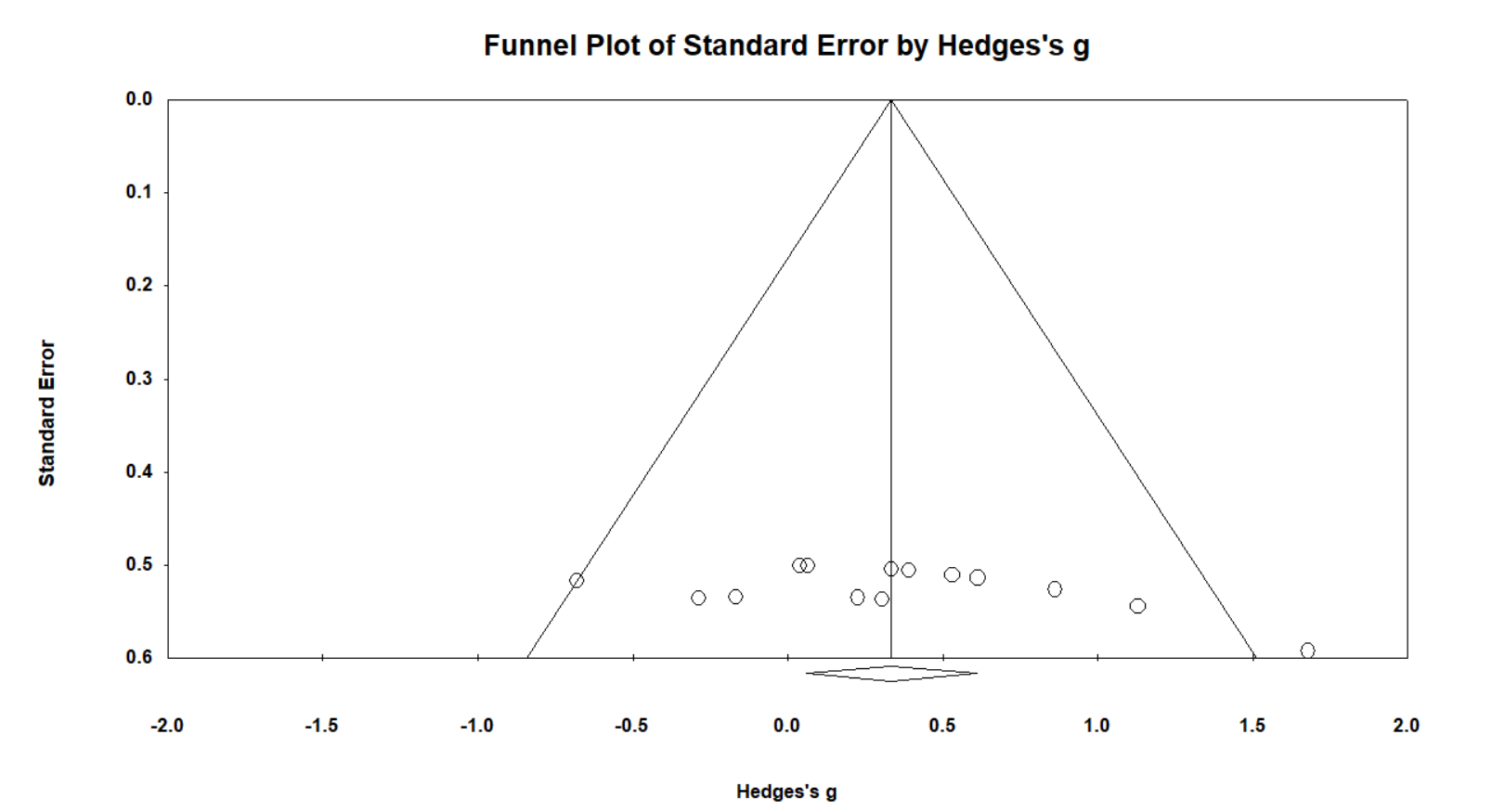
**

**Meta-regression of time from insulin administration**

**
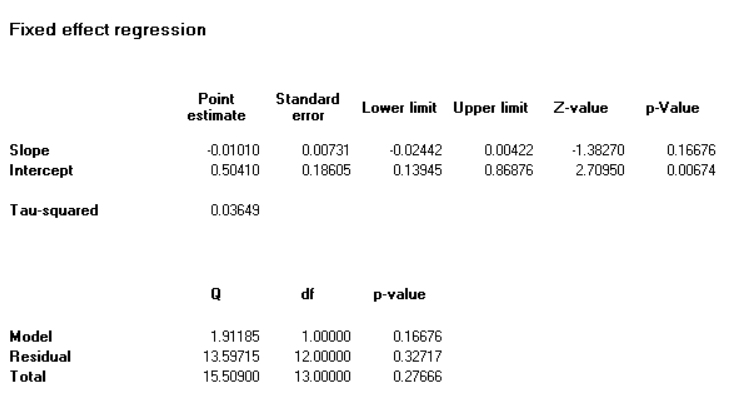

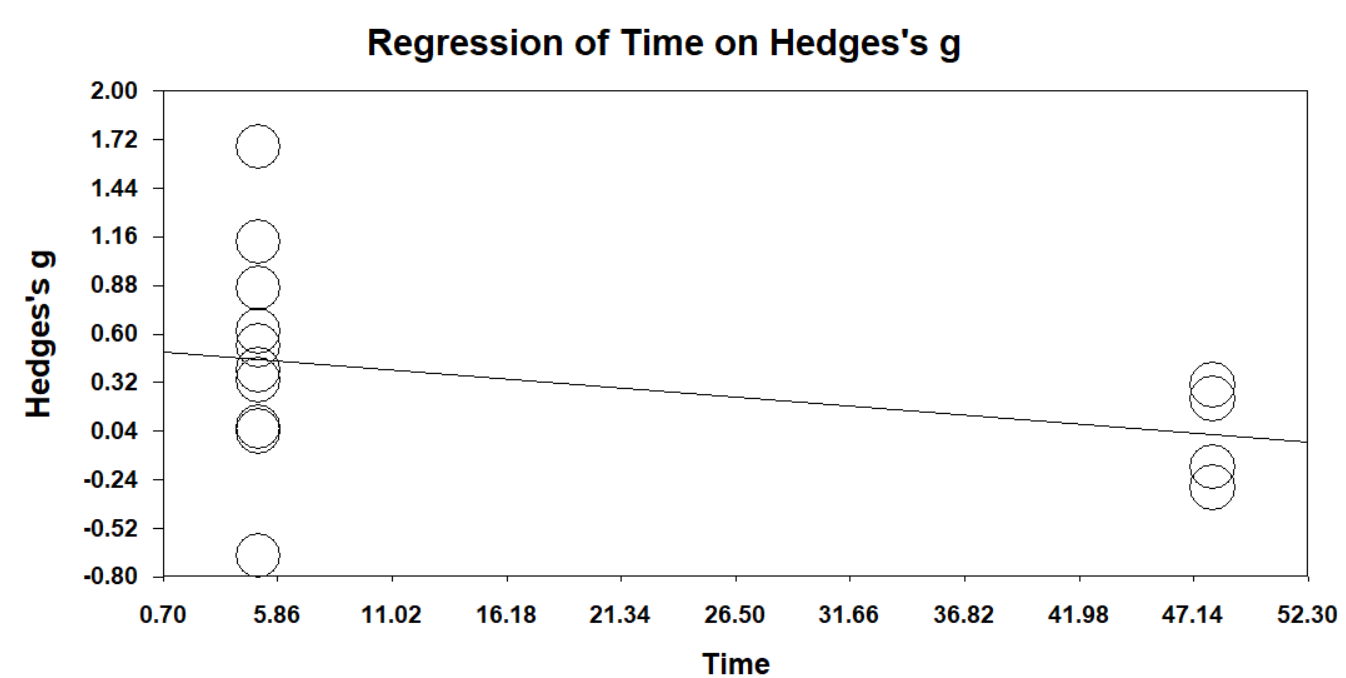
**

**Supplementary Figure n.15 - Tyrosine accumulation in hyperinsulinemia, main analysis**

**
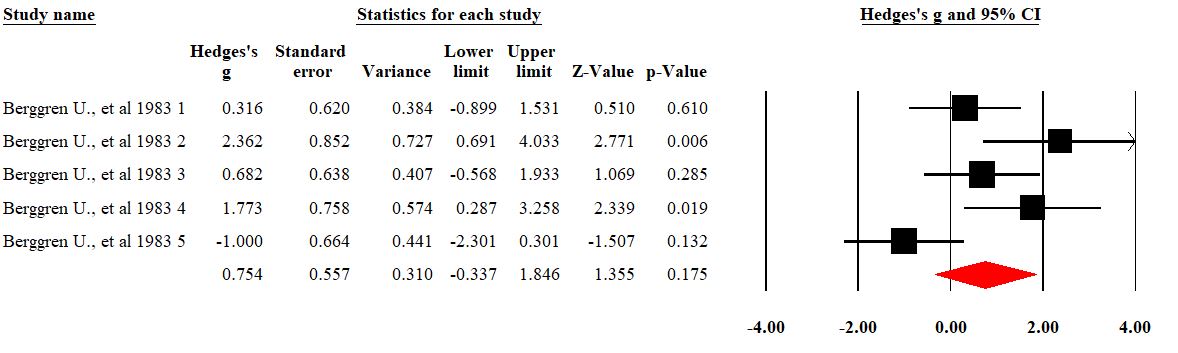
**

**
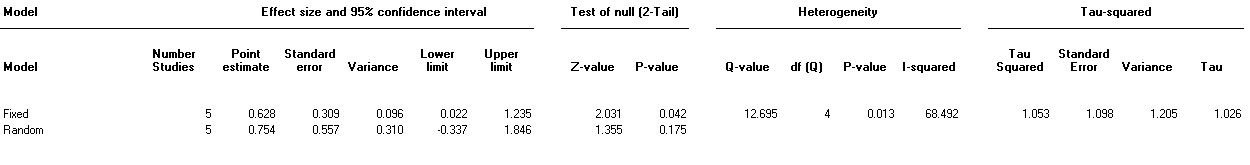
**

**Supplementary Figure n.16 - Tyrosine concentration in hyperinsulinemia, main and subgroup analysis, publication bias, and meta-regression**

**
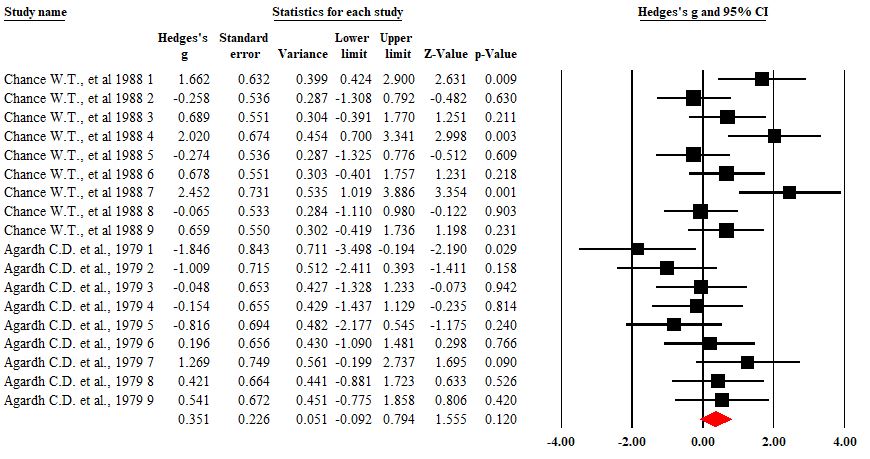
**

**
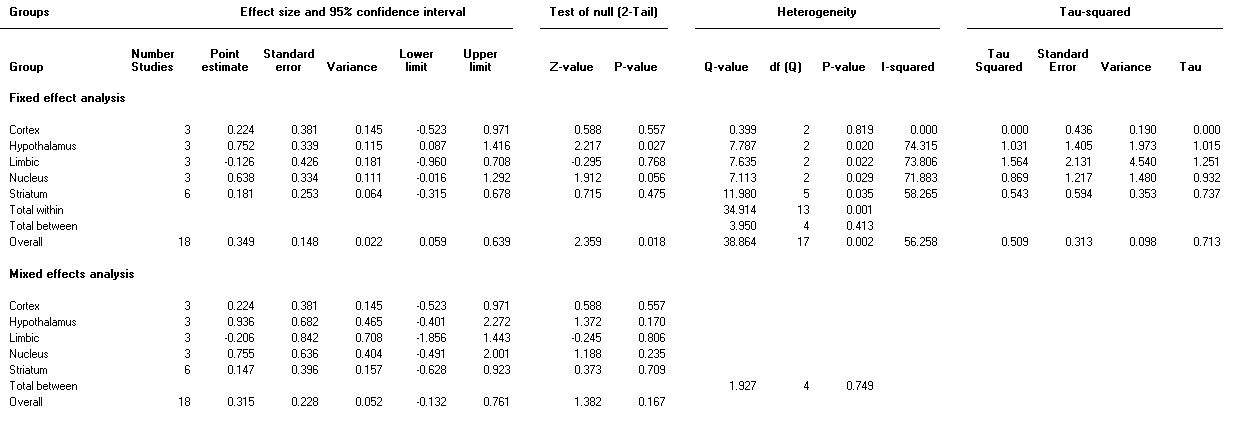
**


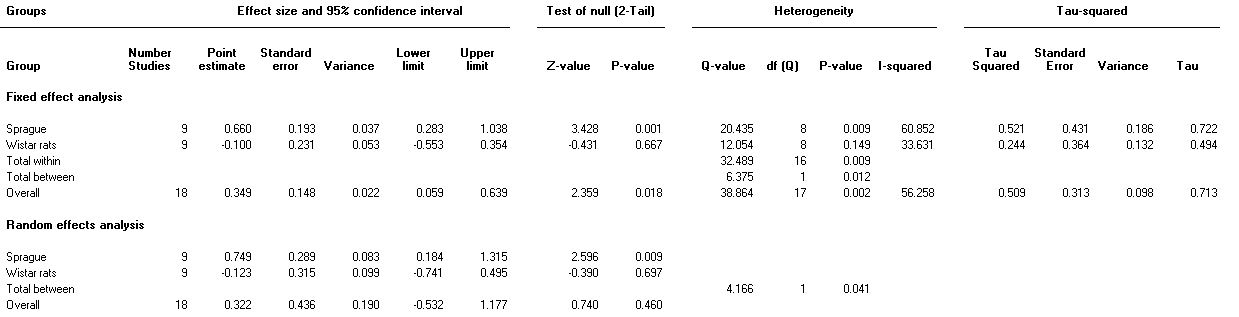


**
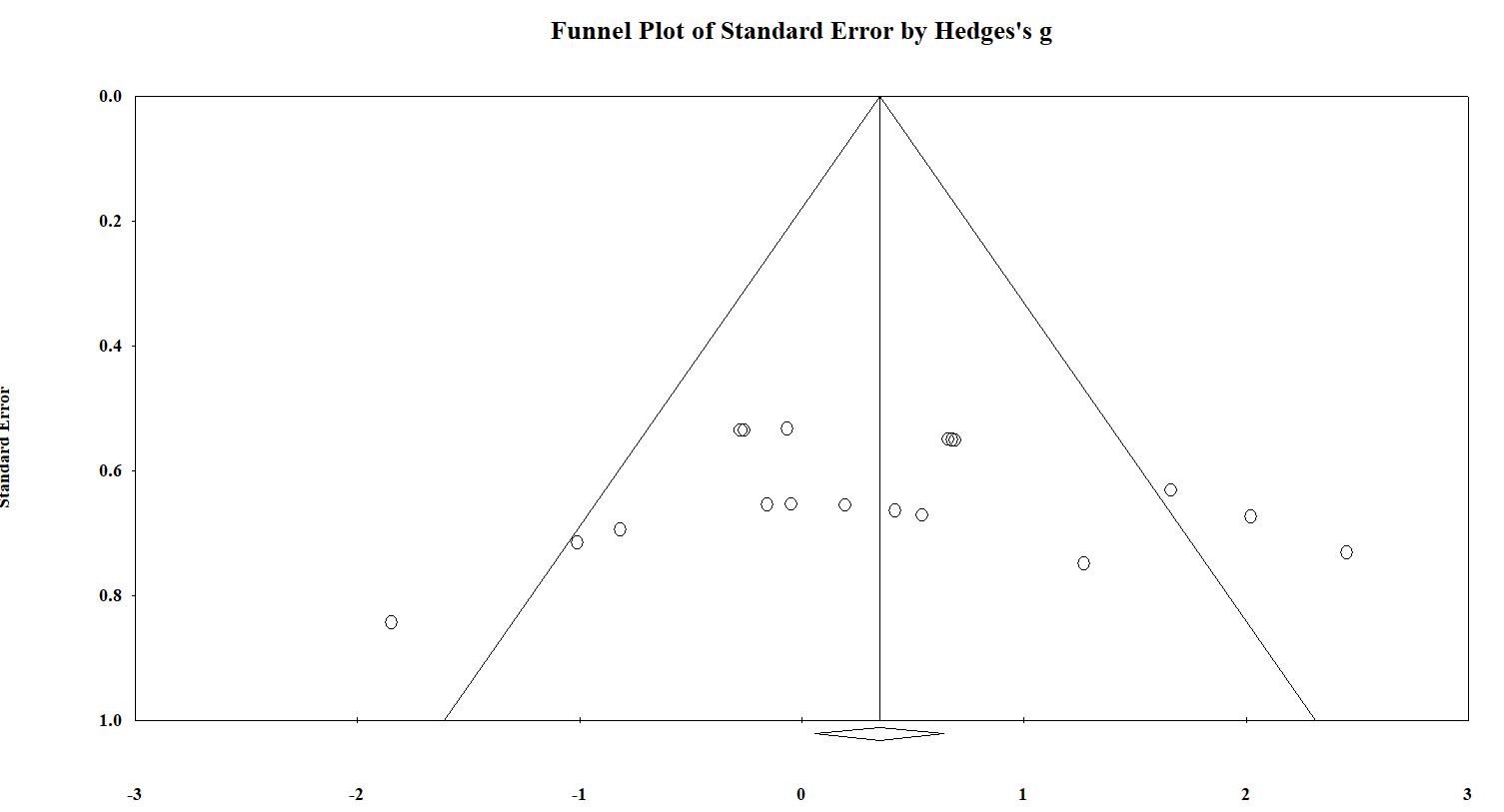
**

**Meta-regression of time from insulin injection**

**
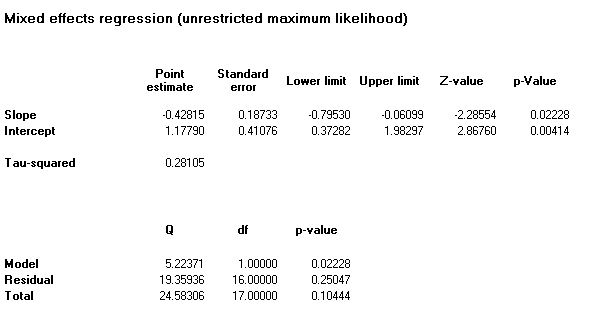
**

**
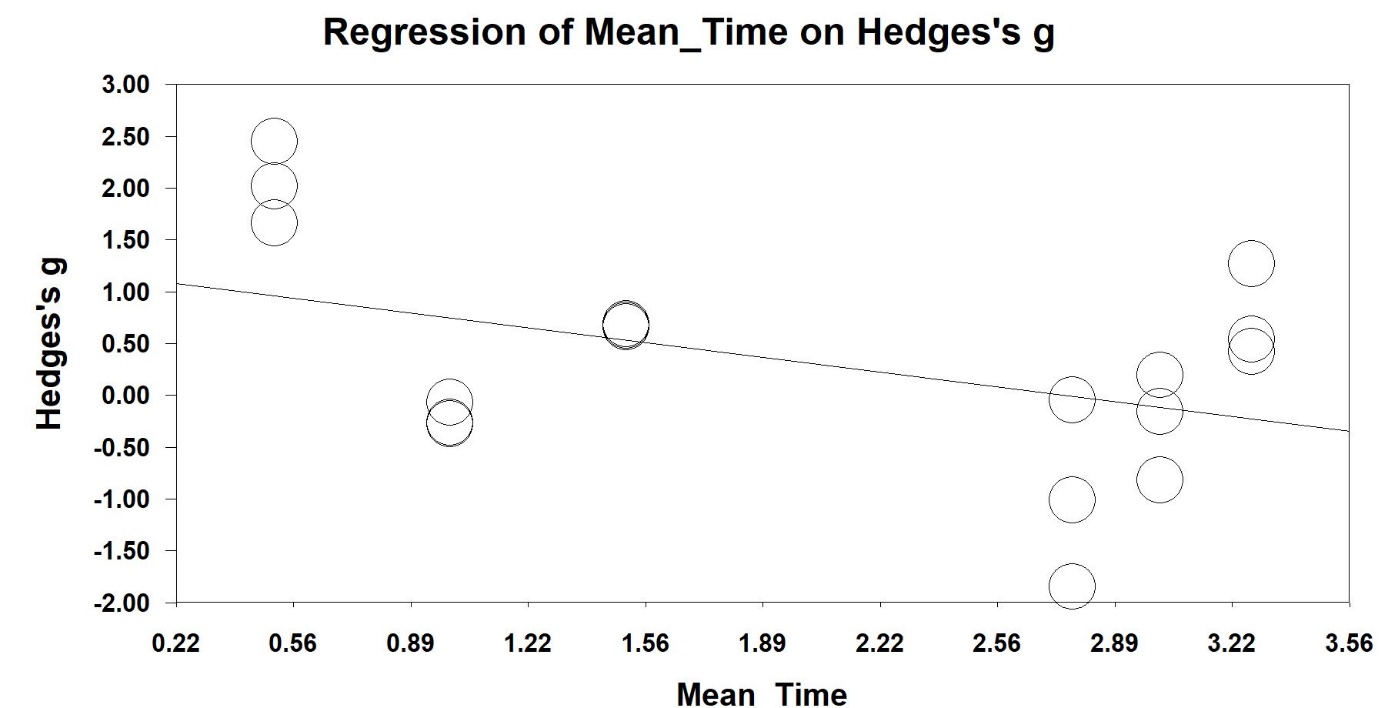
**

**Supplementary Figure n.17 - vMAT mRNA in hyperinsulinemia, main and subgroup analyses**

**
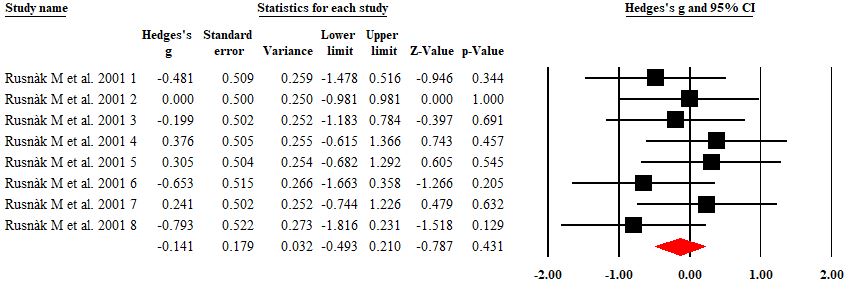
**

**
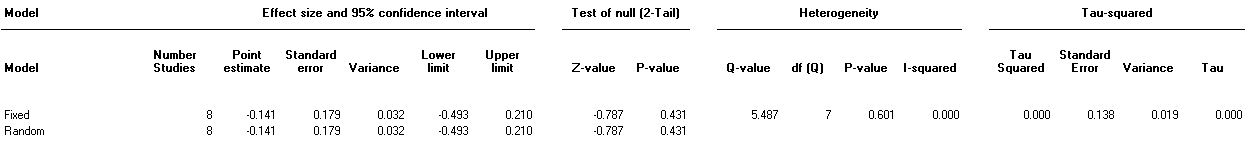
**

**Supplementary Figure n.18 - Cerebellar [3H]-d-aspartate binding in hyperinsulinemia, main and subgroup analysis**

**
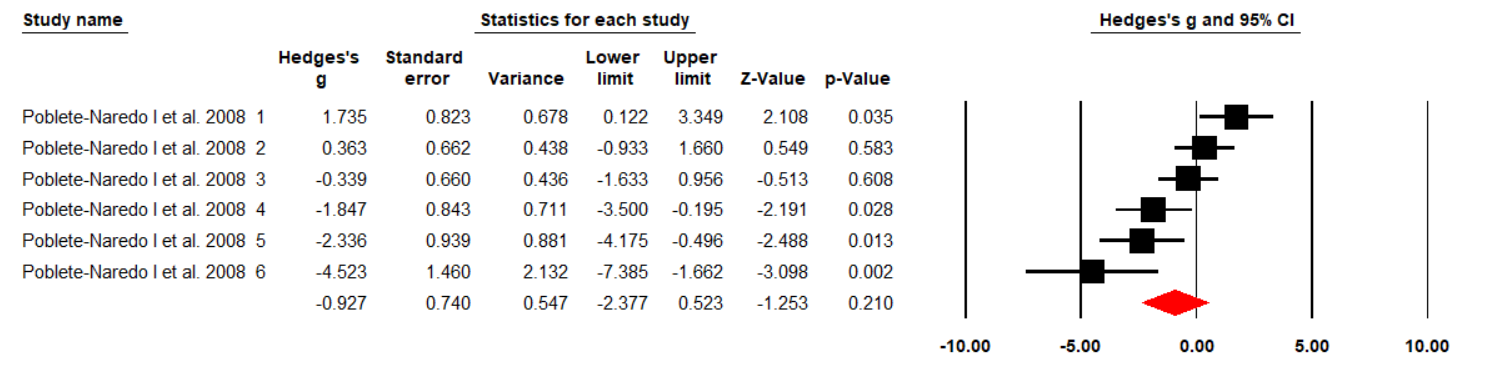
**


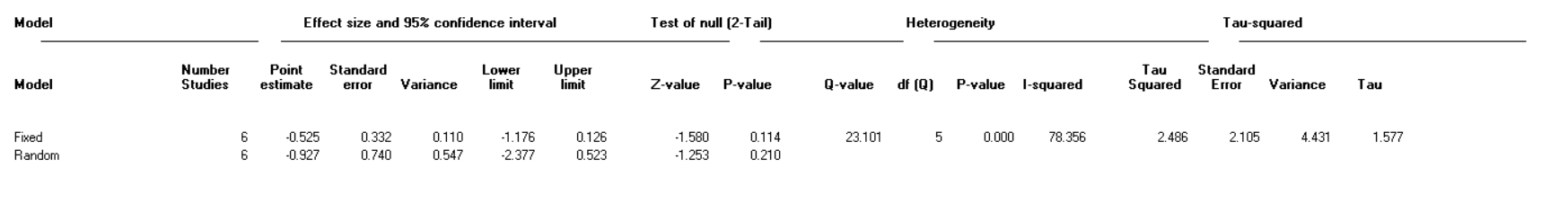


**Supplementary Figure n.19 - EPSCs in hyperinsulinemia, main and subgroup analysis**

**
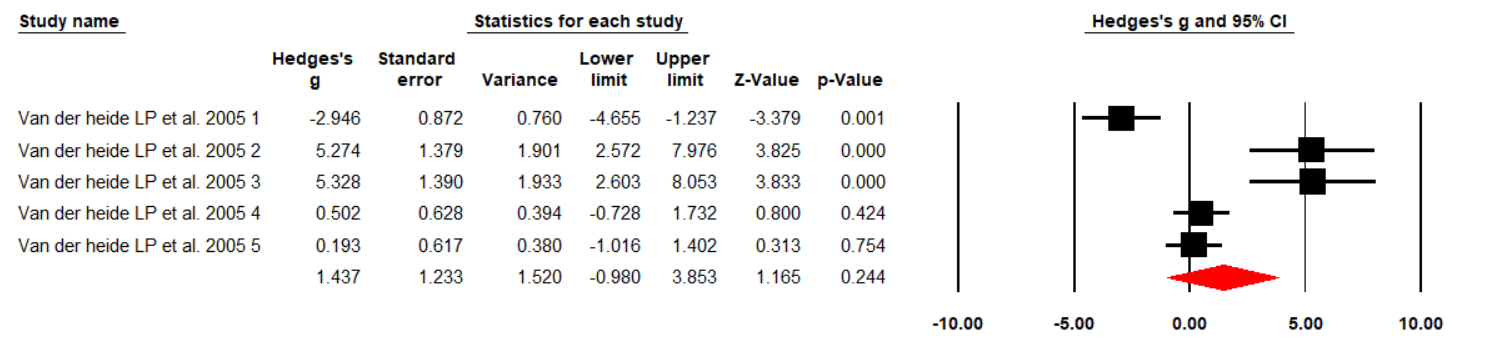
**

**
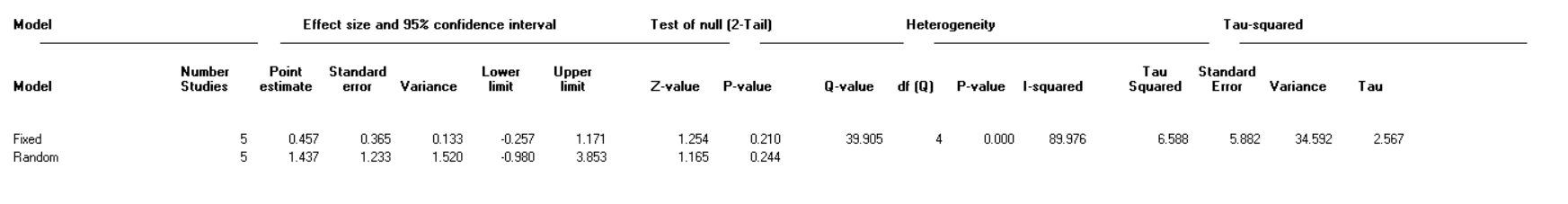
**

**Supplementary Figure n.20 – GLUR1 protein expression in hyperinsulinemia, main and subgroup analysis**


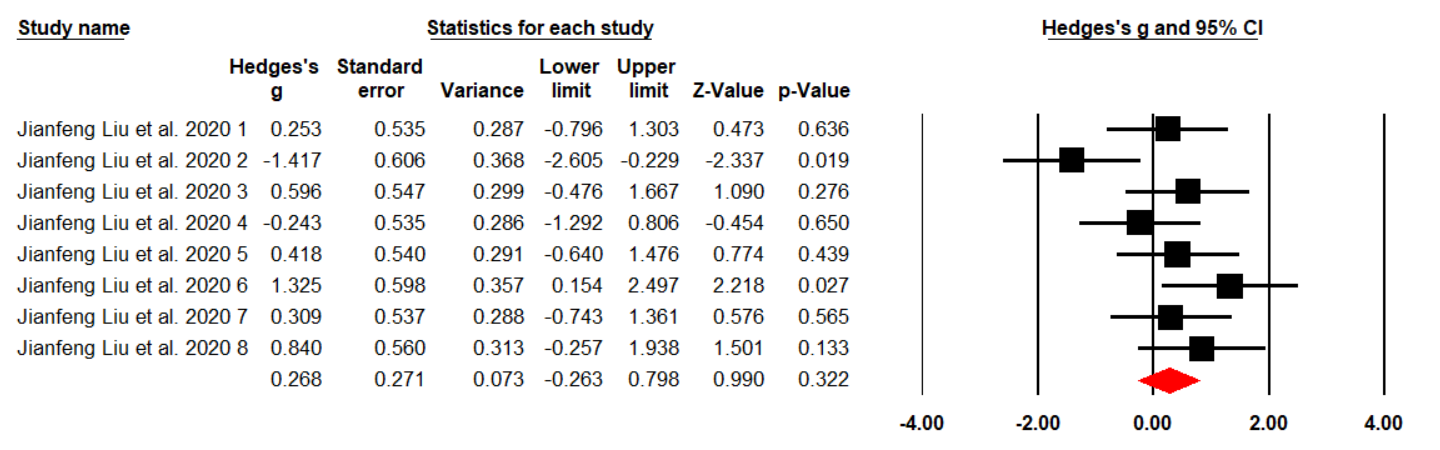


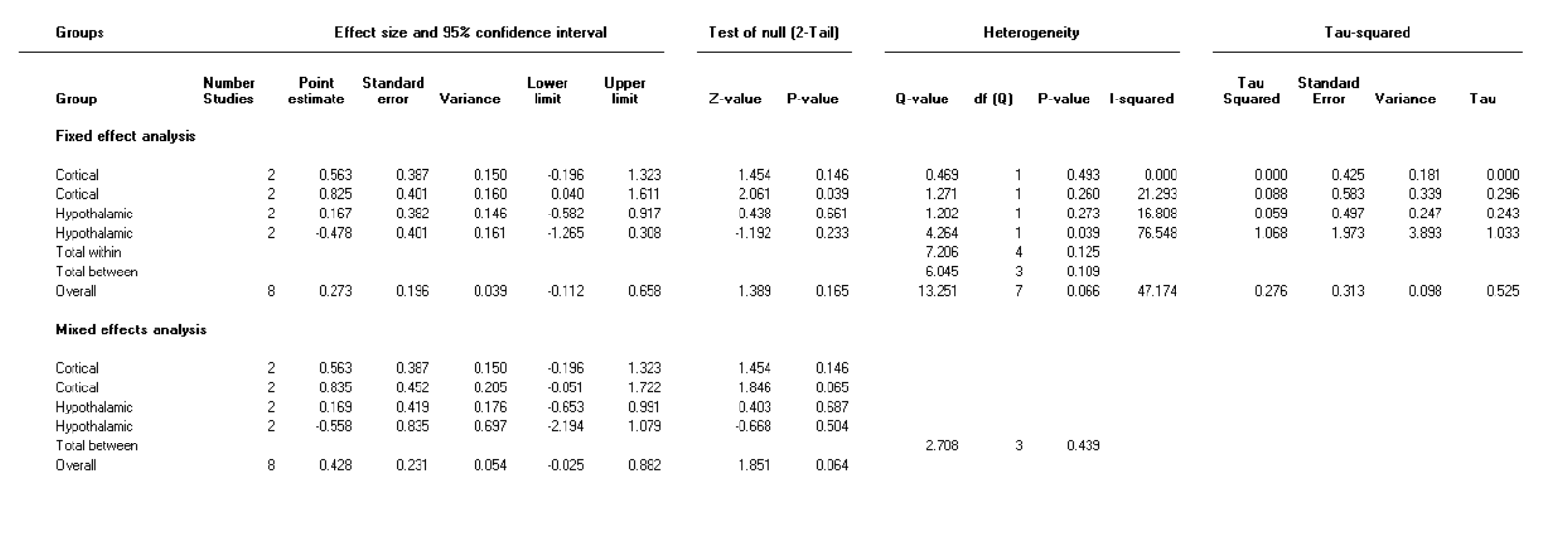


**Supplementary Figure n.21 – GLUR2 protein expression in hyperinsulinemia, main and subgroup analysis**


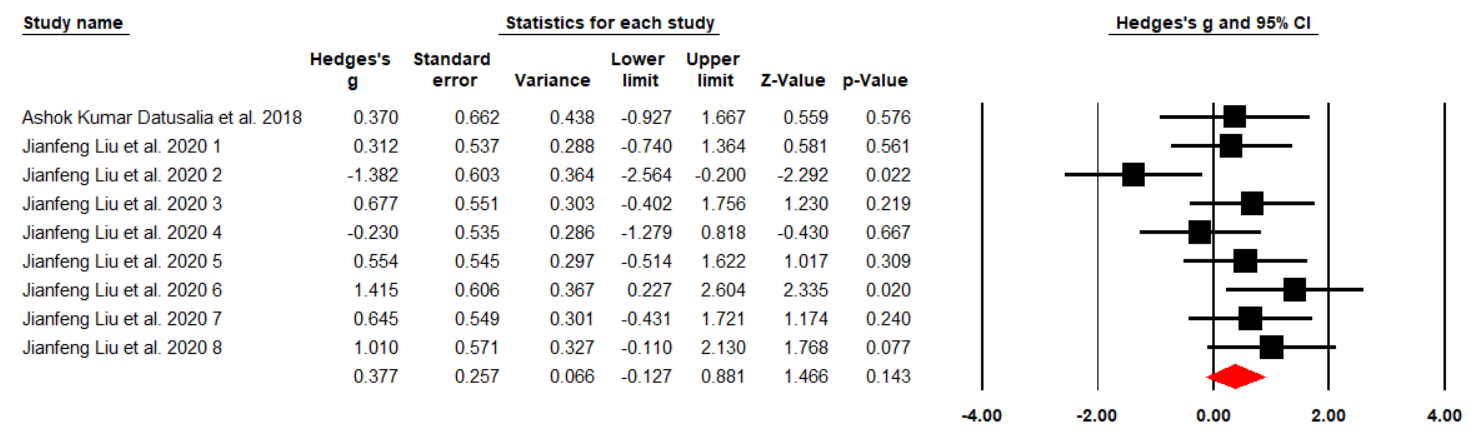


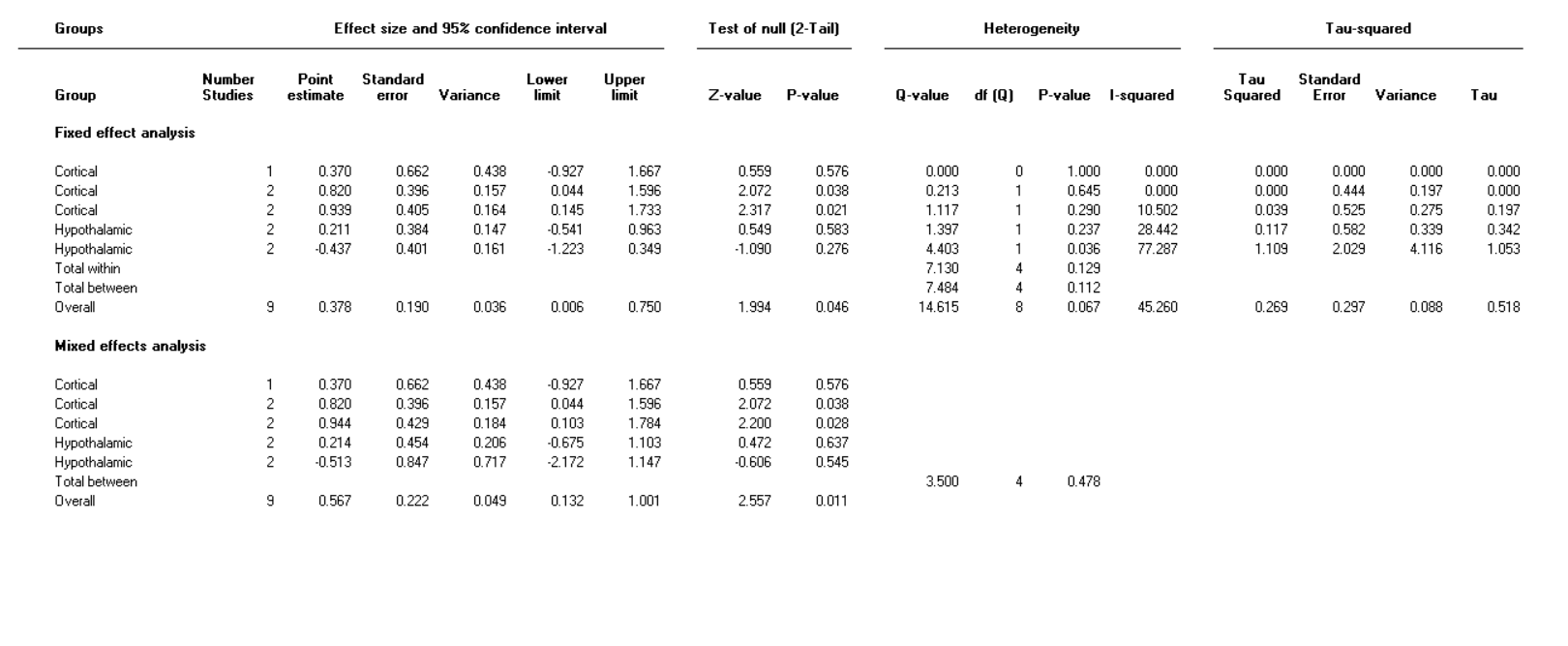


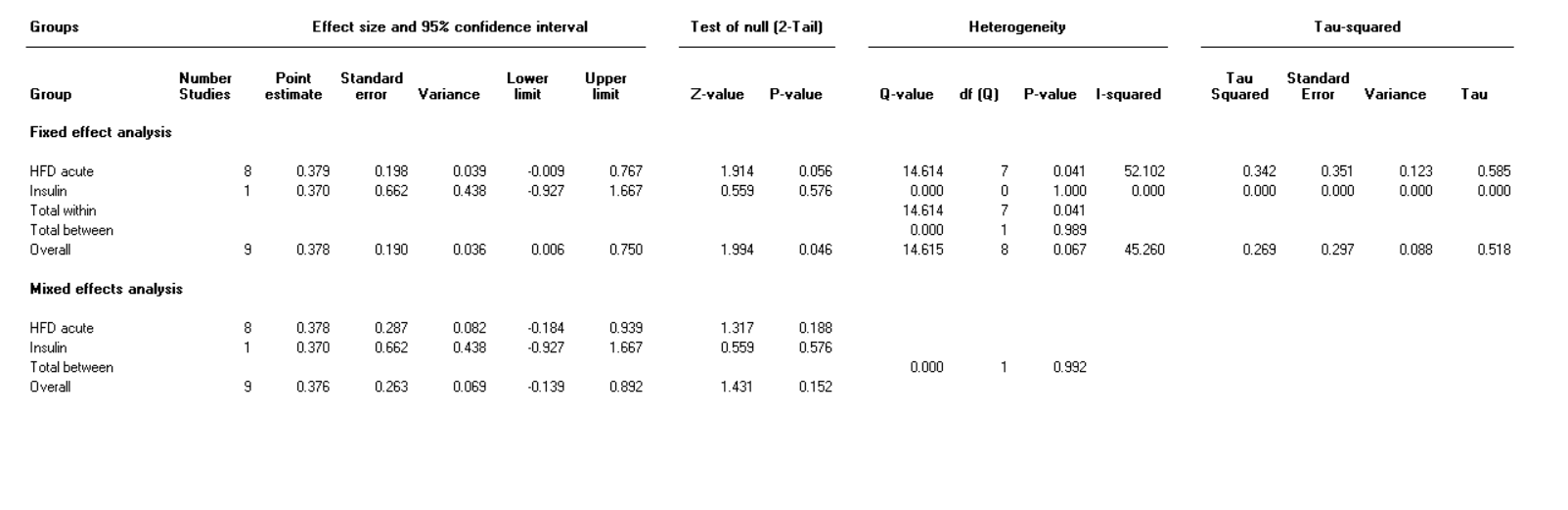


**Supplementary Figure n.22 –** **GLUR1 phosphorylation at Ser831 in hyperinsulinemia, main and subgroup analysis**


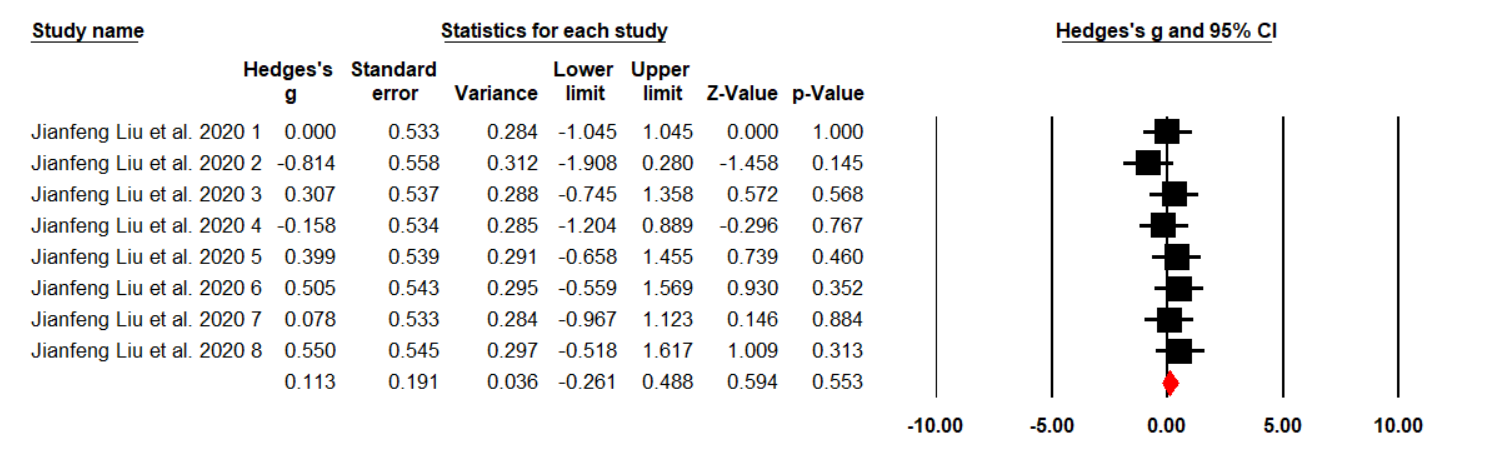


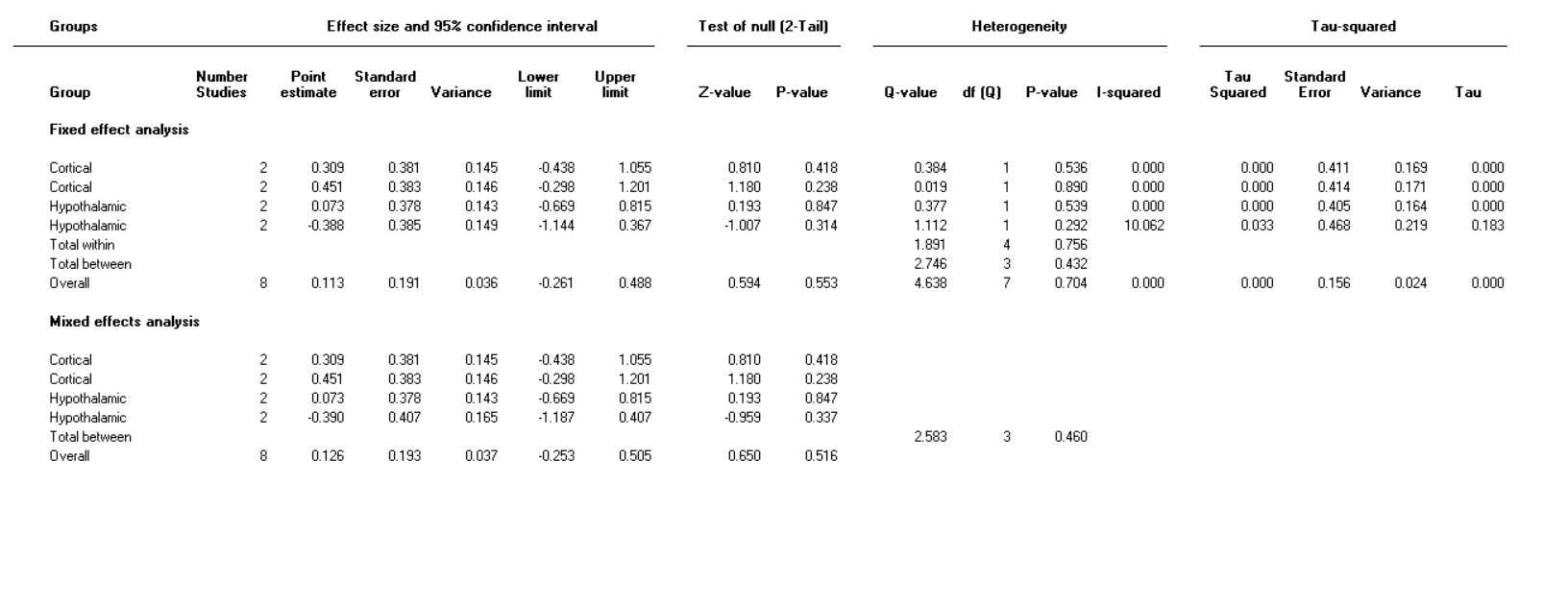


**Supplementary Figure n.23 –** **GLUR1 phosphorylation at Ser845 in hyperinsulinemia, main and subgroup analysis**


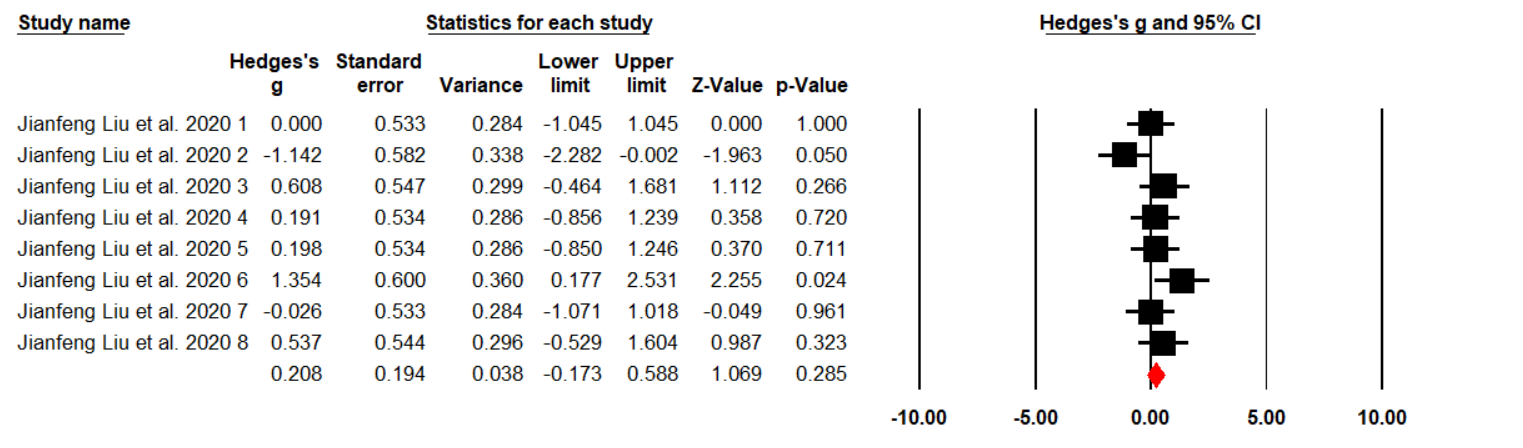


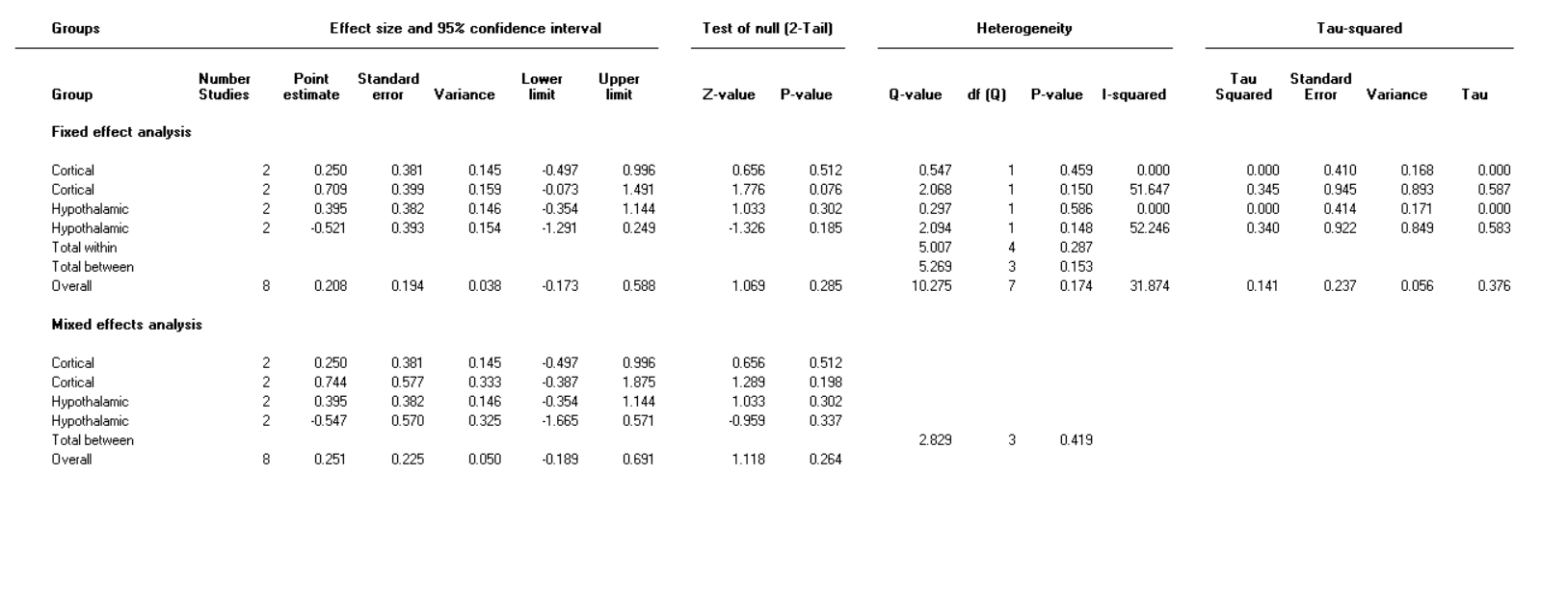


**Supplementary Figure n.24 –** **GLUR5 mRNA in hyperinsulinemia, main and subgroup analysis**


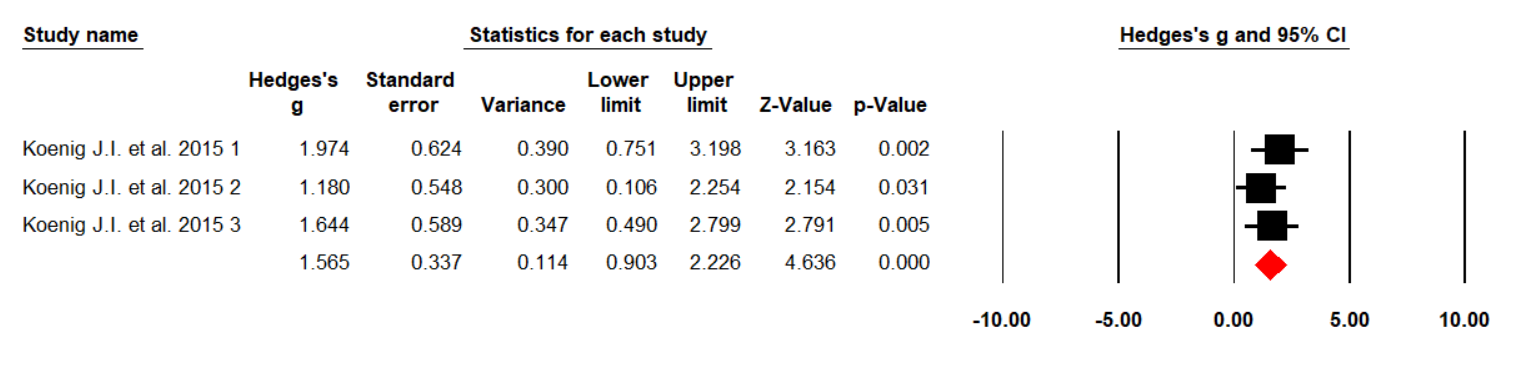


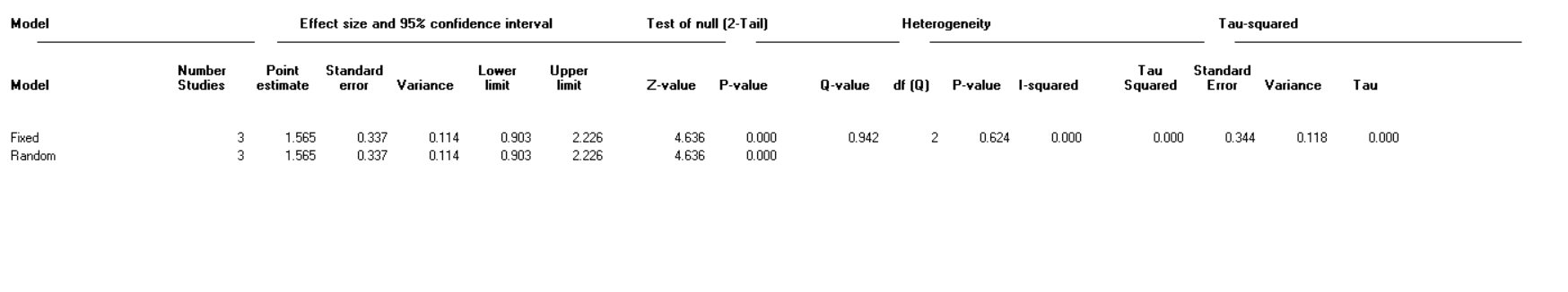


**Supplementary Figure n.25 –** **GLUR6 mRNA in hyperinsulinemia, main and subgroup analysis**

**Supplementary Figure n.26 –** **Glutamate Concentration in hyperinsulinemia, main and subgroup analysis**

**Analysis of glutamate concentration in Sprague-Dawley rats**

**Meta-regression of blood glucose ratio (cases/controls) in Sprague-Dawley rats**

**Supplementary Figure n.28 –** **Glutamine Concentration in hyperinsulinemia, main and subgroup analysis**

**Analysis of glutamine concentration in Sprague-Dawley rats**

**Meta-regression of time from insulin injection in Sprague-Dawley rats**

**Meta-regression of blood glucose ratio (controls/cases) in Sprague-Dawley rats**

**Supplementary Figure n.28 –** **Glutamine Synthase Activity in hyperinsulinemia, main and subgroup analysis**

**Supplementary Figure n.29 –** **Glycine concentration in hyperinsulinemia, main and subgroup analysis**

**Supplementary Figure n.30 –KA2 mRNA expression in hyperinsulinemia, main and subgroup analysis**

**Supplementary Figure n.31 –** **mGluR-5 mRNA expression in hyperinsulinemia, main and subgroup analysis**

**Supplementary Figure n.32 –** **Serine concentration in hyperinsulinemia, main and subgroup analysis**

**Supplementary Figure n. 33 –** **Glutamate dehydrogenase activity in hyperinsulinemia, main and subgroup analysis**

**Supplementary Figure n.34 – Cerebellar** **GLAST mRNA expression in hyperinsulinemia, main and subgroup analysis**

**Supplementary Figure n.35 –** **GAD activity rate in hyperinsulinemia, main and subgroup analysis**

**Supplementary Figure n.36 –** **GAD mRNA expression in hyperinsulinemia, main and subgroup analysis**

**Supplementary Figure n.37 –** **GAD65/67 protein expression in the** **Ventromedial Nucleus of the Hypothalamus in hyperinsulinemia, main and subgroup analysis**

**Meta-regression of blood glucose ratio (cases/controls)**

**Meta-regression of time from insulin injection (cases/controls)**

**Supplementary Figure n.38 - 5-HTP accumulation in hyperinsulinemia, main and subgroup analysis, publication bias, and meta-regression**

**Meta-regression of time from insulin injection**

**Supplementary Figure n.39 - 5HIAA concentration in hyperinsulinemia, main and subgroup analysis, publication bias, and meta-regression**

**Meta-regression of time from insulin injection in Sprague-Dawley rats**

**Meta-regression of time from insulin injection in Wistar rats**

**Meta-regression of blood glucose ratio (cases/controls) in Wistar rats**

**Supplementary Figure n.40 - 5-HT concentration in hyperinsulinemia, main and subgroup analysis, publication bias, and meta-regression**

**Meta-regression of time from insulin injection in Wistar rats**

**Meta-regression of blood glucose ratio in Wistar rats**

**Supplementary Figure n.41 - Tryptophan concentration in hyperinsulinemia, main and subgroup analysis, publication bias, and meta-regression**

**Meta-regression of time from insulin injection in Wistar rats**

**Supplementary Figure n.42 -** **Tryptophan accumulation in hyperinsulinemia, main analysis**

**Supplementary Figure n.43 - Bmax GABA Receptor in hyperinsulinemia, main and subgroup analysis**

**Supplementary Figure n.44 - Kd GABA Receptor in hyperinsulinemia, main and subgroup analysis**

**Supplementary Figure n.45 – Hippocampal GABA-A receptor β2/β3 subunits in hyperinsulinemia, main and subgroup analysis**

**Supplementary Figure n.46 – Gaba A alpha 1 mRNA expression in hyperinsulinemia, main and subgroup analysis**

**Supplementary Figure n.47 –** **GABA turnover in hyperinsulinemia, main and subgroup analysis**

**Supplementary Figure n.48 –** **GABA concentration in hyperinsulinemia, main and subgroup analysis**

**Analysis of GABA concentration in Sprague-Dawley rats**

**Meta-regression of time from insulin administration in Sprague-Dawley rats**

**Meta-regression of blood glucose ratio (cases/controls) administration in Sprague-Dawley rats**

**Supplementary Figure n.49 –** **mIPSC amplitude in hyperinsulinemia, main and subgroup analysis**

**Supplementary Figure n.50 –** **mIPSC frequency in hyperinsulinemia, main and subgroup analysis**

**Supplementary Figure n.51 –** **mIPSCs total current density in hyperinsulinemia, main and subgroup analysis**

**Supplementary Figure n.52 –** **sIPSCs amplitude in hyperinsulinemia, main and subgroup analysis**

**Supplementary Figure n.53 –** **sIPSCs frequency in hyperinsulinemia, main and subgroup analysis**

**Supplementary Figure n.54 –** **Tonic currents amplitude (GABA-A) in CA1 pyramidal neurons in hyperinsulinemia, main and subgroup analysis**

**Supplementary Figure n.55 –** **sIPSCs total current density in hyperinsulinemia, main and subgroup analysis**

**Supplementary Figure n.56 – [3H]DA-uptake, main and subgroup analysis**

**Supplementary Figure n.57 – DOPA concentration, main and subgroup analysis**

**Supplementary Figure n.58 – DOPAC concentration, main and subgroup analysis**

**Meta-regression of time from insulin manipulation**

**Meta-regression of blood insulin ratio (cases/controls)**

**Meta-regression of blood glucose ratio (cases/controls)**

**Supplementary Figure n.59 – DA concentration in hypoinsulinemic model, main and subgroup analysis**

**Meta-regression of time from insulin manipulation**

**Meta-regression of blood glucose ratio (cases/controls)**

**Supplementary Figure n.60 – DA turnover in hypoinsulinemic model, main and subgroup analysis**

**Supplementary Figure n.61 – D1R mRNA in hypoinsulinemic model, main and subgroup analysis**

**Supplementary Figure n.62 – D2R B_max_ in hypoinsulinemic model, main and subgroup analysis**

**Supplementary Figure n.63 – D2R K_d_ in hypoinsulinemic model, main and subgroup analysis**

**Supplementary Figure n.64 – D2R mRNA in hypoinsulinemic model, main and subgroup analysis**

**Supplementary Figure n.65 – DR B_max_ in hypoinsulinemic model, main and subgroup analysis**

**Supplementary Figure n.66 – DR K_d_ in hypoinsulinemic model, main and subgroup analysis**

**Supplementary Figure n.67 – HVA concentration in hypoinsulinemic model, main and subgroup analysis**

**Meta-regression of time from insulin alteration**

**Meta-regression of blood glucose ratio (cases/controls)**

**Supplementary Figure n.68 – TH+ neurons in hypoinsulinemic model, main and subgroup analysis**

**Supplementary Figure n.69 – TH activity in hypoinsulinemic model, main and subgroup analysis**

**Supplementary Figure n.70 – MAO activity in hypoinsulinemic model, main and subgroup analysis**

**Supplementary Figure n.71 –** **3[H]-NMDA-R binding in hypoinsulinemia, main and subgroup analysis**

**Supplementary Figure n.72 –** **3[H]-AMPA-R binding in hypoinsulinemia, main and subgroup analysis**

**Supplementary Figure n.73 –** **EAAT-1 protein expression in hypoinsulinemia, main and subgroup analysis**

**Supplementary Figure n.74 –** **EAAT-2 protein expression in hypoinsulinemia, main and subgroup analysis**

**Supplementary Figure n.75 –** **Glutamate Clearance in hypoinsulinemia, main and subgroup analysis**

**Supplementary Figure n.76 –** **Glutamate Concentration in hypoinsulinemia, main and subgroup analysis**

**Supplementary Figure n.77 –** **Glutamate uptake in hypoinsulinemia, main and subgroup analysis**

**Supplementary Figure n.78 –** **Glutamine levels in hypoinsulinemia, main and subgroup analysis**

**Supplementary Figure n.79 –** **mGluR-5 RNA levels in hypoinsulinemia, main and subgroup analysis**

**Supplementary Figure n.80 –** **NMDA-R Bmax in hypoinsulinemia, main and subgroup analysis**

**Supplementary Figure n.81 –** **NMDA-R Kd in hypoinsulinemia, main and subgroup analysis**

**Supplementary Figure n.82 –** **Hippocampal NR1 levels in hypoinsulinemia, main and subgroup analysis**

**Supplementary Figure n.83 –** **Hippocampal NR2B protein levels in hypoinsulinemia, main and subgroup analysis**

**Supplementary Figure n.84 –** **Glutamate Dehydrogenase Vmax in hypoinsulinemia, main and subgroup analysis**

**Supplementary Figure n.85 –** **Glutamate Dehydrogenase Km in hypoinsulinemia, main and subgroup analysis**

**Supplementary Figure n.86 –** **D-serine levels in hypoinsulinemia, main and subgroup analysis**

**Supplementary Figure n.87 –** **L-serine levels in hypoinsulinemia, main and subgroup analysis**

**Supplementary Figure n.88 –** **Glycine levels in hypoinsulinemia, main and subgroup analysis**

**Supplementary Figure n.89 – GAD mRNA in hypoinsulinemic models, main and subgroup analysis**

**Supplementary Figure n.90 - 5-HIAA concentration in hypoinsulinemia, main and subgroup analysis**

**Analysis in C57Bl6 mice**

**Analysis in albino Sprague-Dawley Rats**

**Analysis in Sprague-Dawley Rats**

**Meta-regression of time from insulin alteration**

**Analysis in albino Wistar Rats**

**Analysis in Wistar Rats**

**Meta-regression of blood glucose ratio (cases/controls)**

**Meta-regression of blood insulin ratio (cases/controls)**

**Meta-regression of time from insulin alteration**

**Analysis in Zucker Rats**

**Meta-regression of time from insulin alteration**

**Meta-regression of blood insulin ratio (cases/controls)**

**Supplementary Figure n.91 - 5-HT concentration in hypoinsulinemia, main and subgroup analysis**

**Analysis in albino Sprague-Dawley Rats**

**Analysis in albino Wistar Rats**

**Meta-regression of time from insulin alteration**

**Meta-regression of blood glucose ratio (cases/controls)**

**Analysis in C57Bl6 mice**

**Analysis in Sprague-Dawley Rats**

**Meta-regression of time from insulin alteration**

**Meta-regression of blood glucose ratio (cases/controls)**

**Analysis in Wistar Rats**

**Meta-regression of time from insulin alteration**

**Meta-regression of blood insulin ratio (cases/controls)**

**Meta-regression of blood glucose ratio (cases/controls)**

**Analysis in Zucker Rats**

**Meta-regression of time from insulin alteration**

**Meta-regression of blood insulin ratio (cases/controls)**

**Supplementary Figure n.92 - 5-HT2a Bmax in hypoinsulinemia, main and subgroup analysis**

**Supplementary Figure n.93 - 5-HT2a Kd in hypoinsulinemia, main and subgroup analysis**

**Supplementary Figure n.94 – Serotonin Receptor Bmax in hypoinsulinemia, main and subgroup analysis**

**Supplementary Figure n.95 – Serotonin Receptor Kd in hypoinsulinemia, main and subgroup analysis**

**Supplementary Figure n.95 – SERT mRNA in hypoinsulinemia, main and subgroup analysis**

**Supplementary Figure n.97 – Tryptophan concentration in hypoinsulinemia, main and subgroup analysis**

**Meta-regression of time from streptozotocin injection in rats**

**Supplementary Figure n.98 – 5-HTP concentration in hypoinsulinemia, main and subgroup analysis**

**Supplementary Figure n.99 – Tryptophan** **hydroxylase activity in hypoinsulinemia, main and subgroup analysis**

**Supplementary Figure n.100 – Tryptophan-5-hydroxylase Km in hypoinsulinemia, main and subgroup analysis**

**Supplementary Figure n.101 –** **Tryptophan-5-hydroxylase Vmax in hypoinsulinemia, main and subgroup analysis**

**Supplementary Figure n.102 – GABA-R B_max_ in hypoinsulinemic models, main and subgroup analysis**

**Supplementary Figure n.103 – GABA-R K_d_ in hypoinsulinemic models, main and subgroup analysis**

**Supplementary Figure n.104 – GABA concentration in hypoinsulinemic models, main and subgroup analysis**

**Supplementary Figure n.105 – GABA tonic current amplitude in hypoinsulinemic models, main and subgroup analysis**

**Supplementary Figure n.106 – GABA tonic current density in hypoinsulinemic models, main and subgroup analysis**

**Supplementary Figure n.107 – GABA-A alfa1 mRNA in hypoinsulinemic models, main and subgroup analysis**

**Supplementary Figure n.108 - DAT Bmax in an insulin-resistant model, main and subgroup analysis**

**Supplementary Figure n.109 - DAT Km in an insulin-resistant model, main and subgroup analysis**

**Supplementary Figure n.110 - DAT Vmax in an insulin-resistant model, main and subgroup analysis**

**Supplementary Figure n.111 - DAT mRNA in an insulin-resistant model, main and subgroup analysis**

**Supplementary Figure n.112 - DAT protein expression in an insulin-resistant model, main and subgroup analysis**

**Supplementary Figure n.113 - DAT cell surface expression in an insulin-resistant model, main and subgroup analysis**

**Supplementary Figure n.114 - DOPAC concentration in an insulin-resistant model, main and subgroup analysis**

**Supplementary Figure n.115 - D2R protein expression in an insulin-resistant model, main and subgroup analysis**

**Supplementary Figure n.116 – Striatal dopamine clearance in an insulin-resistant model, main and subgroup analysis**

**Supplementary Figure n.117 – Dopamine concentration in an insulin-resistant model, main and subgroup analysis**

**Meta-regression for the time from insulin pathway alteration**

**Supplementary Figure n.118 – Dopamine Extraction Fraction in an insulin-resistant model, main and subgroup analysis**

**Supplementary Figure n.119 – Dopamine Extracellular Concentration in the nucleus accumbens of an insulin-resistant model, main and subgroup analysis**

**Supplementary Figure n.120 – HVA levels in insulin-resistant model, main and subgroup analysis**

**Supplementary Figure n.121 –** **TH mRNA in an insulin-resistant model, main and subgroup analysis**

**Supplementary Figure n.122 –** **TH protein levels in insulin-resistant model, main and subgroup analysis**

**Supplementary Figure n.123 – Hippocampal SAP-102 protein levels in an insulin-resistant model, main and subgroup analysis**

**Supplementary Figure n.124 – Hippocampal NR2A protein levels in an insulin-resistant model, main and subgroup analysis**

**Supplementary Figure n.125 – Hippocampal NR2B protein levels in an insulin-resistant model, main and subgroup analysis**

**Sensitivity analysis excluding Li Jin et al 2018**

**Supplementary Figure n.126 – Glutamate clearance in an insulin-resistant model, main and subgroup analysis**

**Supplementary Figure n.127 – Glutamate concentration in an animal insulin-resistant model, main and subgroup analysis**

**Supplementary Figure n.128 – Glutamate concentration in a human insulin-resistant model, main and subgroup analysis**

**Supplementary Figure n.129 – Glutamine concentration in an animal insulin-resistant model, main and subgroup analysis**

**Supplementary Figure n.130 – Glutamine concentration in a human insulin-resistant model, main and subgroup analysis**

**Supplementary Figure n.131– PSD-95 protein levels in insulin-resistant model, main and subgroup analysis**

**Supplementary Figure n.132 – vGlut1 protein expression in an insulin-resistant model, main and subgroup analysis**

**Supplementary Figure n.133 – vGlut2 protein expression in an insulin-resistant model, main and subgroup analysis**

**Supplementary Figure n.134 – GAD-65 protein expression in an insulin-resistant model, main and subgroup analysis**

**Supplementary Figure n.135 – Hippocampal GAD-67 protein expression in an insulin-resistant model, main analysis**

**Supplementary Figure n.136 – Glutamate uptake in an insulin-resistant model, main analysis**

**Supplementary Figure n.137 - 5-HIAA concentration in an insulin-resistant model, main and subgroup analysis**

**Meta-regression of time from insulin alteration**

**Meta-regression of age (days) in Zucker rats**

**Supplementary Figure n.138 - 5-HT concentration in an insulin-resistant model, main and subgroup analysis**

**Supplementary Figure n.139 - Tryptophan concentration in an insulin-resistant model, main and subgroup analysis**

**Supplementary Figure n.140 - GABA concentration in an insulin-resistant model, main and subgroup analysis**

**Supplementary Figure n.141 - VGAT protein levels in an insulin-resistant model, main and subgroup analysis**

**Supplementary Figure n.142 – GABA uptake in an insulin-resistant model, main analysis**

**Supplementary Figure n.143 - DAT mRNA in brain insulin-resistant model, main and subgroup analysis**

**Supplementary Figure n.144 - Dopamine 1 receptor mRNA in brain insulin-resistant model, main and subgroup analysis**

**Supplementary Figure n.145 - Dopamine 2 receptor mRNA in brain insulin-resistant model, main and subgroup analysis**

**Supplementary Figure n.146 - Dopamine 3 receptor mRNA in brain insulin-resistant model, main and subgroup analysis**

**Supplementary Figure n.147 - Dopamine 5 receptor mRNA in brain insulin-resistant model, main and subgroup analysis**

**Supplementary Figure n.148 - MAO-B mRNA in brain insulin-resistant model, main and subgroup analysis**

**Supplementary Figure n.149 - TH mRNA in brain insulin-resistant model, main and subgroup analysis**

**Supplementary Figure n.150 - vMAT1 mRNA in brain insulin-resistant model, main and subgroup analysis**

**Supplementary Figure n.151 – vMAT2 mRNA in brain insulin-resistant model, main and subgroup analysis**

**Supplementary Figure n.152 – Dopamine levels in brain insulin-resistant model, main and subgroup analysis**

**Supplementary Figure n.153 – Hippocampal action potentials number in brain insulin-resistant model, main and subgroup analysis**

**Supplementary Figure n.154 – Hippocampal NR2A protein levels in brain insulin-resistant model, main and subgroup analysis**

**Supplementary Figure n.155 – Hippocampal NR2B protein levels in brain insulin-resistant model, main and subgroup analysis**

**Supplementary Figure n.156 – Glutamate concentration in brain insulin-resistant model, main and subgroup analysis**

**References**

1. Agardh CD--C, A.-//-Lindqvist, M.-//-Siesjo, B. K. The effect of pronounced hypoglycemia on monoamine metabolism in rat brain. *Diabetes* 1979; **28**(9)**:** 804-809.

2. Berggren U--E, J.-//-Liljequist, S. Differential effects of insulin on brain monoamine metabolism in rats. *Acta Pharmacologica et Toxicologica* 1983; **53**(1)**:** 39-43.

3. Chance WT--C, L.-//-Fischer, J. E. Brain 3-methoxytyramine varies inversely with blood glucose in decapitated rats. *Pharmacology Biochemistry and Behavior* 1989; **32**(2)**:** 553-556.

4. Figlewicz DP--S, P.-//-Chavez, M.-//-Woods, S. C.-//-Veith, R. C. Intraventricular insulin increases dopamine transporter mRNA in rat VTA/substantia nigra. *Brain Research* 1994; **644**(2)**:** 331-334.

5. Grunstein HS--S, G. A.-//-Bradshaw, J. E.-//-Compton, P. J. Tolbutamide increases hypothalamic serotonin activity in the rat. *Diabetes* 1986; **35**(4)**:** 475-480.

6. Gupta G--A, M.-//-Baquer, N. Z. Effect of experimental diabetes on the catecholamine metabolism in rat brain. *Journal of Neurochemistry* 1992; **58**(1)**:** 95-100.

7. Guzmán DC--B, N. O.-//-Herrera, M. O.-//-Peraza, A. V.-//-Juárez-Olguín, H.-//-Mejía, G. B. Insulin plus zinc induces a favorable biochemical response effects on oxidative damage and dopamine levels in rat brain. *International Journal of Biological Macromolecules* 2019; **132:** 230-235.

8. Guzmán DC--B, N. O.-//-Herrera, M. O.-//-Peraza, A. V.-//-Mejía, G. B.-//-Olguín, H. J.-//-Jiménez, F. T. Sildenafil alters biogenic amines and increases oxidative damage in brain regions of insulin-hypoglycemic rats. *Acta Pharmaceutica* 2020; **70**(1)**:** 121-127.

9. Lozovsky DB--K, I. J.-//-Saller, C. F. Modulation of dopamine receptor supersensitivity by chronic insulin: Implication in schizophrenia. *Brain Research* 1985; **343**(1)**:** 190-193.

10. Orosco M--R, C.-//-Gripois, D.-//-Blouquit, M. F.-//-Roffi, J.-//-Jacquot, C.-//-Cohen, Y. Effects of insulin on brain monoamine metabolism in the Zucker rat: Influence of genotype and age. *Psychoneuroendocrinology* 1991; **16**(6)**:** 537-546.

11. Robinson R--K, A.-//-Paulose, C. S. Enhanced dopamine D1 and D2 receptor gene expression in the hippocampus of hypoglycaemic and diabetic rats. *Cellular and Molecular Neurobiology* 2009; **29**(3)**:** 365-372.

12. Sauter A--G, M.-//-Engel, J.-//-Ueta, K. Effect of insulin on central catecholamines. *Brain Research* 1983; **260**(2)**:** 330-333.

13. Soengas JL--A, M. Brain glucose and insulin: effects on food intake and brain biogenic amines of rainbow trout. *Journal of comparative physiology A, Neuroethology, sensory, neural, and behavioral physiology* 2004; **190**(8)**:** 641-649.

14. Briski KP--A, M. H.-//-Napit, P. R. Sex-specific acclimation of A2 noradrenergic neuron dopamine-β-hydroxylase and estrogen receptor variant protein and 5'-AMP-Activated protein kinase reactivity to recurring hypoglycemia in rat. *J Chem Neuroanat* 2020; **109:** 101845.

15. Briski KP--A, M. H.-//-Napit, P. R.-//-Mahmood, Asmh-//-Alhamyani, A. R.-//-Alshamrani, A. A.-//-Ibrahim, M. M. H. Sex differences in ventromedial hypothalamic nucleus glucoregulatory transmitter biomarker protein during recurring insulin-induced hypoglycemia. *Brain Struct Funct* 2021; **226**(4)**:** 1053-1065.

16. Del Rio D--S, P.-//-Hernández-Nuño, F.-//-Cano, V.-//-Morales, L.-//-Chowen, J. A.-//-Del Olmo, N.-//-Ruiz-Gayo, M. Free-choice high-fat diet alters circadian oscillation of energy intake in adolescent mice: role of prefrontal cortex. *Eur J Nutr* 2017; **56**(5)**:** 1833-1844.

17. Figlewicz DP--VD, G.-//-Wilkinson, C. W.-//-Gronbeck, P.-//-Higgins, M.-//-Zavosh, A. Effects of repetitive hypoglycemia on neuroendocrine response and brain tyrosine hydroxylase activity in the rat. *Stress* 2002; **5**(3)**:** 217-226.

18. Fine JM--S, B. M.-//-Faltesek, K. A.-//-Terai, K.-//-Haase, L.-//-Knutzen, K. E.-//-Kosyakovsky, J.-//-Bowe, T. J.-//-Fuller, A. K.-//-Frey, W. H.-//-Hanson, L. R. Intranasal delivery of low-dose insulin ameliorates motor dysfunction and dopaminergic cell death in a 6-OHDA rat model of Parkinson's Disease. *Neurosci Lett* 2020; **714:** 134567.

19. Guzman DC--G, E. H.-//-Mejia, G. B.-//-Olguin, H. J.-//-Gonzalez, J. A.-//-Labra Ruiz, N. A. Effect of morphine and lacosamide on levels of dopamine and 5-HIAA in brain regions of rats with induced hypoglycemia. *Pak J Biol Sci* 2014; **17**(2)**:** 292-296.

20. Paulose CS--B, S. Adrenergic, dopaminergic and serotonergic gene expression in low dose, long time insulin and somatotropin treatment to ageing rats: rejuvenation of brain function. *Biogerontology* 2008; **9**(6)**:** 429-439.

21. Rusnák M--J, J.-//-Vietor, I.-//-Sabban, E. L.-//-Kvetnanský, R. Different effects of insulin and 2-deoxy-D-glucose administration on tyrosine hydroxylase gene expression in the locus coeruleus and the adrenal medulla in rats. *Brain Res Bull* 1998; **46**(5)**:** 447-452.

22. Mackenzie RG--T, M. E. EFFECTS OF INSULIN AND STREPTOZOTOCIN–NDUCED DIABETES ON BRAIN TRYPTOPHAN AND SEROTONIN METABOLISM IN RATS. *Journal of Neurochemistry* 1978; **30**(1)**:** 205-211.

23. Rusnák M--K, R.-//-Jeloková, J.-//-Palkovits, M. Effect of novel stressors on gene expression of tyrosine hydroxylase and monoamine transporters in brainstem noradrenergic neurons of long-term repeatedly immobilized rats. *Brain Research* 2001; **899**(1-2)**:** 20-35.

24. Shiraishi JI--Y, K.-//-Terao, H.-//-Matsuura, M.-//-Bungo, T. Involvement of the central monoaminergic system in insulin-induced anorexia in chicks. *Journal of Animal and Veterinary Advances* 2010; **9**(16)**:** 2109-2112.

25. Barry RL--B, N. E.-//-Williams, J. M.-//-Siuta, M. A.-//-Tantawy, M. N.-//-Speed, N. K.-//-Saunders, C.-//-Galli, A.-//-Niswender, K. D.-//-Avison, M. J. Brief exposure to obesogenic diet disrupts brain dopamine networks. *PLoS ONE* 2018; **13**(4).

26. Hajnal A--L, L. Feeding-related dopamine in the amygdala of freely moving rats. *NeuroReport* 1997; **8**(12)**:** 2817-2820.

27. Shimizu H--B, G. A. Effects of insulin on hypothalamic monoamine metabolism. *Brain Research* 1990; **510**(2)**:** 251-258.

28. Trulson ME--C, T.-//-Trulson, V. M. Dopamine-containing substantia nigra units are unresponsive to changes in plasma glucose levels induced by dietary factors glucose infusions or insulin administration in freely moving cats. *Life Sciences* 1983; **32**(22)**:** 2555-2564.

29. Stouffer MA--W, C. A.-//-Patel, J. C.-//-Lee, C. R.-//-Witkovsky, P.-//-Bao, L.-//-Machold, R. P.-//-Jones, K. T.-//-De Vaca, S. C.-//-Reith, M. E. A.-//-Carr, K. D.-//-Rice, M. E. Insulin enhances striatal dopamine release by activating cholinergic interneurons and thereby signals reward. *Nature Communications* 2015; **6**.

30. Knusel B--H, F. Trophic actions of IGF-I, IGF-II and insulin on cholinergic and dopaminergic brain neurons. *Adv Exp Med Biol* 1991; **293:** 351-360.

31. Mebel DM--W, J. C.-//-Dong, Y. J.-//-Borgland, S. L. Insulin in the ventral tegmental area reduces hedonic feeding and suppresses dopamine concentration via increased reuptake. *Eur J Neurosci* 2012; **36**(3)**:** 2336-2346.

32. Kullmann S, Blum D, Jaghutriz BA, Gassenmaier C, Bender B, Häring HU *et al.* Central Insulin Modulates Dopamine Signaling in the Human Striatum. *The Journal of clinical endocrinology and metabolism* 2021; **106**(10)**:** 2949-2961.

33. Huang CC--Y, J. L.-//-Lee, C. C.-//-Hsu, K. S. Insulin induces a novel form of postsynaptic mossy fiber long-term depression in the hippocampus. *Molecular and Cellular Neuroscience* 2003; **24**(3)**:** 831-841.

34. Datusalia AK--A, P.-//-Singh, J. N.-//-Sharma, S. S. Hyper-insulinemia increases the glutamate-excitotoxicity in cortical neurons: A mechanistic study. *Eur J Pharmacol* 2018; **833:** 524-530.

35. Duarte AI--S, M. S.-//-Seiça, R.-//-de Oliveira, C. R. Insulin affects synaptosomal GABA and glutamate transport under oxidative stress conditions. *Brain Res* 2003; **977**(1)**:** 23-30.

36. Kim SJ--H, Y. Insulin inhibits AMPA-induced neuronal damage via stimulation of protein kinase B (Akt). *J Neural Transm (Vienna)* 2005; **112**(2)**:** 179-191.

37. Poblete-Naredo I--A, C.-//-Hernández-Kelly, L.-//-López-Bayghen, E.-//-Aguilera, J.-//-Ortega, A. Insulin-dependent regulation of GLAST/EAAT1 in Bergmann glial cells. *Neurosci Lett* 2009; **451**(2)**:** 134-138.

38. van der Heide LP--K, A.-//-Artola, A.-//-Gispen, W. H.-//-Ramakers, G. M. Insulin modulates hippocampal activity-dependent synaptic plasticity in a N-methyl-d-aspartate receptor and phosphatidyl-inositol-3-kinase-dependent manner. *J Neurochem* 2005; **94**(4)**:** 1158-1166.

39. Plitzko D--R, S.-//-Gottmann, K. Insulin promotes functional induction of silent synapses in differentiating rat neocortical neurons. *European Journal of Neuroscience* 2001; **14**(8)**:** 1412-1415.

40. Sena A--FS, V.-//-Sarliéve, L. L.-//-Tholey, G. Differential modulation of glutamate metabolizing enzymes in mouse and chick cultured glial cells by insulin. *Journal of Neuroscience Research* 1988; **20**(2)**:** 189-194.

41. Spicarova D--P, J. Modulation of AMPA excitatory postsynaptic currents in the spinal cord dorsal horn neurons by insulin. *Neuroscience* 2010; **166**(1)**:** 305-311.

42. Tholey G--S, A. H.-//-Ledig, M. Specific Insulin‐Mediated Regulation of Glutamine Synthetase in Cultured Chick Astroglial Cells. *Journal of Neurochemistry* 1986; **47**(5)**:** 1490-1492.

43. Brass BJ--N, D.-//-Barrett, J. N. Differential Effects of Insulin on Choline Acetyltransferase and Glutamic Acid Decarboxylase Activities in Neuron‐Rich Striatal Cultures. *Journal of Neurochemistry* 1992; **59**(2)**:** 415-424.

44. Ichord RN--J, M. V.-//-Traystman, R. J. MK801 decreases glutamate release and oxidative metabolism during hypoglycemic coma in piglets. *Developmental Brain Research* 2001; **128**(2)**:** 139-148.

45. Silverstein FS--S, J.-//-Gordon, K. E. Hypoglycemia alters striatal amino acid efflux in perinatal rats: An in vivo microdialysis study. *Annals of Neurology* 1990; **28**(4)**:** 516-521.

46. Darling BK--A-R, M.-//-Moores, R. R.-//-Chang, A. S.-//-Howard, R. S.-//-O'Neill, J. T. Brain excitatory amino acid concentrations are lower in the neonatal pig: a buffer against excitotoxicity? *Biol Neonate* 2001; **80**(4)**:** 305-312.

47. Guyot LL--D, F. G.-//-O'Regan, M. H.-//-Song, D.-//-Phillis, J. W. Topical insulin and accumulation of excitotoxic and other amino acids in ischemic rat cerebral cortex (44510). *Experimental Biology and Medicine* 2000; **224**(1)**:** 28-31.

48. Abdul-Ghani AS--G, H.-//-el-Lati, S.-//-Saca'an, A. Changes in the activity of glutamate related enzymes in cerebral cortex, during insulin-induced seizures. *The International journal of neuroscience* 1989; **44**(1-2)**:** 67-74.

49. Koenig JI--C, J. Y. Provocation of kainic acid receptor mRNA changes in the rat paraventricular nucleus by insulin-induced hypoglycaemia. *Journal of Neuroendocrinology* 2005; **17**(2)**:** 111-118.

50. Labouèbe G--L, S.-//-Dias, C.-//-Zou, H.-//-Wong, J. C. Y.-//-Karunakaran, S.-//-Clee, S. M.-//-Phillips, A. G.-//-Boutrel, B.-//-Borgland, S. L. Insulin induces long-term depression of ventral tegmental area dopamine neurons via endocannabinoids. *Nature Neuroscience* 2013; **16**(3)**:** 300-308.

51. McGowan JE--H-L, A. G.-//-Mishra, O. P.-//-Delivoria-Papadopoulos, M. The effect of acute hypoglycemia on the cerebral NMDA receptor in newborn piglets. *Brain Research* 1995; **670**(2)**:** 283-288.

52. Arieff AI--D, T.-//-Zelig, H.-//-Massry, S. G. Mechanisms of seizures and coma in hypoglycemia. Evidence for a direct effect of insulin on electrolyte transport in brain. *J Clin Invest* 1974; **54**(3)**:** 654-663.

53. Balakrishnan S--M, J.-//-Paulose, C. S. Cholinergic and glutamergic receptor functional regulation in long-term, low dose somatotropin and insulin treatment to ageing rats: rejuvenation of brain function. *Mol Cell Endocrinol* 2010; **314**(1)**:** 23-30.

54. Butterworth RF--L, F.-//-Hamel, E.-//-Merkel, A.-//-Giguere, F.-//-Barbeau, A. Effect of asparagine, glutamine and insulin on cerebral amino acid neurotransmitters. *Can J Neurol Sci* 1980; **7**(4)**:** 447-450.

55. Butterworth RF--M, A. D.-//-Landreville, F. Regional amino acid distribution in relation to function in insulin hypoglycaemia. *J Neurochem* 1982; **38**(5)**:** 1483-1489.

56. Gorell JM--D, P. H.-//-Ferrendelli, J. A. Regional levels of glucose, amino acids, high energy phosphates, and cyclic nucleotides in the central nervous system during hypoglycemic stupor and behavioral recovery. *J Neurochem* 1976; **27**(5)**:** 1043-1049.

57. Gundersen V--F, F.-//-Ottersen, O. P.-//-Storm-Mathisen, J. Redistribution of neuroactive amino acids in hippocampus and striatum during hypoglycemia: a quantitative immunogold study. *J Cereb Blood Flow Metab* 2001; **21**(1)**:** 41-51.

58. Joseph A--A, S.-//-Paulose, C. S. Increased glutamate receptor gene expression in the cerebral cortex of insulin induced hypoglycemic and streptozotocin-induced diabetic rats. *Neuroscience* 2008; **156**(2)**:** 298-304.

59. Liu J--D, S.-//-Sawangjit, A.-//-Born, J.-//-Ehrlich, I.-//-Hallschmid, M. Short-term high-fat feeding induces a reversible net decrease in synaptic AMPA receptors in the hypothalamus. *J Nutr Biochem* 2021; **87:** 108516.

60. McGowan JE--Z, S. A.-//-Haynes-Laing, A. G.-//-Mishra, O. P.-//-Delivoria-Papadopoulos, M. Modification of glutamate binding sites in newborn brain during hypoglycemia. *Brain Res* 2002; **927**(1)**:** 80-86.

61. Muller AP--G, J.-//-Moreira, J. D.-//-Zimmer, E. R.-//-Haas, C. B.-//-Lulhier, F.-//-Perry, M. L.-//-Souza, D. O.-//-Torres-Aleman, I.-//-Portela, L. V. Exercise increases insulin signaling in the hippocampus: physiological effects and pharmacological impact of intracerebroventricular insulin administration in mice. *Hippocampus* 2011; **21**(10)**:** 1082-1092.

62. Petroff OA--Y, R. S.-//-Cowan, B. E.-//-Novotny, E. J., Jr. 1H nuclear magnetic resonance spectroscopy study of neonatal hypoglycemia. *Pediatr Neurol* 1988; **4**(1)**:** 31-34.

63. Telushkin PK--N, A. D.-//-Potapov, P. P.-//-Medvedeva, N. B.-//-Stel'makh, A. Y. Glycolysis and oxidtion enzyme activity in rat brain during insulin-induced hypoglycemia against the background of alloxan-induced diabetes mellitus. *Bull Exp Biol Med* 2005; **140**(6)**:** 695-697.

64. Agardh CD--F, J.-//-Siesjou, B. K. CEREBRAL METABOLIC CHANGES IN PROFOUND, INSULIN‐INDUCED HYPOGLYCEMIA, AND IN THE RECOVERY PERIOD FOLLOWING GLUCOSE ADMINISTRATION. *Journal of Neurochemistry* 1978; **31**(5)**:** 1135-1142.

65. Engelsen B--W, E.-//-Fonnum, F.-//-Wieloch, T. Effect of Insulin‐Induced Hypoglycemia on the Concentrations of Glutamate and Related Amino Acids and Energy Metabolites in the Intact and Decorticated Rat Neostriatum. *Journal of Neurochemistry* 1986; **47**(5)**:** 1634-1641.

66. Hernandez T--C, R. A. Effect of insulin on free amino acids in Caiman tissue and plasma. *Comparative Biochemistry And Physiology* 1968; **26**(3)**:** 991-996.

67. Beverly JL--M, R. J. Influence of serum glucose on glutamate decarboxylase activity in the ventromedial nucleus of rats. *American Journal of Physiology - Regulatory Integrative and Comparative Physiology* 1990; **258**(3 27-3)**:** R697-R703.

68. Sherin A--A, J.-//-Peeyush, K. T.-//-Smijin, S.-//-Anitha, M.-//-Roshni, B. T.-//-Paulose, C. S. Cholinergic and GABAergic receptor functional deficit in the hippocampus of insulin-induced hypoglycemic and streptozotocin-induced diabetic rats. *Neuroscience* 2012; **202:** 69-76.

69. Sherin A--P, K. T.-//-Naijil, G.-//-Chinthu, R.-//-Paulose, C. S. Hypoglycemia induced behavioural deficit and decreased GABA receptor, CREB expression in the cerebellum of streptozoticin induced diabetic rats. *Brain Res Bull* 2010; **83**(6)**:** 360-366.

70. Antony S--K, T. P.-//-Kuruvilla, K. P.-//-George, N.-//-Paulose, C. S. Decreased GABA receptor binding in the cerebral cortex of insulin induced hypoglycemic and streptozotocin induced diabetic rats. *Neurochem Res* 2010; **35**(10)**:** 1516-1521.

71. Alhamami HN--A, A.-//-Briski, K. P. Inhibition of glycogen phosphorylase stimulates ventromedial hypothalamic nucleus AMP-activated protein kinase: Activity and neuronal nitric oxide synthase protein expression in male rats. *Physiological Reports* 2017; **5**(23).

72. Alshamrani AA--B, K.-//-Alhamyani, A.-//-Ali, M. H.-//-Napit, P. R.-//-Uddin, M. M.-//-Mahmood, A. S. M. H.-//-Ibrahim, M. M. H.-//-Briski, K. P. Sex-dimorphic Rostro-caudal Patterns of 5′-AMP-activated Protein Kinase Activation and Glucoregulatory Transmitter Marker Protein Expression in the Ventrolateral Ventromedial Hypothalamic Nucleus (VMNvl) in Hypoglycemic Male and Female Rats: Impact of Estradiol. *Journal of Molecular Neuroscience* 2020.

73. Ibrahim MMH--B, K.-//-Alhamami, H. N.-//-Briski, K. P. Effects of Intracerebroventricular Glycogen Phosphorylase Inhibitor CP-316,819 Infusion on Hypothalamic Glycogen Content and Metabolic Neuron AMPK Activity and Neurotransmitter Expression in Male Rat. *Journal of Molecular Neuroscience* 2020; **70**(5)**:** 647-658.

74. Behar KL--dH, J. A.-//-Petroff, O. A.-//-Hetherington, H. P.-//-Prichard, J. W.-//-Shulman, R. G. Effect of hypoglycemic encephalopathy upon amino acids, high-energy phosphates, and pHi in the rat brain in vivo: detection by sequential 1H and 31P NMR spectroscopy. *J Neurochem* 1985; **44**(4)**:** 1045-1055.

75. Chowdhury GMI--W, P.-//-Ciardi, A.-//-Mamillapalli, R.-//-Johnson, J.-//-Zhu, W.-//-Eid, T.-//-Behar, K.-//-Chan, O. Impaired Glutamatergic Neurotransmission in the Ventromedial Hypothalamus May Contribute to Defective Counterregulation in Recurrently Hypoglycemic Rats. *Diabetes* 2017; **66**(7)**:** 1979-1989.

76. Aonuma H--T, Y.-//-Kaneda, M.-//-Nakamura, R.-//-Watanabe, T.-//-Hatakeyama, D.-//-Dyakonova, V. E.-//-Lukowiak, K.-//-Ito, E. Effects of 5-HT and insulin on learning and memory formation in food-deprived snails. *Neurobiology of Learning and Memory* 2018; **148:** 20-29.

77. Heyes MP--P, M.-//-Leonard, C.-//-Markey, S. P.-//-Auer, R. N. Brain and plasma quinolinic acid in profound insulin-induced hypoglycemia. *Journal of Neurochemistry* 1990; **54**(3)**:** 1027-1033.

78. MacKenzie RG--T, M. E. Does insulin act directly on the brain to increase tryptophan levels? *Journal of Neurochemistry* 1978; **30**(5)**:** 1205-1208.

79. Mahata SK. Effect of insulin on serotonin, 5-hydroxyindole-acetic acid, norepinephrine, epinephrine and corticosterone contents in chick. *Neuroscience Letters* 1991; **121**(1-2)**:** 115-118.

80. Orosco M--R, C.-//-Jacquot, C.-//-Gripois, D.-//-Valens, M.-//-Roffi, J. Effects of insulin on brain serotonin in the young rat: Influence of thyroid status. *Psychoneuroendocrinology* 1989; **14**(4)**:** 321-326.

81. Ruibal C--S, J. L.-//-Aldegunde, M. Brain serotonin and the control of food intake in rainbow trout (Oncorhynchus mykiss): Effects of changes in plasma glucose levels. *Journal of Comparative Physiology A: Neuroethology, Sensory, Neural, and Behavioral Physiology* 2002; **188**(6)**:** 479-484.

82. Smythe GA--B, J. E.-//-Nicholson, M. V. Rapid bidirectional effects of insulin on hypothalamic noradrenergic and serotoninergic neuronal activity in the rat: Role in glucose homeostasis. *Endocrinology* 1985; **117**(4)**:** 1590-1597.

83. Yehuda R--M, J. S. A role for serotonin in the hypothalamic-pituitary-adrenal response to insulin stress. *Neuroendocrinology* 1984; **38**(1)**:** 25-32.

84. Fernstrom JD--W, R. J. Brain serotonin content: increase following ingestion of carbohydrate diet. *Science* 1971; **174**(4013)**:** 1023-1025.

85. Gordon AE--M, B. S. Effect of insulin on brain 5-hydroxytryptamine and 5-hydroxy-indole-acetic acid of rat. *Biochem Pharmacol* 1970; **19**(12)**:** 3042-3044.

86. Jhanwar-Uniyal M--M, B.-//-Kahn, A. H. Indications of pre- and post-synaptic 5-HT1A receptor interactions in feeding behavior and neuroendocrine regulation. *Brain Research* 1994; **646**(2)**:** 247-257.

87. Azmitia EC--D, K.-//-Whitaker-Azmitia, P. M. S-100B but not NGF, EGF, insulin or calmodulin is a CNS serotonergic growth factor. *Brain Res* 1990; **516**(2)**:** 354-356.

88. Kimura H--K, K. Distribution of gamma aminobutyric acid (GABA) in the rat hypothalamus: functional correlates of GABA with activities of appetite controlling mechanisms. *Journal of Neurochemistry* 1975; **24**(5)**:** 903-907.

89. Accardi MV--B, P. M.-//-Miraucourt, L. S.-//-Orser, B. A.-//-Bowie, D. α6-Containing GABAA Receptors Are the Principal Mediators of Inhibitory Synapse Strengthening by Insulin in Cerebellar Granule Cells. *J Neurosci* 2015; **35**(26)**:** 9676-9688.

90. Saad SF. Further observations on the role of γ-aminobutyric acid in insulin-induced hypoglycaemic convulsions. *European journal of pharmacology* 1972; **17**(1)**:** 152-156.

91. Beverly JL--DV, M. G.-//-Bouman, S. D.-//-Arseneau, L. M. Noradrenergic and GABAergic systems in the medial hypothalamus are activated during hypoglycemia. *Am J Physiol Regul Integr Comp Physiol* 2001; **280**(2)**:** R563-569.

92. Tossman U--W, T.-//-Ungerstedt, U. γ-aminobutyric acid and taurine release in the striatum of the rat during hypoglycemic coma, studied by microdialysis. *Neuroscience Letters* 1985; **62**(2)**:** 231-235.

93. Hammoud H--N, O.-//-Tafreshiha, A. S.-//-Korol, S. V.-//-Jin, Z.-//-Li, J. P.-//-Birnir, B. Insulin differentially modulates GABA signalling in hippocampal neurons and, in an age-dependent manner, normalizes GABA-activated currents in the tg-APPSwe mouse model of Alzheimer's disease. *Acta Physiol (Oxf)* 2021; **232**(2)**:** e13623.

94. Jin Z--J, Y.-//-Kumar-Mendu, S.-//-Degerman, E.-//-Groop, L.-//-Birnir, B. Insulin reduces neuronal excitability by turning on GABA(A) channels that generate tonic current. *PLoS One* 2011; **6**(1)**:** e16188.

95. Wan Q--X, Z. G.-//-Man, H. Y.-//-Ackerley, C. A.-//-Braunton, J.-//-Lu, W. Y.-//-Becker, L. E.-//-MacDonald, J. F.-//-Wang, Y. T. Recruitment of functional GABA(A) receptors to postsynaptic domains by insulin. *Nature* 1997; **388**(6643)**:** 686-690.

96. Paulsen R--F, F. Comparison of results obtained with different methods for estimating GABA turnover in rat neostriatum. *Biochem Pharmacol* 1987; **36**(9)**:** 1539-1544.

97. Anitha M--A, P. M.-//-Paulose, C. S. Striatal dopamine receptors modulate the expression of insulin receptor, IGF-1 and GLUT-3 in diabetic rats: Effect of pyridoxine treatment. *European journal of pharmacology* 2012; **696**(1-3)**:** 54-61.

98. Chaves YC--G, K.-//-Stern, C. A.-//-de Oliveira Guaita, G.-//-de Souza Crippa, J. A.-//-da Cunha, J. M.-//-Zanoveli, J. M. Two-weeks treatment with cannabidiol improves biophysical and behavioral deficits associated with experimental type-1 diabetes. *Neuroscience Letters* 2020; **729**.

99. Chu PC--L, M. T.-//-Shian, L. R.-//-Leu, S. Y. Alterations in physiologic functions and in brain monoamine content in streptozocin-diabetic rats. *Diabetes* 1986; **35**(4)**:** 481-485.

100. Di Giulio AM--T, B.-//-La Croix, R.-//-Mantegazza, P.-//-Abbracchio, M. P.-//-Cattabeni, F.-//-Gorio, A. Denervation and hyperinnervation in the nervous system of diabetic animals. II. Monoaminergic and peptidergic alterations in the diabetic encephalopathy. *Journal of Neuroscience Research* 1989; **24**(3)**:** 362-368.

101. Figlewicz DP--B, M. D.-//-McCall, A. L.-//-Szot, P. Diabetes causes differential changes in CNS noradrenergic and dopaminergic neurons in the rat: A molecular study. *Brain Research* 1996; **736**(1-2)**:** 54-60.

102. Georgy GS--N, N. N.-//-Mansour, H. A.-//-Abdallah, D. M. Cerebrolysin Ameloriates Cognitive Deficits in Type III Diabetic Rats. *PLoS ONE* 2013; **8**(6).

103. Gotoh M--L, C.-//-Yatoh, M.-//-Okabayashi, N.-//-Habu, S.-//-Hirooka, Y. Hypothalamic monoamine metabolism is different between the diabetic GK (Goto-Kakizaki) rats and streptozotocin-induced diabetic rats. *Brain Research* 2006; **1073-1074**(1)**:** 497-501.

104. Gupta D--K, Y.-//-Radhakrishnan, M. Antidepressant effects of insulin in streptozotocin induced diabetic mice: Modulation of brain serotonin system. *Physiology and Behavior* 2014; **129:** 73-78.

105. Huang CW--H, T. W.-//-Wang, Y. J.-//-Chen, K. C.-//-Pei, J. C.-//-Chuang, T. Y.-//-Lai, W. S.-//-Tsai, S. H.-//-Chu, R.-//-Chen, W. C.-//-Sheen, L. Y.-//-Takahashi, S.-//-Ding, S. T.-//-Shen, T. L. Ophiocordyceps formosana improves hyperglycemia and depression-like behavior in an STZ-induced diabetic mouse model. *BMC Complementary and Alternative Medicine* 2016; **16**(1).

106. Kolta MG--S, K. F. A.-//-Williams, B. B. Role of 5-hydroxytryptamine in the regulation of brain neuropeptides in normal and diabetic rat. *Hormone Research* 1986; **23**(2)**:** 112-121.

107. Lewczuk B--P, M.-//-Ziółkowska, N.-//-Dąbrowski, M.-//-Martniuk, K.-//-Hanuszewska, M.-//-Zielonka, Ł. Effects of streptozotocin-induced diabetes on the pineal gland in the domestic pig. *International Journal of Molecular Sciences* 2018; **19**(10).

108. Li H--Z, Y. M.-//-Lei, Y. H.-//-Xu, R. J.-//-Wang, Y. Modeling of diabetes mellitus-related depression. *Neurophysiology* 2014; **46**(1)**:** 71-78.

109. Lin LW--T, F. S.-//-Yang, W. T.-//-Lai, S. C.-//-Shih, C. C.-//-Lee, S. C.-//-Wu, C. R. Differential change in cortical and hippocampal monoamines, and behavioral patterns in streptozotocin-induced type 1 diabetes rats. *Iranian Journal of Basic Medical Sciences* 2018; **21**(10)**:** 1026-1034.

110. Lozovsky D--S, C. F.-//-Kopin, I. J. Dopamine receptor binding is increased in diabetic rats. *Science* 1981; **214**(4524)**:** 1031-1033.

111. Martin FJ--M, J. M.-//-Aldegunde, M. Long-term experimentally-induced diabetes and catecholamine metabolism in rat brain regions. *Biogenic Amines* 1995; **11**(4)**:** 305-311.

112. Montefusco O--A, M. C.-//-Missale, C. Insulin-mediated effects of glucose on dopamine metabolism. *Acta Diabetologica Latina* 1983; **20**(1)**:** 71-77.

113. Motawi TK--D, H. A.-//-Hamed, M. A.-//-El-Rigal, N. S.-//-Naser, A. F. A. A Therapeutic Insight of Niacin and Coenzyme Q10 Against Diabetic Encephalopathy in Rats. *Molecular Neurobiology* 2017; **54**(3)**:** 1601-1611.

114. O'Dell LE--N, L. A.-//-Pipkin, J. A.-//-Roman, F.-//-Torres, I.-//-Jurado, J.-//-Torres, O. V.-//-Friedman, T. C.-//-Tenayuca, J. M.-//-Nazarian, A. Enhanced nicotine self-administration and suppressed dopaminergic systems in a rat model of diabetes. *Addiction Biology* 2014; **19**(6)**:** 1006-1019.

115. Owens WA--S, R. J.-//-Galici, R.-//-Chang, X.-//-Javors, M. A.-//-Galli, A.-//-France, C. P.-//-Daws, L. C. Deficits in dopamine clearance and locomotion in hypoinsulinemic rats unmask novel modulation of dopamine transporters by amphetamine. *Journal of Neurochemistry* 2005; **94**(5)**:** 1402-1410.

116. Patterson TA--B, M. D.-//-Zavosh, A.-//-Schenk, J. O.-//-Szot, P.-//-Figlewicz, D. P.-//-Figlewicz Lattermann, D. Food deprivation decreases mRMA and activity of the rat dopamine transporter. *Neuroendocrinology* 1998; **68**(1)**:** 11-20.

117. Peeyush KT--S, B.-//-Sherin, A.-//-Anju, T. R.-//-Jes, P.-//-Paulose, C. S. Cholinergic, dopaminergic and insulin receptors gene expression in the cerebellum of streptozotocin-induced diabetic rats: Functional regulation with Vitamin D3 supplementation. *Pharmacology Biochemistry and Behavior* 2010; **95**(2)**:** 216-222.

118. Portero-Tresserra M--R-B, D.-//-Vega-Carbajal, C.-//-Guillazo-Blanch, G.-//-Vale-Martínez, A.-//-Martí-Nicolovius, M. Caloric restriction modulates the monoaminergic system and metabolic hormones in aged rats. *Scientific reports* 2020; **10**(1)**:** 19299.

119. Shankar PNE--J, A.-//-Paulose, C. S. Decreased [3H] YM-09151-2 binding to dopamine D2 receptors in the hypothalamus, brainstem and pancreatic islets of streptozotocin-induced diabetic rats. *European Journal of Pharmacology* 2007; **557**(2-3)**:** 99-105.

120. Steger RW--K, S. G. Effect of continuous versus delayed insulin replacement on sex behavior and neuroendocrine function in diabetic male rats. *Diabetes* 1990; **39**(8)**:** 942-948.

121. Trulson ME--H, C. D. Decreased brain dopamine synthesis rate and increased [3H]spiroperidol binding in streptozotocin-diabetic rats. *Journal of Neurochemistry* 1983; **40**(5)**:** 1456-1459.

122. Vaidya RJ--C, N. H.-//-Vyas, B. A.-//-Shah, S. A. Effectiveness of dipeptidyl peptidase - 4 inhibitors in streptozotocin induced neurodegenerative mice. *International Journal of Pharmaceutical Sciences and Research* 2018; **9**(3)**:** 1044-1050.

123. Yang YF--L, M. T. Brain serotonin depletion attenuates diabetogenic effects of streptozotocin. *American Journal of Physiology - Endocrinology and Metabolism* 1995; **268**(5 31-5)**:** E839-E844.

124. Barber M--K, B. S.-//-Austin, M. E.-//-Patel, K. P.-//-MohanKumar, S. M.-//-MohanKumar, P. S. Diabetes-induced neuroendocrine changes in rats: role of brain monoamines, insulin and leptin. *Brain Res* 2003; **964**(1)**:** 128-135.

125. Kamei J--S, A. Evidence for the modulation of spontaneous locomotor activity by higher serum glucose levels and/or spleen-derived factor(s) in diabetic mice. *Life Sci* 1997; **60**(19)**:** 1699-1708.

126. Kwok RP--W, E. K.-//-Juorio, A. V. The concentration of dopamine, 5-hydroxytryptamine, and some of their acid metabolites in the brain of genetically diabetic rats. *Neurochem Res* 1985; **10**(5)**:** 611-616.

127. Porter JR--S, F. Neurochemical and Hormonal Changes in Lean and Obese Zucker Rats During 48 Hours of Calorie Deprivation. Are They related? *Nutr Neurosci* 1998; **1**(3)**:** 191-204.

128. Wesselmann U--K, R. J.-//-Roerig, D. L.-//-Harder, D. R. Early effects of experimental diabetes on central catecholamine concentrations. *Brain Res Bull* 1988; **20**(5)**:** 651-654.

129. Regalia J--C, F.-//-Helke, C. Streptozotocin-induced diabetes and the neurochemistry of vagal afferent neurons. *Brain Research* 2002; **938**(1-2)**:** 7-14.

130. Cruz B--C, L. M.-//-Flores, R. J.-//-Espinoza, E. J.-//-Nazarian, A.-//-O’Dell, L. E. Insulin restores the neurochemical effects of nicotine in the mesolimbic pathway of diabetic rats. *Journal of Neurochemistry* 2021; **156**(2)**:** 200-211.

131. Kino M--Y, T.-//-Aomine, M. Simultaneous measurement of nitric oxide, blood glucose, and monoamines in the hippocampus of diabetic rat: An in vivo microdialysis study. *Neurochemistry International* 2004; **44**(2)**:** 65-73.

132. Ohtani N--O, M.-//-Sugano, T. Microdialysis study of modification of hypothalamic neurotransmitters in streptozotocin-diabetic rats. *Journal of Neurochemistry* 1997; **69**(4)**:** 1622-1628.

133. Williams JM--O, W. A.-//-Turner, G. H.-//-Saunders, C.-//-Dipace, C.-//-Blakely, R. D.-//-France, C. P.-//-Gore, J. C.-//-Daws, L. C.-//-Avison, M. J.-//-Galli, A. Hypoinsulinemia regulates amphetamine-induced reverse transport of dopamine. *PLoS Biol* 2007; **5**(10)**:** e274.

134. Jayanarayanan S--S, S.-//-Peeyush, K. T.-//-Anju, T. R.-//-Paulose, C. S. NMDA and AMPA receptor mediated excitotoxicity in cerebral cortex of streptozotocin induced diabetic rat: Ameliorating effects of curcumin. *Chemico-Biological Interactions* 2013; **201**(1-3)**:** 39-48.

135. Viswaprakash N--V, T.-//-Viswaprakash, A.-//-Judd, R.-//-Parameshwaran, K.-//-Suppiramaniam, V. Insulin treatment restores glutamate (α-amino-3-hydroxy-5-methyl-4-isoxazolepropionic acid) receptor function in the hippocampus of diabetic rats. *Journal of Neuroscience Research* 2015; **93**(9)**:** 1442-1450.

136. Amin SN--Y, S. M.-//-Youssef, M. F.-//-Rashed, L. A.-//-Mohamady, I. A histological and functional study on hippocampal formation of normal and diabetic rats. *F1000Res* 2013; **2:** 151.

137. Balakrishnan S--T, P. K.-//-Paulose, C. S. Glutamate (mGluR-5) gene expression in brain regions of streptozotocin induced diabetic rats as a function of age: role in regulation of calcium release from the pancreatic islets in vitro. *J Biomed Sci* 2009; **16**(1)**:** 99.

138. Cardoso S--C, C.-//-Santos, R.-//-Correia, S.-//-Santos, M. S.-//-Seiça, R.-//-Oliveira, C. R.-//-Moreira, P. I. Impact of STZ-induced hyperglycemia and insulin-induced hypoglycemia in plasma amino acids and cortical synaptosomal neurotransmitters. *Synapse* 2011; **65**(6)**:** 457-466.

139. Coleman ES--D, J. C.-//-Braden, T. D.-//-Judd, R. L.-//-Posner, P. Insulin treatment prevents diabetes-induced alterations in astrocyte glutamate uptake and GFAP content in rats at 4 and 8 weeks of diabetes duration. *Brain Res* 2010; **1306:** 131-141.

140. Di Luca M--R, L.-//-Gardoni, F.-//-Cattabeni, F.-//-Biessels, G. J.-//-Gispen, W. H. NMDA receptor subunits are modified transcriptionally and post-translationally in the brain of streptozotocin-diabetic rats. *Diabetologia* 1999; **42**(6)**:** 693-701.

141. Gardoni F--K, A.-//-Bellone, C.-//-Biessels, G. J.-//-Ramakers, G. M.-//-Cattabeni, F.-//-Gispent, W. H.-//-Di Luca, M. Effects of streptozotocin-diabetes on the hippocampal NMDA receptor complex in rats. *J Neurochem* 2002; **80**(3)**:** 438-447.

142. Hawkins RA--M, A.-//-Dejoseph, M. R.-//-Viña, J. R.-//-Fernstrom, J. D. Glutamate permeability at the blood-brain barrier in insulinopenic and insulin-resistant rats. *Metabolism* 2010; **59**(2)**:** 258-266.

143. Jangra A--D, A. K.-//-Khandwe, S.-//-Sharma, S. S. Amelioration of diabetes-induced neurobehavioral and neurochemical changes by melatonin and nicotinamide: implication of oxidative stress-PARP pathway. *Pharmacol Biochem Behav* 2013; **114-115:** 43-51.

144. Nair AR--B, M. P.-//-Paulose, C. S. Effect of pyridoxine and insulin administration on brain glutamate dehydrogenase activity and blood glucose control in streptozotocin-induced diabetic rats. *Biochim Biophys Acta* 1998; **1381**(3)**:** 351-354.

145. Nair P--P, P. S.-//-Abraham, A.-//-Sudha, B.-//-Raghu, K. G.-//-Paulose, C. S. Glutamate dehydrogenase induction in the brain of streptozotocin diabetic rats. *Indian J Biochem Biophys* 1996; **33**(5)**:** 428-430.

146. Nardin P--Z, C.-//-Hansen, F.-//-Batassini, C.-//-Gasparin, M. S.-//-Sesterheim, P.-//-Gonçalves, C. A. Peripheral Levels of AGEs and Astrocyte Alterations in the Hippocampus of STZ-Diabetic Rats. *Neurochem Res* 2016; **41**(8)**:** 2006-2016.

147. Shinbori C--S, Y.-//-Mitani, H.-//-Saito, M.-//-Satoh, K. Effects of cyclohexanonic long-chain fatty alcohol, tCFA15 on amino acids in diabetic rat brain: a preliminary study. *Neurochem Res* 2008; **33**(7)**:** 1189-1195.

148. Suzuki M--S, J.-//-Furuya, S.-//-Mita, M.-//-Hamase, K.-//-Aiso, S. Type 1 diabetes mellitus in mice increases hippocampal D-serine in the acute phase after streptozotocin injection. *Brain Res* 2012; **1466:** 167-176.

149. Valastro B--C, J.-//-Lavoie, N.-//-Gagnon, S.-//-Trudeau, F.-//-Massicotte, G. Up-regulation of glutamate receptors is associated with LTP defects in the early stages of diabetes mellitus. *Diabetologia* 2002; **45**(5)**:** 642-650.

150. Zhang H--H, M.-//-Gao, L.-//-Lei, H. Region-specific cerebral metabolic alterations in streptozotocin-induced type 1 diabetic rats: an in vivo proton magnetic resonance spectroscopy study. *J Cereb Blood Flow Metab* 2015; **35**(11)**:** 1738-1745.

151. Abraham PM--A, T. R.-//-Jayanarayanan, S.-//-Paulose, C. S. Serotonergic receptor upregulation in cerebral cortex and down regulation in brainstem of streptozotocin induced diabetic rats: Antagonism by pyridoxine and insulin. *Neuroscience Letters* 2010; **483**(1)**:** 23-27.

152. Abraham PM--K, K. P.-//-Mathew, J.-//-Malat, A.-//-Joy, S.-//-Paulose, C. S. Alterations in hippocampal serotonergic and INSR function in streptozotocin induced diabetic rats exposed to stress: Neuroprotective role of pyridoxine and Aegle marmelose. *Journal of Biomedical Science* 2010; **17**(1).

153. Atienza G--M, J. M.-//-Martin, F. J.-//-Aldegunde, M. Differential changes in serotonin metabolism in different brain regions of streptozotocin-diabetic rats. *Biogenic Amines* 1995; **11**(2)**:** 123-135.

154. Chaouloff F--L, D.-//-Merino, D.-//-Serrurier, B.-//-Baudrie, V.-//-Elghozi, J. L. Duration of streptozotocin diabetes influences the response of hypothalamic serotonin metabolism to immobilization stress. *Neuroendocrinology* 1989; **50**(3)**:** 344-350.

155. Crandall EA--F, J. D. Acute changes in brain tryptophan and serotonin after carbohydrate or protein ingestion by diabetic rats. *Diabetes* 1980; **29**(6)**:** 460-466.

156. Kohsaka S--T, K.-//-Tsukada, Y. Effect of food restriction on serotonin metabolism in rat brain. *Neurochemical Research* 1980; **5**(1)**:** 69-79.

157. Ramakrishnan R--P, K.-//-Jayakumar, A. R.-//-Gunasekaran, P.-//-Sheeladevi, R.-//-Suthanthirarajan, N. Involvement of Ca2+/calmodulin-dependent protein kinase II in the modulation of indolamines in diabetic and hyperglycemic rats. *Journal of Neuroscience Research* 2005; **80**(4)**:** 518-528.

158. Ramakrishnan R--S, R.-//-Namasivayam, A. Regulation of protein kinases and coregulatory interplay of S-100β and serotonin transporter on serotonin levels in diabetic rat brain. *Journal of Neuroscience Research* 2009; **87**(1)**:** 246-259.

159. Woodger TL--S, A.-//-Anderson, G. H. Diabetes, dietary tryptophan, and protein intake regulation in weanling rats. *American Journal of Physiology - Regulatory Integrative and Comparative Physiology* 1979; **5**(3)**:** R307-R311.

160. Herrera R--M, G.-//-Hernandez, J. Inhibition and kinetic changes of brain tryptophan-5-hydroxylase during insulin-dependent diabetes mellitus in the rat. *Nutr Neurosci* 2005; **8**(1)**:** 57-62.

161. Jackson J--P, C. S. Enhancement of [m-methoxy 3H]MDL100907 binding to 5HT(2A) receptors in cerebral cortex and brain stem of streptozotocin induced diabetic rats. *Molecular and Cellular Biochemistry* 1999; **199**(1-2)**:** 81-85.

162. James J--P, C. S. Brain 5HT2A receptor regulation by tryptophan supplementation in Streptozotocin diabetic rats. *Journal of Biochemistry, Molecular Biology and Biophysics* 2001; **5**(1)**:** 1-7.

163. Ramakrishnan R--S, R.-//-Suthanthirarajan, N. PKC-α mediated alterations of indoleamine contents in diabetic rat brain. *Brain Research Bulletin* 2004; **64**(2)**:** 189-194.

164. Miyata S--Y, N.-//-Hirano, S.-//-Tanaka, S. i-//-Kamei, J. Diabetes attenuates psychological stress-elicited 5-HT secretion in the prefrontal cortex but not in the amygdala of mice. *Brain Research* 2007; **1147**(1)**:** 233-239.

165. Boychuk CR--H, K. C.-//-Smith, B. N. Diabetes induces GABA receptor plasticity in murine vagal motor neurons. *J Neurophysiol* 2015; **114**(1)**:** 698-706.

166. Boychuk CR--S, B. N. Glutamatergic drive facilitates synaptic inhibition of dorsal vagal motor neurons after experimentally induced diabetes in mice. *J Neurophysiol* 2016; **116**(3)**:** 1498-1506.

167. Boychuk CR--S, K. C.-//-Smith, B. N. Functional and molecular plasticity of γ and α1 GABA(A) receptor subunits in the dorsal motor nucleus of the vagus after experimentally induced diabetes. *J Neurophysiol* 2017; **118**(5)**:** 2833-2841.

168. Chan O--P, S. A.-//-Horblitt, A.-//-Zhu, W.-//-Sherwin, R. S. Lactate-induced release of GABA in the ventromedial hypothalamus contributes to counterregulatory failure in recurrent hypoglycemia and diabetes. *Diabetes* 2013; **62**(12)**:** 4239-4246.

169. van der Zwaal EM--dW, B. A.-//-van de Giessen, E. M.-//-Janssen, I.-//-Berends, F. J.-//-van de Laar, A.-//-Ackermans, M. T.-//-Fliers, E.-//-la Fleur, S. E.-//-Booij, J.-//-Serlie, M. J. Striatal dopamine D2/3 receptor availability increases after long-term bariatric surgery-induced weight loss. *European Neuropsychopharmacology* 2016; **26**(7)**:** 1190-1200.

170. Versteeg RI--K, K. E.-//-Booij, J.-//-Ackermans, M. T.-//-Unmehopa, U. A.-//-Fliers, E.-//-La Fleur, S. E.-//-Serlie, M. J. Serotonin Transporter Binding in the Diencephalon Is Reduced in Insulin-Resistant Obese Humans. *Neuroendocrinology* 2017; **105**(2)**:** 141-149.

171. Fried PJ--P-L, A.-//-Bolo, N. R. Diabetes and the link between neuroplasticity and glutamate in the aging human motor cortex. *Clin Neurophysiol* 2019; **130**(9)**:** 1502-1510.

172. Haley AP--G, M. M.-//-Tarumi, T.-//-Miles, S. C.-//-Goudarzi, K.-//-Tanaka, H. Elevated cerebral glutamate and myo-inositol levels in cognitively normal middle-aged adults with metabolic syndrome. *Metab Brain Dis* 2010; **25**(4)**:** 397-405.

173. Thielen JW--G, S.-//-Hong, D.-//-Rohani Rankouhi, S.-//-Chen, B.-//-Apostolopoulou, M.-//-Anadol-Schmitz, E.-//-Roden, M.-//-Norris, D. G.-//-Tendolkar, I. Higher GABA concentration in the medial prefrontal cortex of Type 2 diabetes patients is associated with episodic memory dysfunction. *Hum Brain Mapp* 2019; **40**(14)**:** 4287-4295.

174. Edwin Thanarajah S--I, S.-//-Kuzmanovic, B.-//-Rigoux, L.-//-Stephan, K. E.-//-Brüning, J. C.-//-Tittgemeyer, M. Modulation of midbrain neurocircuitry by intranasal insulin. *Neuroimage* 2019; **194:** 120-127.

175. Anderzhanova E--C, M.-//-Hajnal, A. Altered basal and stimulated accumbens dopamine release in obese OLETF rats as a function of age and diabetic status. *American Journal of Physiology - Regulatory Integrative and Comparative Physiology* 2007; **293**(2)**:** R603-R611.

176. Cumming P--M, S.-//-Riss, P. J.-//-Grill, E.-//-Pischetsrieder, M.-//-Kuwert, T.-//-Prante, O. Perturbed Development of Striatal Dopamine Transporters in Fatty Versus Lean Zucker Rats: a Follow-up Small Animal PET Study. *Molecular Imaging and Biology* 2015; **17**(4)**:** 521-528.

177. de Leeuw van Weenen JE--H, L.-//-Jansen-Van Zelm, K.-//-de Vries, M. G.-//-Tamsma, J. T.-//-Romijn, J. A.-//-Pijl, H. Four weeks high fat feeding induces insulin resistance without affecting dopamine release or gene expression patterns in the hypothalamus of C57Bl6 mice. *Brain Research* 2009; **1250:** 141-148.

178. Figlewicz Latteman DP--P, T. A.-//-Johnson, L. B.-//-Zavosh, A.-//-Israel, P. A.-//-Szot, P. Dopamine transporter mRNA is increased in the CNS of Zucker fatty (fa/fa) rats. *Brain Research Bulletin* 1998; **46**(3)**:** 199-202.

179. Morris JK--B, G. L.-//-Gorres, B. K.-//-Davis, V. A.-//-Kim, J.-//-Lee, P. P.-//-Brooks, W. M.-//-Gerhardt, G. A.-//-Geiger, P. C.-//-Stanford, J. A. Insulin resistance impairs nigrostriatal dopamine function. *Experimental Neurology* 2011; **231**(1)**:** 171-180.

180. Paternain L--B, M. A.-//-De La Garza, A. L.-//-Milagro, F. I.-//-Martínez, J. A.-//-Campión, J. Transcriptomic and epigenetic changes in the hypothalamus are involved in an increased susceptibility to a high-fat-sucrose diet in prenatally stressed female rats. *Neuroendocrinology* 2012; **96**(3)**:** 249-260.

181. Routh VH--S, J. S.-//-Horwitz, B. A. Serotonergic activity is depressed in the ventromedial hypothalamic nucleus of 12-day-old obese Zucker rats. *American Journal of Physiology - Regulatory Integrative and Comparative Physiology* 1994; **267**(3 36-3)**:** R712-R719.

182. Speed N--S, C.-//-Davis, A. R.-//-Owens, W. A.-//-Matthies, H. J. G.-//-Saadat, S.-//-Kennedy, J. P.-//-Vaughan, R. A.-//-Neve, R. L.-//-Lindsley, C. W.-//-Russo, S. J.-//-Daws, L. C.-//-Niswender, K. D.-//-Galli, A. Impaired striatal akt signaling disrupts dopamine homeostasis and increases feeding. *PLoS ONE* 2011; **6**(9).

183. Xin X--Y, Z.-//-Yang, H. Immediate-early c-fos and tyrosine hydroxylase protein expression in different brain regions in high-fat induced obesity in mice. *Zhongguo ying yong sheng li xue za zhi = Zhongguo yingyong shenglixue zazhi = Chinese journal of applied physiology* 2017; **33**(6)**:** 572-576.

184. Adams WK--S, J. L.-//-Kaur, S.-//-D'Souza A, M.-//-Kieffer, T. J.-//-Winstanley, C. A. Long-term, calorie-restricted intake of a high-fat diet in rats reduces impulse control and ventral striatal D2 receptor signalling - two markers of addiction vulnerability. *Eur J Neurosci* 2015; **42**(12)**:** 3095-3104.

185. Bittencourt A--B, P. O.-//-Ribeiro, C. T.-//-Gasparotto, J.-//-Bortolin, R. C.-//-de Vargas, A. R.-//-Heimfarth, L.-//-de Almeida, R. F.-//-Moreira, J. C. F.-//-de Oliveira, J.-//-Gelain, D. P. High fat diet-induced obesity causes a reduction in brain tyrosine hydroxylase levels and non-motor features in rats through metabolic dysfunction, neuroinflammation and oxidative stress. *Nutr Neurosci* 2020**:** 1-15.

186. D'Souza SS--A, A. High-fat simple carbohydrate feeding impairs central and peripheral monoamine metabolic pathway triggering the onset of metabolic syndrome in C57Bl/6J mice. *Neurol India* 2016; **64**(5)**:** 923-933.

187. Jones KT--W, C.-//-Zhen, J.-//-Antonio, T.-//-Carr, K. D.-//-Reith, M. E. Effects of diet and insulin on dopamine transporter activity and expression in rat caudate-putamen, nucleus accumbens, and midbrain. *J Neurochem* 2017; **140**(5)**:** 728-740.

188. Mechlovich D--A, T.-//-Bar-Am, O.-//-Weinreb, O.-//-Youdim, M. B. Molecular targets of the multifunctional iron-chelating drug, M30, in the brains of mouse models of type 2 diabetes mellitus. *Br J Pharmacol* 2014; **171**(24)**:** 5636-5649.

189. Sharma S--T, R. High fat diet feeding exacerbates the toxic effects of 6-hydroxydopamine in rats: Possible involvement of histone acetylation. *Parkinsonism and Related Disorders* 2016; **22:** e181.

190. Auer MK--S, M.-//-Lenz, J. N.-//-Jakovcevski, M.-//-Biedermann, S. V.-//-Falfán-Melgoza, C.-//-Deussing, J.-//-Steinle, J.-//-Bielohuby, M.-//-Bidlingmaier, M.-//-Pfister, F.-//-Stalla, G. K.-//-Ende, G.-//-Weber-Fahr, W.-//-Fuss, J.-//-Gass, P. Effects of a high-caloric diet and physical exercise on brain metabolite levels: a combined proton MRS and histologic study. *J Cereb Blood Flow Metab* 2015; **35**(4)**:** 554-564.

191. Duarte JM--A, P. M.-//-Carvalho, R. A.-//-Cunha, R. A. Caffeine consumption prevents diabetes-induced memory impairment and synaptotoxicity in the hippocampus of NONcZNO10/LTJ mice. *PLoS One* 2012; **7**(4)**:** e21899.

192. Girault FM--S, S.-//-Gruetter, R.-//-Duarte, J. M. N. Alterations of Brain Energy Metabolism in Type 2 Diabetic Goto-Kakizaki Rats Measured In Vivo by (13)C Magnetic Resonance Spectroscopy. *Neurotox Res* 2019; **36**(2)**:** 268-278.

193. Jin L--L, Y. P.-//-Feng, Q.-//-Ren, L.-//-Wang, F.-//-Bo, G. J.-//-Wang, L. Cognitive deficits and Alzheimer-like neuropathological impairments during adolescence in a rat model of type 2 diabetes mellitus. *Neural Regen Res* 2018; **13**(11)**:** 1995-2004.

194. Kawamura N--K, G.-//-Yamada-Goto, N.-//-Novianti, E.-//-Inui, A.-//-Asakawa, A. Impaired brain fractalkine-CX3CR1 signaling is implicated in cognitive dysfunction in diet-induced obese mice. *BMJ Open Diabetes Res Care* 2021; **9**(1).

195. Liu X--Z, H. Leptin-Mediated Sympathoexcitation in Obese Rats: Role for Neuron-Astrocyte Crosstalk in the Arcuate Nucleus. *Front Neurosci* 2019; **13:** 1217.

196. Lizarbe B--S, A. F.-//-Larsson, S.-//-Duarte, J. M. N. Neurochemical Modifications in the Hippocampus, Cortex and Hypothalamus of Mice Exposed to Long-Term High-Fat Diet. *Front Neurosci* 2018; **12:** 985.

197. Matsunaga Y--N, T.-//-Hatakeyama, A.-//-Kawagoe, Y.-//-Sawano, E.-//-Tashiro, T. Impairment of synaptic development in the hippocampus of diabetic Goto-Kakizaki rats. *Int J Dev Neurosci* 2016; **53:** 58-67.

198. Sasaki-Hamada S--H, Y.-//-Koyama, H.-//-Otsuka, H.-//-Oka, J. Changes in hippocampal synaptic functions and protein expression in monosodium glutamate-treated obese mice during development of glucose intolerance. *Eur J Neurosci* 2015; **41**(11)**:** 1393-1401.

199. Spinelli M--F, S.-//-Mainardi, M.-//-Scala, F.-//-Natale, F.-//-Lapenta, R.-//-Mattera, A.-//-Rinaudo, M.-//-Li Puma, D. D.-//-Ripoli, C.-//-Grassi, A.-//-D'Ascenzo, M.-//-Grassi, C. Brain insulin resistance impairs hippocampal synaptic plasticity and memory by increasing GluA1 palmitoylation through FoxO3a. *Nat Commun* 2017; **8**(1)**:** 2009.

200. Tsai SF--W, H. T.-//-Chen, P. C.-//-Chen, Y. W.-//-Yu, M.-//-Wang, T. F.-//-Wu, S. Y.-//-Tzeng, S. F.-//-Kuo, Y. M. High-fat diet suppresses the astrocytic process arborization and downregulates the glial glutamate transporters in the hippocampus of mice. *Brain Res* 2018; **1700:** 66-77.

201. Duarte AI--S, M. S.-//-Seiça, R.-//-Oliveira, C. R. Oxidative stress affects synaptosomal γ-aminobutyric acid and glutamate transport in diabetic rats: The role of insulin. *Diabetes* 2004; **53**(8)**:** 2110-2116.

202. Duarte A--S, M.-//-Seiça, R.-//-Resende de Oliveira, C. Effect of oxidative stress on the uptake of GABA and glutamate in synaptosomes isolated from diabetic rat brain. *Neuroendocrinology* 2000; **72**(3)**:** 179-186.

203. Okuda MH--Z, J. C. S.-//-de Santana, A. A.-//-Santamarina, A. B.-//-Moreno, M. F.-//-Hachul, A. C. L.-//-Santos, B. D.-//-Nascimento, C. M. O. D.-//-Ribeiro, E. B.-//-Oyama, L. M. Green tea extract improves high fat diet-induced hypothalamic inflammation, without affecting the serotoninergic system. *Journal of Nutritional Biochemistry* 2014; **25**(10)**:** 1084-1089.

204. Zemdegs J--M, H.-//-Pintana, H.-//-Bullich, S.-//-Manta, S.-//-Marqués, M. A.-//-Moro, C.-//-Laye, S.-//-Ducrocq, F.-//-Chattipakorn, N.-//-Chattipakorn, S. C.-//-Rampon, C.-//-Pénicaud, L.-//-Fioramonti, X.-//-Guiard, B. P. Metformin promotes anxiolytic and antidepressant-like responses in insulin-resistant mice by decreasing circulating branched-chain amino acids. *Journal of Neuroscience* 2019; **39**(30)**:** 5935-5948.

205. McNeilly AD--S, C. A.-//-Sutherland, C.-//-Balfour, D. J. High fat feeding is associated with stimulation of the hypothalamic-pituitary-adrenal axis and reduced anxiety in the rat. *Psychoneuroendocrinology* 2015; **52:** 272-280.

206. Tu LL--S, Q.-//-Wei, L. L.-//-Shi, J.-//-Li, J. P. Upregulation of GABA receptor promotes long-term potentiation and depotentiation in the hippocampal CA1 region of mice with type 2 diabetes mellitus. *Exp Ther Med* 2019; **18**(4)**:** 2429-2436.

207. Cai W--X, C.-//-Sakaguchi, M.-//-Konishi, M.-//-Shirazian, A.-//-Ferris, H. A.-//-Li, M. E.-//-Yu, R.-//-Kleinridders, A.-//-Pothos, E. N.-//-Kahn, C. R. Insulin regulates astrocyte gliotransmission and modulates behavior. *Journal of Clinical Investigation* 2018; **128**(7)**:** 2914-2926.

208. Kleinridders A--C, W.-//-Cappellucci, L.-//-Ghazarian, A.-//-Collins, W. R.-//-Vienberg, S. G.-//-Pothos, E. N.-//-Kahn, C. R. Insulin resistance in brain alters dopamine turnover and causes behavioral disorders. *Proceedings of the National Academy of Sciences of the United States of America* 2015; **112**(11)**:** 3463-3468.

209. Salkovic M--S, I.-//-Lackovic, Z. Striatal dopaminergic D1 and D2 receptors after intracerebroventricular application of alloxan and streptozocin in rat. *Journal of Neural Transmission - General Section* 1995; **100**(2)**:** 137-145.

210. Abdel Rasheed NO--ES, N. S.-//-El-Khatib, A. S. Targeting central β2 receptors ameliorates streptozotocin-induced neuroinflammation via inhibition of glycogen synthase kinase3 pathway in mice. *Prog Neuropsychopharmacol Biol Psychiatry* 2018; **86:** 65-75.

211. Costello DA--C, M.-//-Al-Qassab, H.-//-Plattner, F.-//-Irvine, E. E.-//-Choudhury, A. I.-//-Giese, K. P.-//-Withers, D. J.-//-Pedarzani, P. Brain deletion of insulin receptor substrate 2 disrupts hippocampal synaptic plasticity and metaplasticity. *PLoS One* 2012; **7**(2)**:** e31124.

212. Grillo CA--P, G. G.-//-Lawrence, R. C.-//-Wrighten, S. A.-//-Green, A. J.-//-Wilson, S. P.-//-Sakai, R. R.-//-Kelly, S. J.-//-Wilson, M. A.-//-Mott, D. D.-//-Reagan, L. P. Hippocampal Insulin Resistance Impairs Spatial Learning and Synaptic Plasticity. *Diabetes* 2015; **64**(11)**:** 3927-3936.

213. Labak M--F, T.-//-Kirk, D.-//-Rushforth, D.-//-Tomanek, B.-//-Jasiński, A.-//-Grieb, P. Metabolic changes in rat brain following intracerebroventricular injections of streptozotocin: a model of sporadic Alzheimer's disease. *Acta Neurochir Suppl* 2010; **106:** 177-181.

214. Murtishaw AS--H, C. F.-//-Bolton, M. M.-//-Sabbagh, J. J.-//-Langhardt, M. A.-//-Kinney, J. W. Effect of acute lipopolysaccharide-induced inflammation in intracerebroventricular-streptozotocin injected rats. *Neuropharmacology* 2016; **101:** 110-122.

215. Shonesy BC--T, K.-//-Parameshwaran, K.-//-Rahman, E. A.-//-Karuppagounder, S. S.-//-Huggins, K. W.-//-Pinkert, C. A.-//-Amin, R.-//-Dhanasekaran, M.-//-Suppiramaniam, V. Central insulin resistance and synaptic dysfunction in intracerebroventricular-streptozotocin injected rodents. *Neurobiol Aging* 2012; **33**(2)**:** 430.e435-418.
